# Shallow defects and variable photoluminescence decay times up to 280 µs in triple-cation perovskites

**DOI:** 10.1038/s41563-023-01771-2

**Published:** 2024-01-09

**Authors:** Ye Yuan, Genghua Yan, Chris Dreessen, Toby Rudolph, Markus Hülsbeck, Benjamin Klingebiel, Jiajiu Ye, Uwe Rau, Thomas Kirchartz

**Affiliations:** 1 IEK-5 Photovoltaik, Forschungszentrum Jülich, Jülich, Germany; 2 Institute for Solar Energy Systems, Guangdong Provincial Key Laboratory of Photovoltaic Technology, School of Physics, Sun Yat-sen University, Guangzhou, P. R. China; 3 Instituto de Ciencia Molecular (ICMol), Universidad de Valencia, Paterna, Spain; 4 Faculty of Engineering and CENIDE, University of Duisburg-Essen, Duisburg, Germany

**Keywords:** Photonic devices, Solar cells

## Abstract

Quantifying recombination in halide perovskites is a crucial prerequisite to control and improve the performance of perovskite-based solar cells. While both steady-state and transient photoluminescence are frequently used to assess recombination in perovskite absorbers, quantitative analyses within a consistent model are seldom reported. We use transient photoluminescence measurements with a large dynamic range of more than ten orders of magnitude on triple-cation perovskite films showing long-lived photoluminescence transients featuring continuously changing decay times that range from tens of nanoseconds to hundreds of microseconds. We quantitatively explain both the transient and steady-state photoluminescence with the presence of a high density of shallow defects and consequent high rates of charge carrier trapping, thereby showing that deep defects do not affect the recombination dynamics. The complex carrier kinetics caused by emission and recombination processes via shallow defects imply that the reporting of only single lifetime values, as is routinely done in the literature, is meaningless for such materials. We show that the features indicative for shallow defects seen in the bare films remain dominant in finished devices and are therefore also crucial to understanding the performance of perovskite solar cells.

## Abstract (web summary)

Quantifying recombination in halide perovskites is crucial, but quantitative analysis remains rare. Here the authors observe a long-lived and continuously changing photoluminescence decay time due to the high density of shallow defects and substantial rates of charge carrier trapping.

## Main

Non-radiative recombination via defects is one of the most important loss processes in most photovoltaic technologies^1,2^. Thus, a considerable amount of photovoltaic research has been dedicated to suppressing non-radiative recombination as well as characterizing and quantifying its extent^3,4^. This is especially true for emerging photovoltaic technologies such as halide perovskites that mostly rely on solution-processed polycrystalline thin films. In lead halide perovskites, non-radiative recombination is much less of a problem compared to other polycrystalline materials used for photovoltaic applications^5,6^. The common explanation is that most intrinsic defects are either shallow or unlikely to form^7^. Interestingly, the experimental community has so far worked under the paradigm that deep defects dominate recombination, whereas shallow defects are mostly considered irrelevant^8,9^. The only shallow defects considered crucial for device functionality are mobile ions causing field screening and hysteresis in the current–voltage curve^10,11^.

**Fig. 1:**
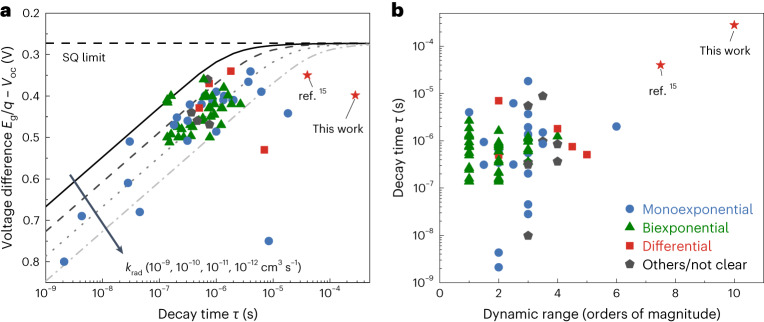
Meta-analysis of reported energy loss and decay time in publications. **a**, The voltage difference *E*_g_/*q* – *V*_oc_ as a function of decay time of perovskite films. The lines indicate the relationship between carrier lifetime and energy loss^13,14^, which is calculated based on a step function absorptance by taking *p*_0_ = 0, *p*_a_ = 0, *p*_e_ = 0.05, *G*_ext_ = 5.3 × 10^21^ cm^−3^ s^−1^ and ${V}_{{\rm{oc}}}^{{\rm{SQ}}}=1.297 \ {\rm{V}}$ (corresponding to a bandgap of 1.57 eV), where *p*_0_ is the equilibrium carrier concentration, *p*_a_ is parasitic absorption probability, *p*_e_ is emission probability, *G*_ext_ is the generation rate of electron-hole pairs due to external illumination and ${V}_{{\rm{oc}}}^{{\rm{SQ}}}$ is the open-circuit voltage in the Shockley-Queisser (SQ) model. Additionally, *k*_rad_ is the radiative recombination coefficient. **b**, Meta-analysis on the dynamic range of tr-PL decay curves in publications. The colours represent the fitting methods of the tr-PL decay curves. More information is in Supplementary Note 5. Source data

Identifying the properties of the defects dominating non-radiative recombination is important for various reasons. Depending on the dominant defect species, different material optimization strategies and characterization approaches are needed. For deep traps, both transient photoluminescence (PL) and PL quantum yields are viable methods to quantify recombination, and the information content of both quantities is basically identical^12^. In the presence of deep traps, transient PL measurements lead to monoexponential decays at sufficiently low injection conditions, from which charge carrier lifetimes can be extracted. Those lifetimes must then be consistent with the PL quantum yields obtained from steady-state PL measurements and consequently correlate with the voltage difference *E*_g_/*q* – *V*_oc_, where *E*_g_, *q* and *V*_oc_ are the bandgap energy, elementary charge and open-circuit voltage, respectively. Figure 1a shows this correlation for a range of perovskite studies^13,14^, where the voltage difference was calculated from the solar cell data while the lifetime was obtained from film measurements. While many data points seem to show correlation between lifetime and voltage difference, others (especially the data points (stars) labelled ‘this work’ and ‘ref. ^15^’) feature decay times that seem too long for the associated value of *E*_g_/*q* – *V*_oc_. This raises the question of whether transient PL decay times are always a valid method to quantify recombination and voltage losses in halide perovskites. Moreover, the finding raises doubts regarding the implicit assumption that deep defects dominate recombination losses and transient PL decay.

Here we show that typical triple-cation perovskite layers, layer stacks and solar cells are strongly affected by shallow defects that manifest themselves in steady-state and transient PL data. The most convincing evidence for shallow defects is the presence of an extremely long-lived PL signal in time-resolved PL (tr-PL) measurements with a high dynamic range of greater than ten orders of magnitude. Combined with the observation of PL quantum yields in the range of 2% (this means much smaller than unity) in films, we can rule out radiative band-to-band recombination as the reason for the long-lived decay. Consistent with the dominant influence of shallow defects, the decay approximately follows a power law (PL flux *ϕ* ∝ *t*^-*α*^, where *t* is time and *α* is a constant) over the investigated time range and never saturates to an exponential decay (Supplementary Fig. 5). The high dynamic range is important to disentangle different mechanisms in large-signal transients^16^ and is not typically found in the literature on halide perovskites (Fig. 1b). The differential decay times exceed 100 µs at the end of the decay, which implies that these decays may be the longest measured so far in halide perovskites or any other direct semiconductor considered for photovoltaic applications^6^. The high dynamic range combined with the power-law nature of the decay allows us to observe differential decay times that vary over four orders of magnitude spanning tens of nanoseconds at the beginning of a decay up to hundreds of microseconds. This implies that the heavily used concept of a single ‘lifetime’ of charge carriers in halide perovskites can be highly misleading and may have to be replaced by effective recombination coefficients. We note that decay times exceeding tens of microseconds are observed also in films with one or two charge-extracting layers and in full devices. This finding implies that the shallow traps and long lifetimes are consistent with efficient charge extraction in solar cells with fill factors exceeding 80%. The absence of any type of saturation of the decay time to a constant value shows that (1) the halide perovskite films are extremely intrinsic^17^ and (2) the Shockley–Read–Hall (SRH) lifetime for recombination via deep defects must be extremely long (in the range of hundreds of microseconds; Supplementary Fig. 7). The latter finding implies a change of the dominant paradigm that reduction of deep defects is crucial for further efficiency improvements. Instead, we show that a high density of shallow defects dominates recombination and limits device performance.

## Defect-mediated recombination

Recombination via defects is the most relevant recombination mechanism for thin-film photovoltaics as it reduces the open-circuit voltage of solar cells and often also the fill factor and the short-circuit current. The SRH model is used to identify non-radiative recombination and estimate its effect on device performance. The SRH recombination rate for one species of singly charged defects is given by^18,19^

1 $${R}_{{\rm{SRH}}}=\frac{\left({np}-{n}_{{\mathrm{i}}}^{2}\right)}{\left(n+{n}_{1}\right){\tau }_{{\rm{p}}}+(p+{p}_{1}){\tau }_{{\rm{n}}}},$$

where *n*, *p* and *n*_i_ represent electron, hole and intrinsic carrier concentrations; *τ*_p_ and *τ*_n_ are the SRH lifetimes for holes and electrons; *n*_1_ = *N*_C_exp[(*E*_T_ – *E*_C_)/*k*_B_*T*]; and *p*_1_ = *N*_V_exp[(*E*_V_ – *E*_T_)/*k*_B_*T*]. Here *n*_1_ and *p*_1_ are in the unit ‘per cubic centimetre’ and use further variables such as the effective density of states for the conduction and valence bands (*N*_C_ and *N*_V_, respectively) and the energy of the trap (*E*_T_), conduction band edge (*E*_C_) and valence band edge (*E*_V_), as well as a Boltzmann constant (*k*_B_) and temperature (*T*). Given that lead halide perovskites behave like intrinsic semiconductors^17^, the equation is typically simplified using the two assumptions *n* = *p* and *n* ≫ *n*_i_. Furthermore, *n*_1_ and *p*_1_ are typically considered negligible relative to *n* and *p*, implying that detrapping is neglected, which is typically a good approximation for a deep trap. These simplifications lead to *R*_SRH_ = *n*/(*τ*_p_ + *τ*_n_), that is, to the situation in which SRH recombination is often considered synonymous with first-order recombination, which has a rate that is linear in *n* (and *p*), that is, *R*_SRH_ ∝ *n*^*δ*^, where *δ* = 1 is the reaction order. However, this is only a special case, where the trap is between the quasi-Fermi levels under operation^20^. If a trap dominating recombination is close to either the conduction or the valence band edge, one of the two voltage-independent terms *n*_1_ or *p*_1_ will become comparable to *n* and *p*, and hence affect the recombination rate. Without loss of generality, we assume that we have a defect close to the conduction band, implying that *n*_1_ ≫ *p*_1_. In this case, the rate *R*_SRH_ = *n*^2^/[(*n* + *n*_1_)*τ*_p_ + *nτ*_n_] can scale linearly with *n* (for the case of *n*_1_ ≪ *n*); it may scale quadratically with *n* (for *n*_1_ ≫ *n*); or it may have a non-integer recombination order if *n* and *n*_1_ are similar in magnitude. Thus, depending on the trap level and the quasi-Fermi levels, SRH recombination may lead to 1 < *δ* < 2, but in consequence, the ideality factor *n*_id_ will assume non-integer values over a wide range of Fermi levels, that is, 1 < *n*_id_ < 2.

Equation (1) describes SRH recombination in a steady-state situation relevant for explaining the current–voltage curve, the open-circuit voltage and the steady-state PL. For a transient experiment, the SRH formalism becomes a set of coupled rate equations that can be solved numerically (Supplementary Note 6). Analytical solutions are possible^15,16^ but are commonly used only in the absence of detrapping. A perovskite film on glass whose recombination is dominated by a deep trap will exhibit a monoexponential PL decay at sufficiently low excitation conditions, where radiative recombination can be disregarded. In the presence of shallow traps, however, the decay will have additional features related to detrapping and changes in trap occupation due to the movement of the quasi-Fermi levels relative to the trap position during the transient process. Note that increased apparent lifetimes caused by detrapping have previously been reported for multicrystalline Si wafers^21,22^ and for kesterite solar cells^23^.

## PL experiments

**Fig. 2:**
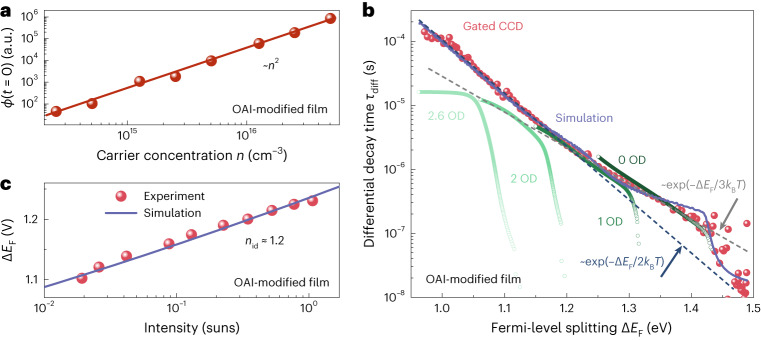
The tr-PL and steady-state PL results of OAI-modified films. **a**, Change of initial amplitude *ϕ*(*t* = 0) of tr-PL decay curve (time-correlated single-photon counting set-up) for an OAI-modified perovskite film as a function of carrier concentration. The amplitude is proportional to *n*^2^, indicating the film is intrinsic. **b**, Measured differential decay time as a function of Fermi-level splitting by both time-correlated single-photon counting (using different optical density (OD) filters) and gated CCD set-ups. Note that the curve for 0 OD is nearly overlapping with the gated CCD curve. The solid line is the simulated result. **c**, Calculated and simulated results of Δ*E*_F_ versus illumination intensity for OAI-modified film. The calculated data are based on the steady-state PL results. Source data

To investigate the nature of defects, we prepared Cs_0.05_FA_0.73_MA_0.22_PbI_2.56_Br_0.44_ triple-cation perovskite films post-treated with *n*-octylammonium iodide (OAI), with a bandgap of ∼1.63 eV, and subsequently fabricated ITO/Me-4PACz/perovskite/C_60_/BCP/Ag inverted solar cells with non-radiative recombination losses as low as ∼100 mV (ITO, indium tin oxide; Me-4PACz, [4-(3,6-dimethyl-9*H*-carbazol-9-yl)butyl]phosphonic acid; BCP, bathocuproine). To quantitatively analyse non-radiative recombination, we measured tr-PL decays as a function of light intensity. Figure 2a shows that the initial PL flux *ϕ*(*t* = 0) of transient PL measurements scales with the square of the laser power and hence with *n*^2^, suggesting that the electron and hole concentrations are identical just after the pulse (that is, before recombination could have happened) over a range of pulse intensities. Thus, the perovskite film cannot have either a high hole or a high electron density in the dark, as otherwise the initial amplitude should have scaled linearly with laser power, as seen for instance for Sn-based perovskites^17^. Thus, we can treat the OAI-modified film as well as an unmodified control (Supplementary Fig. 12) as intrinsic semiconductors—a finding that is consistent with reports on similar triple-cation perovskites^24^.

Furthermore, we measured tr-PL decays with two different methods (single-photon counting and a gated CCD (charge-coupled device) camera) over approximately ten orders of magnitude in dynamic range (Fig. 2b). In Fig. 2b, the normalized decay data have been changed to differential decay time versus Fermi-level splitting. The details of the transformation and the figure before transformation can be found in Supplementary Fig. 4. Figure 2b shows that the tr-PL data obtained using the gated CCD camera features a constantly changing decay time that varies from tens of nanoseconds at high Fermi-level splitting (beginning of the decay) to >100 µs at a Fermi-level splitting of ∼1 V. The covered range of ∼500 meV in Fermi-level splitting corresponds to exp(500 meV/*k*_B_*T*) ≘ 9.5 orders of magnitude dynamic range (*k*_B_, Boltzmann’s constant). Increasing the laser intensity can enhance the signal-to-noise ratio, which can further increase the dynamic range to over ten orders of magnitude. Then a detectable decay time of >280 µs was observed (Supplementary Fig. 7). The decay time (*τ*) is nearly continuously changing with a constant slope, where *τ* ∝ exp(–Δ*E*_F_/(*θk*_B_*T*)), 2 ≤ *θ* ≤ 3 and *E*_F_ is the Fermi energy. This implies that the decay is approximately consistent with a power law of the type *ϕ* ∝ *t*^–2^ as expected (Supplementary Fig. 5) for radiative recombination—or shallow defects—in an intrinsic semiconductor. An alternative to the determination of a (constantly changing) decay time is therefore the determination of a differential recombination coefficient *k*_diff_ (details in Supplementary Note 1), which would be constant for a recombination quadratic in free carrier density. This would enable the description of the recombination dynamics by a single constant parameter in the case of the absence of deep defects. Such a recombination coefficient has been frequently used in the organic solar cell community^25,26^ but is so far uncommon in the description of halide perovskites.

We also note that the single-photon counting data partly overlap with the data from the gated CCD but have additional features. Due to the repetition rate limitation of the single-photon counting method, the decay times shown using the gated CCD are impossible to measure in the absence of a measurement system that can handle repetition rates below a few kilohertz (Supplementary Fig. 4). In addition, we demonstrate that exponential fitting is unable to reliably extract PL decay times (Supplementary Table 1). Finally, we measured the steady-state PL to determine the ideality factor of the films and obtained a non-integer ideality factor of *n*_id_ ≈ 1.2 (Fig. 2c).

To verify that the data are consistent with shallow traps but inconsistent with recombination being limited by deep traps, we use a rate-equation model to simulate both the tr-PL and the steady-state PL data (equations are shown in Supplementary Note 6). The solid lines shown in Fig. 2b,c represent fits to the data, with the parameters shown in Supplementary Table 2. We use three shallow defects to fit the data, and they have a distance to the nearest band of about 55, 95 and 125 meV. Furthermore, we know from Fig. 2a that the defects cannot dope the layer, that is, they have to be acceptor-like defects close to the conduction band or donor-like defects close to the valence band (as visualized in Supplementary Fig. 17). We note that defect positions closer to mid-gap would lead to substantially different shapes of the tr-PL (Supplementary Fig. 20). Furthermore, the only way to consistently explain decay times of hundreds of microseconds in combination with steady-state PL values that are much lower than the radiative limit is to invoke the presence of shallow traps that release charge carriers at longer times, thereby leading to a delayed luminescence, a power-law decay and, in consequence, extremely long decay times towards the end of the decay. Detrapping effects can cause the peculiar situation of PL decay times that increase with increasing shallow defect density, which is the opposite trend as that observed for deep defects (Supplementary Fig. 23).

## Influence of charge-extracting layers

**Fig. 3:**
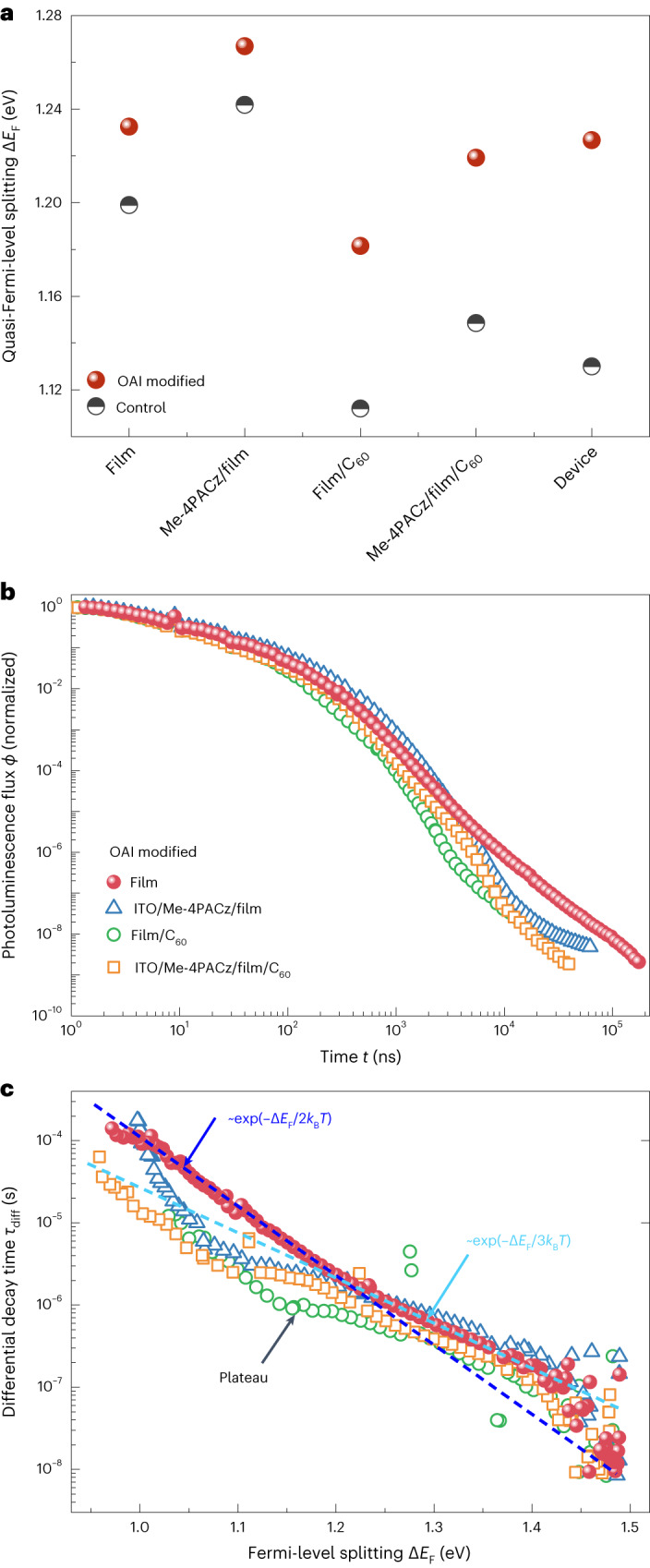
PL characteristics of layer-stack samples. **a**, Calculated quasi-Fermi-level splitting for control and OAI-modified samples with different layer stacks, which were measured under 1 sun equivalent illumination. **b**, The tr-PL decay curves of OAI-modified samples with different layer stacks measured by the gated CCD set-up. **c**, Differential decay time as a function of Fermi-level splitting for OAI-modified samples with different layer stacks measured by the gated CCD set-up. Source data

Figure 3a shows the Δ*E*_F_ of layer-stack samples acquired from steady-state PL measurements (Supplementary Fig. 30). The ITO/Me-4PACz/perovskite sample shows the highest Δ*E*_F_ value. However, interfacing the perovskite layer with C_60_ substantially lowers Δ*E*_F_, possibly by introducing additional interfacial defects, as suggested by studies^27,28^ and consistent with previous reports on steady-state^29^ and transient PL^15,30,31^. OAI modification can effectively passivate the perovskite/C_60_ interface defects, as samples with the perovskite/C_60_ interface show a stronger enhancement in Δ*E*_F_ after OAI modification than stacks without C_60_. Figure 3b,c shows the time-dependent tr-PL decay curves and the differential decay time *τ*_diff_ as a function of the Δ*E*_F_ of different layer stacks. The decay times of different stacks are remarkably similar. While interfaces between perovskite and C_60_ reduce Δ*E*_F_ values, interfaces with only Me-4PACz show an increase in Δ*E*_F_. This indicates that film growth on Me-4PACz improves the bulk properties and suggests that the Me-4PACz/perovskite interface is electronically rather benign. This also contributes to the Me-4PACz/perovskite samples showing the longest *τ*_diff_ at high Δ*E*_F_ values. While the general shape of the *τ*_diff_ versus Δ*E*_F_ curves is similar, the samples with charge-extracting interfaces (either electron transport layer ETL or hole transport layer HTL) show a somewhat lower slope at intermediate values of Δ*E*_F_ (highlighted by the plateau in the figure). Possible reasons for this feature are Coulomb effects that have previously been shown to lead to an S-shaped decay time versus Δ*E*_F_ curves^15,32^.

## Device characteristics

**Fig. 4:**
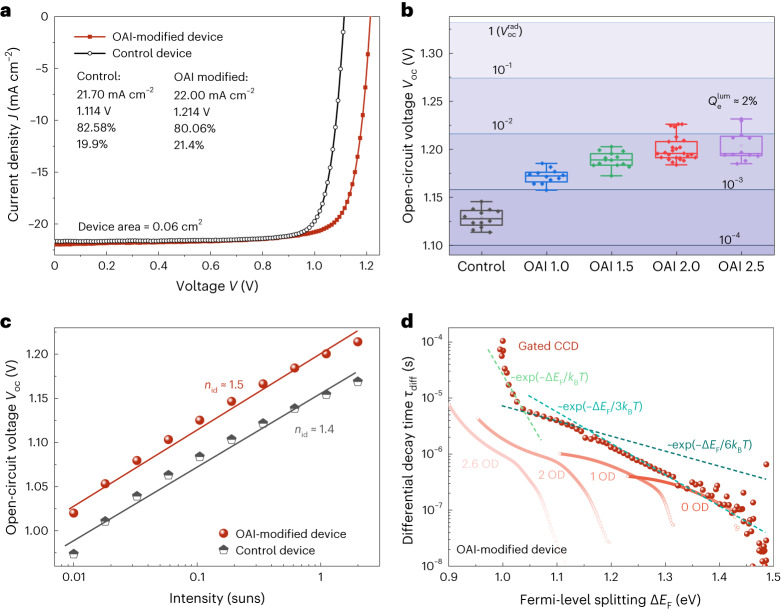
Device performance. **a**, *J*–*V* curves of the control and OAI-modified (2 mg ml^–1^) small-area device. The value shown in the figure, from top to bottom, is *J*_sc_, *V*_oc_, *FF* and efficiency. **b**, Statistical open-circuit voltage data of control devices and OAI-modified devices with different OAI concentrations in milligrams per millilitre. The solid lines indicate the PL quantum yields. The box contains the values from the upper to lower quartiles. The lines outside the box indicate the ×1.5 interquartile range, and the line inside the box is the median. The open square inside the box is the mean value. The sample size for each group, from left to right, is 12, 12, 14, 26 and 12. **c**, Open-circuit voltage of control and OAI device as a function of illumination intensity. **d**, Differential decay time *τ*_diff_ as a function of Δ*E*_F_ for the OAI-modified device. Source data

Finally, inverted solar cells were fabricated based on the films. Figure 4a shows that the *V*_oc_ of the device increases from 1.114 to 1.214 V after OAI modification, resulting in an efficiency increase from 19.9% to 21.4%. Figure 4b presents the statistical distribution of open-circuit voltages as a function of the OAI concentration. The best devices reach a *V*_oc_ over 1.23 V corresponding to a non-radiative recombination loss: $\,{\Delta V}_{{\rm{oc}}}^{\,{\rm{nonrad}}}={V}_{{\rm{oc}}}^{\,{\rm{rad}}}-{V}_{{\rm{oc}}}\approx 100 \ {\mathrm{mV}}$ (current density versus voltage (*J*–*V*) curves in Supplementary Fig. 36), whereby the open-circuit voltage in the radiative limit is given by ${V}_{{\rm{oc}}}^{\,{\rm{rad}}}=1.332 \ {\rm{V}}$. The horizontal lines represent the values of *V*_oc_ expected for different PL quantum efficiency (${Q}_{{\rm{e}}}^{{\rm{lum}}}$) values. Results show that ${Q}_{{\rm{e}}}^{{\rm{lum}}}$ increases from ∼0.04% to ∼2% as a function of OAI concentration. Although the value is lower than the record value of >5% (refs. ^33,34^), it is still higher than most triple-cation-perovskite-based inverted devices (Supplementary Fig. 37). More details about device performances as well as the band alignment between absorber and contact layers can be found in Supplementary Note 4. Figure 4c shows the intensity-dependent open-circuit voltages of full devices, from which we derive ideality factors of around 1.4 (control) to 1.5 (OAI-modified device). These ideality factors are considerably lower than 2, as would be expected from an intrinsic absorber layer dominated by a deep defect, and are therefore consistent with the assumption that the existence of shallow traps still dominates the behaviour in the final cell. This observation is further corroborated by the behaviour of the decay times from tr-PL, shown in Fig. 4d. The decay times show a rather similar behaviour to the films and layer stacks shown in Fig. 3c. At high Δ*E*_F_ (for example, 1.3–1.5 eV), the high carrier concentration results in strong radiative recombination, leading to the fast variation of *τ*_diff_. In the intermediate region (for example, 1.05–1.3 eV), the decay follows a roughly constant slope of approximately exp(–Δ*E*_F_/6*k*_B_*T*), which is less steep than for the films and layer stacks. At low Δ*E*_F_ (for example, <1.05 eV), *τ*_diff_ sharply increases again. One possible reason could be the capacitance effect caused by the electrodes^15^. The single-photon counting data cannot reflect the real variation of *τ*_diff_ in the low Δ*E*_F_ region because of the limitation of the repetition rate.

## Outlook

We show that typical triple-cation perovskite layers, layer stacks and solar cells are strongly affected by shallow defects that manifest themselves in steady-state and transient PL data. Detrapping from such shallow traps then leads to extremely long decay times of hundreds of microseconds that can only be measured using a technique with an extremely low repetition rate. These shallow traps are less problematic for device performance than deeper traps with given SRH lifetimes of *τ*_n_ and *τ*_p_ but are still dominating the steady-state properties. Furthermore, the signatures of shallow traps in transient and steady-state experiments are difficult to distinguish from radiative recombination, which may have contributed to the wide spread of reported values for the radiative recombination coefficient in lead halide perovskites^35–40^ as well as the frequent reports on non-radiative contributions to the quadratic recombination coefficient^9,12,38,39,41^. Furthermore, the work highlights that the often used approximations of the SRH recombination rate must be applied with caution and should not be considered as the default recombination model. The work also shows that the absolute value of the PL decay time extracted from single- or multiexponential fits to low dynamic range fractions of the complete datasets can lead to highly misleading values as decay times may vary over orders of magnitude (tens of nanoseconds to hundreds of microseconds) depending on the excitation density and the repetition rate of the PL set-up. Thus, considering the decay time observed from transient experiments on halide perovskites to be a single number is one of the key fallacies the community needs to overcome to gain insights on recombination dynamics in these materials. A possible alternative to effective decay times for decays that rather resemble a power law instead of an exponential decay is the determination of an effective recombination coefficient.

## Methods

### Materials

Methylammonium iodide, methylammonium chloride, formamidinium iodide and OAI were purchased from Greatcell Solar. Caesium iodide (CsI, 99.9%) was purchased from Alfa Aesar. Lead(II) iodide (PbI_2_, 99.99%), Me-4PACz (>99.0%) and BCP (>99.0%) were purchased from TCI. Lead bromide (PbBr_2_, 99.999%), caesium bromide (CsBr, 99.999%), anisole (99.7%), *N*,*N*-dimethylformamide (DMF, 99.8%), dimethyl sulfoxide (DMSO, ≥99.9%), isopropyl alcohol (IPA, 99.5%), chlorobenzene (99.8%) and poly(methyl methacrylate) (PMMA, weight-averaged molecular mass *M*_w_ ≈ 120,000 by gel permeation chromatography (GPC)) were purchased from Sigma-Aldrich. Ethyl alcohol (EtOH, maximum 0.003% water) and acetone were purchased from VWR Chemicals. C_60_ was purchased from Lumtec. All chemicals were used as received. GaAs (intrinsic, 350 μm thick) wafers were purchased from Suzhou Jingguikeji Company.

### Device fabrication

Patterned ITO glasses (Kinetic, 2.0 × 2.0 cm^2^) were used as substrates and ultrasonically cleaned with soap solution (Seife Hellmanex III, 2%, 50 °C, 20 min), acetone (20 °C, 20 min) and IPA (20 °C, 20 min), one by one. The substrates were further cleaned by oxygen plasma (Diener Zepto, 50 W, 13.56 MHz, 10 min) and then transferred into a N_2_-filled glove box to await use. The Me-4PACz powder was dissolved by EtOH solvent with a concentration of 1 mmol l^–1^. After it was completely dissolved, the solution was spin-coated on the substrates at 3,000 r.p.m. for 25 s (acceleration time, 4 s) and then annealed at 100 °C for 10 min. For the perovskite solution, 1.2 M Cs_0.05_FA_0.73_MA_0.22_PbI_2.56_Br_0.44_ triple-cation perovskite precursor solution was prepared by mixing CsI (0.06 M), methylammonium (MA) iodide (0.264 M), formamidinium (FA) iodide (0.876 M), PbBr_2_ (0.264 M) and PbI_2_ (0.936 M) solutes in DMF/DMSO (3:1 volume ratio) solvent. Then PMMA (∼0.06 mg ml^–1^) was added to the solution. The precursor solution was stirred at 75 °C until fully dissolved, and then filtered with a polytetrafluoroethylene filter (0.45 μm). Some 180 μl solution was dropped onto the Me-4PACz layer and spin-coated at 4,000 r.p.m. for 15 s (acceleration time, 5 s) and 6,000 r.p.m. for 40 s (acceleration time, 5 s). Some 300 μl anisole was dripped onto the film as an antisolvent 20 s before the end of the spin process. The films were immediately annealed at 100 °C for 20 min. For the OAI-modified sample, 100 μl OAI/IPA solutions (with concentrations of 1, 1.5, 2 and 2.5 mg ml^–1^) were each dynamically spin-coated on a perovskite layer at 5,000 r.p.m. for around 30 s, and then annealed at 100 °C for 5 min. The as-prepared films were covered by C_60_ (25 nm) and BCP (8 nm) layers by thermal evaporation at a rate of 0.1 Å s^–1^. Finally, 80 nm of silver was thermally evaporated on the film with a mask. All of the solution preparation and film preparation was performed in a N_2_-filled glove box and attached thermal evaporation system. The active cell area (0.06 and 0.16 cm^2^) is the intersection of the silver and patterned ITO.

### Material characterizations

The surface morphologies of the perovskite films were characterized by scanning electron microscope (Zeiss LEO 1550VP). Absorptance spectra of the film samples were measured by an ultraviolet–visible–near-infrared spectrometer (PerkinElmer Lambda 950). The thickness of perovskite films was measured by a step profiler (Veeco Dektak 6M). Ultraviolet photoelectron spectroscopy (UPS) measurements were carried out for each layer to investigate the energy level alignment. The UPS system is a Multiprobe MXPS system from Scienta Omicron with an ARGUS hemispherical electron spectrometer and is part of the JOSEPH cluster system at the research center in Jülich. The base pressure in the system is 3 × 10^−11^ mbar. The light source for UPS measurement is a HIS13 He I gas discharge VUV source from FOCUS (main line He Iα, 21.22 eV). The binding energy scale is referenced to the Fermi edge measured on a freshly evaporated gold sample, measured under identical conditions. Work functions were determined from the spectra by measuring the position of the cut-off at high binding energies using linear fits at the background and the steep edge. The same method was applied to the leading edge of the UPS spectra to determine the valence band position for the HTL and ETL. For the control and OAI-treated perovskite films, the valence band onset was plotted on a logarithmic scale and was determined by using exponential fitting.

### Device characterizations

The current–voltage curves were measured by a calibrated air mass 1.5 (AM1.5) spectrum of a class AAA solar simulator (WACOM-WXS-140S-Super-L2 with a combined xenon/halogen lamp-based system) using a crystalline silicon cell as a reference and providing a power density of 100 mW cm^–2^. The reference cell was certified by the photovoltaic calibration laboratory at the Fraunhofer ISE, Germany, and the spectral mismatch factor is ∼0.98. For both forward and reverse scans, the scan speed was about 76 mV s^–1^ with a measurement time of around 17 s. All the samples were kept uniformly under the light for 5 s before scanning without any other preconditioning. Additionally, a white light light-emitting diode (LED; Cree XLamp CXA3050) was also used as light source. The light intensity of the LED was adjusted to the one sun condition using the short-circuit current resulting from the solar simulator measurement of a perovskite solar cell. A 2450 Keithley was used as a source measure unit. All measurements were carried out under inert atmosphere in a glove box. We did not use a mask for the measurements, as it would lead to erroneous *V*_oc_ and fill factor FF values with masking, though the determination of short-circuit current density *J*_sc_ can be more accurate^42^. To solve the *J*_sc_ issue, we consider that validating the *J*_sc_ with the external quantum efficiency results is a good choice. For our samples, they matched well with each other.

For the external quantum efficiency measurement, a set-up with a xenon light source (Osram XPO 150 W) and a Bentham monochromator (TMC 300) was used. A photodiode (Gigahertz Optik SSO-PD 100-04) was used to calibrate the light source. The cells were mounted inside a sealed, nitrogen-filled sample box with a quartz cover glass. The raw data of external quantum efficiency and integrated *J*_sc_ have been corrected by subtracting the reflectance of the cover glass.

Fourier-transform photocurrent spectroscopy measurements were carried out using a Fourier-transform infrared spectrometer (Bruker Vertex 80v) equipped with a halogen lamp. A low-noise current amplifier (Femto DLPCA-200) was used to amplify the photocurrent generated upon illumination of the solar cell devices with light modulated by the Fourier-transform infrared spectrometer. We used a mirror speed of 2.5 kHz and a resolution of 12 cm^−1^. Measurements with different filters were combined to get a spectrum with a higher dynamic range of the bandgap.

### Sample preparation for PL measurement

We prepared five types of sample for PL measurement, that is, perovskite film, film/C_60_, Me-4PACz/film, Me-4PACz/film/C_60_ and the full device. Unless otherwise noted, the OAI-modified perovskite samples were prepared using 2 mg ml^–1^ OAI/IPA solution. Perovskite film and film/C_60_ samples were prepared on quartz glass substrates. In order to reduce the defect density of the glass/perovskite interface, we prepared PMMA film on the glass before perovskite preparation. To be specific, 20 mg ml^–1^ PMMA was dissolved in chlorobenzene solvent and then spin-coated on the quartz glass at 3,000 r.p.m. for 25 s, followed by annealing at 100 °C for 10 min. Other types of stacks were prepared on ITO and have the same preparation parameters as devices described previously.

Apart from the triple-cation perovskite, we also performed transient PL measurement for Cs_0.05_FA_0.95_PbI_3_ and CsPbBr_3_ films, as well as GaAs wafer. The GaAs wafer was purchased from a company without any treatment. The CsPbBr_3_ precursor was prepared by adding 0.35 M CsBr and 0.35 M PbBr_2_ into DMSO solution. After fully dissolving it at 70 °C, 100 μl solution was dropped on the bare glass and spin-coated at 4,000 r.p.m. for 1 min, followed by annealing at 100 °C for 10 min. As for Cs_0.05_FA_0.95_PbI_3_, the precursor was mixed together with formamidinium iodide (1.71 M), PbI_2_(1.8 M) and CsI (0.09 M) powders and then dissolved in DMF/DMSO (8:1 v/v). To facilitate crystallinity, we added an extra 5% PbI_2_ and 30% methylammonium chloride (molar ratio) into the solution. The perovskite layer was prepared by spin-coating with ∼100 μl precursor at 1,000 r.p.m. (10 s) and 5,000 r.p.m. (30 s). Some 200 μl chlorobenzene was used as antisolvent and dropped onto the film at 10 seconds prior to the end. The film was annealed at 100 °C for 30 minutes. All processes were performed in the glove box.

### The tr-PL measurement

The tr-PL decay was measured using time-correlated single-photon counting and gated CCD recording, separately. For the time-correlated single-photon counting set-up, a 630 nm laser with a pulse width of 96 ps was used. The laser spot size was 50 μm in diameter, and the laser pulse repetition rate applied was 25 kHz and 50 kHz. The time resolution of the system was approximately 2 ns. To vary the laser intensity hitting the sample, filters with different OD values were used. The applied excitation fluences using 2.6 OD, 2 OD, 1 OD and 0 OD filters were 2.00, 7.97, 79.65 and 796.54 nJ cm^–2^, respectively. The whole system was placed in a black box to protect the signal from ambient light.

Regarding to gated CCD recording, a pulsed UV–solid-state laser was used as an excitation source, which served as a pump laser for the dye laser. The set-up parameters followed the following description unless otherwise noted. The pumped dye (Coumarin) used in the tr-PL set-up emitted a down-converted, pulsed laser radiation of 512 nm. The repetition rate was 100 Hz. This radiation passed through an optical fibre and impinged at an angle of 30° on the sample surface, illuminating an elliptically shaped spot with a diameter 3.07 mm on the samples. The applied excitation fluence was around 2.83 μJ cm^–2^, making the corresponding initial carrier concentration and Δ*E*_F_ values 1.46 × 10^17^ cm^−3^ and 1.48 eV, respectively. The PL signal emitted by the samples was focused and coupled into the spectrometer (SPEX 270M from Horiba Jobin Yvon). An intensified CCD camera (iStar DH720 from Andor Solis) was used to detect the spectrally dispersed signals. To get a time resolution, we exploited the inherent shutter functionality of our intensified CCD camera and a signal of the laser as a trigger. By changing the decay time between the trigger signal and an acquisition of a spectrum, the PL can be measured at different times after the excitation pulse.

For both methods, the samples were mounted inside a sealed, nitrogen-filled sample box. To analyse the data, we first subtracted the background and then normalized the data, as well as shifting the peak to position zero. Detailed instructions can be found in ref. ^15^.

### Steady-state PL measurement and Quasi-Fermi-level splitting calculation

The samples were optically excited by a continuous wave 532 nm laser (Coherent Sapphire). The laser beam was widened to a square of about 5.3 mm × 5.3 mm to illuminate the entire cell area (4 mm × 4 mm). The luminescence spectra were detected via a spectrometer (Andor Shamrock 303) with an Andor Si (deep depletion) CCD camera (iDus Series). The laser power was 17.3 mW. PL measurements were performed for different laser intensities impinging on the sample by using different OD filters. During the measurements, dark spectra were taken following each illuminated measurement to subtract the background.

The quasi-Fermi-level splitting of layer-stack samples was acquired from steady-state PL data. With the open-circuit voltage *V*_oc_ and corresponding PL intensity *ϕ*_PL,cell_ at the 1 sun condition (*V*_oc_(*ϕ*_sun_) and *ϕ*_PL,cell_(*ϕ*_sun_), respectively) of the control device as a reference, the Δ*E*_F_ of layer-stack samples was calculated by

$$\Delta {E}_{{\rm{F}}}=q{V}_{{\rm{oc}}}\left({\phi }_{{\rm{sun}}}\right)+{k}_{{\rm{B}}}T\,{\mathrm{ln}}\left(\frac{{\phi }_{{\rm{PL}}}/\int A{\phi }_{{\rm{BB}}}{{\mathrm{d}}E}}{{\phi }_{{\rm{PL}},{\rm{cell}}}({\phi }_{{\rm{sun}}})/\int {Q}^{{\rm{EQE}}}{\phi }_{{\rm{BB}}}{{\mathrm{d}}E}}\right)$$

where *ϕ*_PL_, *A*, *E* and *Q*^EQE^ are the PL intensity of the sample, absorptance of the films, energy and external quantum efficiency of the cells, respectively. *ϕ*_BB_ is the spectral black-body radiation as shown in

$${\phi }_{{\rm{BB}}}(E\,)=\frac{2{\uppi E}^{2}}{{h}^{3}{c}^{2}}\frac{1}{\exp \left[E/({k}_{{\rm{B}}}T\,)\right]-1}$$

where *k*_B_, *T*, *h* and *c* are Boltzmann’s constant, the temperature, Planck’s constant and light speed in a vacuum, respectively.

### PL quantum yield calculation

The PL quantum yield (${Q}_{{\rm{e}}}^{{\rm{lum}}}$) values of devices were calculated from

$$q\left({V}_{{\rm{oc}}}^{\,{\rm{rad}}}-{V}_{{\rm{oc}}}\right)=-{k}_{{\rm{B}}}T\,{\mathrm{ln}}\left({Q}_{{\rm{e}}}^{{\rm{lum}}}\right)$$

where ${V}_{{\rm{oc}}}^{\,{\rm{rad}}}$ is the radiative open-circuit voltage limit. It can be calculated using the approach described in ref. ^43^.

### Numerical simulation

Band diagrams of devices were simulated using SCAPS software based on the UPS measurement results. The tr-PL and steady-state PL simulations were performed with self-developed MATLAB scripts based on the coupled rate equations (Supplementary Note 6).

### Reporting summary

Further information on research design is available in the Nature Portfolio Reporting Summary linked to this article.

## Online content

Any methods, additional references, Nature Portfolio reporting summaries, source data, extended data, supplementary information, acknowledgements, peer review information; details of author contributions and competing interests; and statements of data and code availability are available at 10.1038/s41563-023-01771-2.

### Supplementary information

Supplementary Information Supplementary Figs. 1–42, Tables 1–3 and Discussion.

Reporting Summary

### Source data

Source Data Fig. 1 Statistical source data.

Source Data Fig. 2 Statistical source data.

Source Data Fig. 3 Statistical source data.

Source Data Fig. 4 Statistical source data.

## Data Availability

The data used in the paper are available at https://zenodo.org/records/10101259. Source data are provided with this paper.

## Code availability

The codes used for the simulation of this study can be accessed at https://zenodo.org/records/10101259.
