# Supplementary material for: Shallow defects and variable photoluminescence decay times up to 280 µs in triple-cation perovskites
Source: Nat Mater. 2024 Jan 9;23(3):391–7. doi: 10.1038/s41563-023-01771-2 (PMC10917677; doi:10.1038/s41563-023-01771-2)
Supplement: Supplementary file 1 — Supplementary Figs. 1–42, Tables 1–3 and Discussion. [file 41563_2023_1771_MOESM1_ESM.pdf]

nature materials

Article

https://doi.org/10.1038/s41563-023-01771-2

Shallow defects and variable photoluminescence decay times up to 280 μs in triple-cation perovskites

In the format provided by the authors and unedited

Table-of-Contents

Supplementary Note 1 Quantifying recombination mechanism behind photoluminescence characterization 2
Supplementary Note 2 Photoluminescence experiments and numerical simulation 9
Supplementary Note 3 Influence of charge extracting layers 35
Supplementary Note 4 Device characteristics 38
Supplementary Note 5 References used for Fig. 1 in the main paper........ 47
Supplementary Note 6 Numerical models 50
References........ 52

Supplementary Note 1 Quantifying recombination mechanism behind photoluminescence characterization

In the following, we will initially derive the basic equations for non-radiative recombination in steady-state situations and then continue with the rate equation model required to determine the transient behavior of a semiconductor film with one or several defect states.

Recombination process via Shockley-Read-Hall recombination via deep defects

Under the assumption that recombination is dominated by Shockley-Read-Hall (SRH) recombination via a defect, the recombination rate is given by[1]

$$R_{\mathrm{SRH}} = N_{\mathrm{t}}\beta_{\mathrm{n}}\beta_{\mathrm{p}}\frac{np-n_{\mathrm{i}}^{2}}{n\beta_{\mathrm{n}}+p\beta_{\mathrm{p}}+e_{\mathrm{n}}+e_{\mathrm{p}}} \tag{1}$$

where N_{t} is the trap density, β_{n}, β_{p} are the capture coefficients of electrons and holes, e_{n}, e_{p} are the emission coefficients of electrons and holes.

The emission coefficients can be expressed as

$$e_{\mathrm{n}} = \beta_{n}N_{\mathrm{c}}\exp\left(\frac{E_{\mathrm{t}}-E_{\mathrm{c}}}{kT}\right) = \beta_{\mathrm{n}}n_{1} \tag{2}$$

$$e_{\mathrm{p}} = \beta_{p}N_{\mathrm{v}}\exp\left(\frac{E_{v}-E_{t}}{kT}\right) = \beta_{\mathrm{p}}p_{1} \tag{3}$$

where N_{c} and N_{v} are the effective density of states for conduction and valence band, E_{t} is the trap energy. $n_{1} = N_{\mathrm{C}}\exp[(E_{\mathrm{t}}-E_{\mathrm{C}})/kT]$ and $p_{1} = N_{\mathrm{V}}\exp[(E_{\mathrm{V}}-E_{\mathrm{t}})/kT]$ are the trap-assisted electron and hole concentration. In case of $n = p$ and $n >> n_{i}$, the SRH recombination rate can be written as[1]

$$R_{\mathrm{SRH}} = \frac{n^{2}}{(n+n_{1})\tau_{\mathrm{p}}+(n+p_{1})\tau_{\mathrm{n}}} \tag{4}$$

where $\tau_{\mathrm{n}} = 1/\beta_{\mathrm{n}}N_{\mathrm{t}}$ and $\tau_{\mathrm{p}} = 1/\beta_{\mathrm{p}}N_{\mathrm{t}}$ are the electron and hole lifetime.

We further assume that the defect is in the middle of the band gap and hence $n >> n_{1} = p_{1}$, as well as assuming $\tau = \tau_{\mathrm{n}} + \tau_{\mathrm{p}}$ (high level injection). Thus, the SRH recombination rate can be simplified as

$$R_{\mathrm{SRH}} = \frac{n}{\tau_{\mathrm{n}}+\tau_{\mathrm{p}}} = \frac{n}{\tau} \tag{5}$$

In this case, if we assume a constant external generation rate G_{ext} and it equals the SRH recombination rate (i.e. $G_{\mathrm{ext}} = R_{\mathrm{SRH}}$), then the Fermi-level splitting can be written as

$$\Delta E_{\mathrm{F}} = k_{\mathrm{B}}T\ln\left(\frac{n^2}{{n_i}^2}\right) = 2k_{\mathrm{B}}T\ln(\frac{G_{\mathrm{ext}}\tau}{n_i}) \quad (6)$$

We define the ideality factor n_{id} as

$$n_{\mathrm{id}} = d\Delta E_F / \left(k_{\mathrm{B}}T \, d\ln(G_{\mathrm{ext}})\right) \quad (7)$$

Substituting Supplementary Eq. (6) into Supplementary Eq. (7), we can have n_{id}=2 under these assumptions.

Recombination process via both radiative recombination and SRH recombination via deep defects

Now, we consider the situation with both radiative recombination and SRH recombination. Thus, the total recombination rate is $R_{\mathrm{tot}} = R_{\mathrm{rad}} + R_{\mathrm{SRH}}$, where the radiative recombination rate is given by $R_{\mathrm{rad}} = k_{\mathrm{rad}}np$. Under the assumption of n=p>> n_1 = p_1 and $n >> n_i$, the total recombination rate can be expressed as

$$R_{\mathrm{tot}} = k_{\mathrm{rad}}n^2 + \frac{n}{\tau_{\mathrm{p}}+\tau_{\mathrm{n}}} \quad (8)$$

Solving for carrier concentration n gives

$$n = \sqrt{\frac{4k_{\mathrm{rad}}R_{\mathrm{tot}}(\tau_{\mathrm{p}}+\tau_{\mathrm{n}})^2+1}{\left[2k_{\mathrm{rad}}(\tau_{\mathrm{p}}+\tau_{\mathrm{n}})\right]^2}} - \frac{1}{2k_{\mathrm{rad}}(\tau_{\mathrm{p}}+\tau_{\mathrm{n}})} \quad (9)$$

Assuming that $G_{ext} = R_{tot}$, then the Fermi-level splitting can be given by

$$\Delta E_{\mathrm{F}} = k_{\mathrm{B}}T\ln\left(\frac{n^2}{{n_i}^2}\right) = 2k_{\mathrm{B}}T\ln\left(\frac{\sqrt{4G_{\mathrm{ext}}k_{\mathrm{rad}}(\tau_{\mathrm{p}}+\tau_{\mathrm{n}})^2+1}-1}{2k_{\mathrm{rad}}n_i(\tau_{\mathrm{p}}+\tau_{\mathrm{n}})}\right) \quad (10)$$

Substituting Supplementary Eq. (10) into Supplementary Eq. (7), we can get that the ideality factor n_{id} approaches 1, in case of $\tau_{\mathrm{p}} + \tau_{\mathrm{n}}$ becomes sufficiently large (namely, the radiative recombination is dominant).

The influence of shallow defect on SRH recombination

In the situation that a shallow defect is close to the conduction band and SRH recombination is dominant, i.e. n_1 >> p_1 and n >> p_1. $n_1 = N_{\mathrm{C}}\exp[(E_{\mathrm{t}} - E_{\mathrm{C}})/k_{\mathrm{B}}T]$ can't be ignored directly. The recombination rate Supplementary Eq. (4) can be simplified to

$$R_{\mathrm{SRH}} = \frac{n^2}{n(\tau_{\mathrm{p}}+\tau_{\mathrm{n}})+n_1\tau_{\mathrm{p}}} \quad (11)$$

Solving for n gives

$$n = \sqrt{\frac{4n_1 R_{\mathrm{SRH}}\tau_{\mathrm{p}} + [R_{\mathrm{SRH}}(\tau_{\mathrm{p}}+\tau_{\mathrm{n}})]^2}{4}} + \frac{R_{\mathrm{SRH}}(\tau_{\mathrm{p}}+\tau_{\mathrm{n}})}{2} \tag{12}$$

Assuming that $G_{\mathrm{ext}} = R_{\mathrm{SRH}}$, in this case, the Fermi-level splitting can be expressed as

$$\Delta E_{\mathrm{F}} = k_{\mathrm{B}}T\ln\left(\frac{n^2}{{n_i}^2}\right) = 2k_{\mathrm{B}}T\ln\left(\frac{\sqrt{4n_1 G_{\mathrm{ext}}\tau_{\mathrm{p}} + [G_{\mathrm{ext}}(\tau_{\mathrm{p}}+\tau_{\mathrm{n}})]^2} + G_{\mathrm{ext}}(\tau_{\mathrm{p}}+\tau_{\mathrm{n}})}{2n_i}\right) \tag{13}$$

When $n_1 \ll G_{\mathrm{ext}}\tau$(where $\tau = \tau_{\mathrm{n}} + \tau_{\mathrm{p}}$), we can simplify the Fermi-level splitting to

$$\Delta E_{\mathrm{F}} = 2k_{\mathrm{B}}T\ln\frac{G_{\mathrm{ext}}\tau}{n_i} \tag{14}$$

Substituting it into Supplementary Eq. (7), we can determine the ideality factor to $n_{\mathrm{id}} = 2$.

On the contrary, when $n_1 \gg G_{\mathrm{ext}}\tau$, the Fermi-level splitting goes to

$$\Delta E_{\mathrm{F}} = 2k_{\mathrm{B}}T\ln\frac{\sqrt{n_1 G_{\mathrm{ext}}\tau_{\mathrm{p}}}}{n_i} \tag{15}$$

And hence the ideality factor approaches $n_{\mathrm{id}} = 1$.

Recombination process via radiative recombination and SRH recombination (shallow and deep defects)

In this situation, the total recombination rate can be expressed as

$$R_{\mathrm{tot}} = k_{\mathrm{rad}}n^2 + \frac{n^2}{n(\tau_{\mathrm{p,shallow}}+\tau_{\mathrm{n,shallow}}) + n_1\tau_{\mathrm{p,shallow}}} + \frac{n}{\tau_{\mathrm{p,deep}}+\tau_{\mathrm{n,deep}}} \tag{16}$$

For an intrinsic semiconductor (such as most Pb-based perovskites), $\tau_{\mathrm{p}} = \tau_{\mathrm{n}}$ and $\tau = \tau_{\mathrm{p}} + \tau_{\mathrm{n}}$ in both shallow and deep defect cases. Hence, Supplementary Eq. (16) can be simplified as

$$R_{\mathrm{tot}} = k_{\mathrm{rad}}n^2 + \frac{n^2}{(n+n_1/2)\tau_{\mathrm{shallow}}} + \frac{n}{\tau_{\mathrm{deep}}} \tag{17}$$

In the case of $n << n_1$ (such as a situation that a perovskite has shallow defect and low excess carrier concentration),

$$R_{\mathrm{tot}} = (k_{\mathrm{rad}} + 2/n_1\tau_{\mathrm{shallow}})n^2 + \frac{n}{\tau_{\mathrm{deep}}} \tag{18}$$

Here we can see that a sufficiently shallow defect in SRH can behave like radiative recombination (so second order in excess carrier density).

In our case, the OAI modified perovskite films didn't show any obvious deep defect. And if we assume

$k_{\mathrm{tot}} = k_{\mathrm{rad}} + 2/n_1\tau_{\mathrm{shallow}}$, the equation can be further simplified to

$$R_{\mathrm{tot}} = k_{\mathrm{tot}} n^2 \qquad (19)$$

Additionally, for a general case, to evaluate the recombination coefficient of experimental data measured at steady-state condition, we define an effective recombination coefficient k_{eff} as

$$k_{\mathrm{eff}} = \frac{R}{n^2} = \frac{G}{n^2} = \frac{G}{n_{\mathrm{i}}{}^2 \exp{(\Delta E_{\mathrm{F}}/k_{\mathrm{B}}T)}} \qquad (20)$$

Differing from Supplementary Eq. (19), the k_{eff} contains the effects of radiative recombination and SRH recombination through both shallow and deep defects (see Supplementary Fig. 14).

SRH recombination via shallow defect during transients

While the steady-state SRH recombination rate according to Supplementary Eq. (1) is frequently used in the community, it is only applicable to steady-state situations. In particular, the derivation assumes that the occupation of the trap is a constant given by the SRH occupation statistics. In transient experiments, however, not only the concentration of free electrons and holes changes as a function of time after a laser pulse, but also the occupation of the trap levels changes. Initially, it is typically empty and trap filling will occur at earlier times. At later times, detrapping will become relevant. As observed in ref.[2] , transient PL therefore probes a different situation than steady state PL and in consequence also the equations needed to explain the decay are different to the steady-state solutions for the SRH recombination rate. The recombination dynamics in the presence of a recombination path via only one active shallow trap reads

$$\frac{dn}{dt} = \frac{-n(N_{\mathrm{t}} - n_{\mathrm{t}}) + n_1 n_{\mathrm{t}}}{\tau_{\mathrm{n}} N_{\mathrm{t}}} - k_{\mathrm{rad}} np \qquad (21)$$

for the concentration n of free electrons, where the term $\frac{-n(N_{\mathrm{t}} - n_{\mathrm{t}})}{\tau_{\mathrm{n}} N_{\mathrm{t}}}$ and $\frac{n_1 n_{\mathrm{t}}}{\tau_{\mathrm{n}} N_{\mathrm{t}}}$ describes the trapping and detrapping process respectively, as well as the $-k_{\mathrm{rad}} np$ for the radiative recombination process. Additionally, it also has

$$\frac{dn_{\mathrm{t}}}{dt} = \frac{n(N_t - n_{\mathrm{t}}) - n_1 n_{\mathrm{t}}}{\tau_{\mathrm{n}} N_{\mathrm{t}}} + \frac{p_1(N_{\mathrm{t}} - n_{\mathrm{t}}) - p n_{\mathrm{t}}}{\tau_{\mathrm{p}} N_{\mathrm{t}}} \qquad (22)$$

for the concentration n_{t} of trapped electrons. We now assume that the change in free electron concentration is faster than the interaction of the trapped electrons with the holes (i.e. $n(N_t - n_{\mathrm{t}}) - n_1 n_{\mathrm{t}} = 0$). If we also

assume that the trap is almost empty, i.e., $n_\mathrm{t} \ll N_\mathrm{t}$, the concentration n of free electron follows closely the concentration n_t of trapped electrons via

$$n \approx \frac{n_1}{N_\mathrm{t}} n_\mathrm{t} \tag{23}$$

By eliminating capture and emission of electrons as well as emissions of holes ($p_1 \ll p$) from Supplementary Eq. (22), we arrive at

$$\frac{dp}{dt} \approx \frac{dn_\mathrm{t}}{dt} \approx -\frac{pn_\mathrm{t}}{\tau_\mathrm{p} N_\mathrm{t}} - k_\mathrm{rad} np \approx -\frac{{n_\mathrm{t}}^2}{\tau_\mathrm{p} N_\mathrm{t}} - \frac{k_\mathrm{rad} n_1 p n_\mathrm{t}}{N_\mathrm{t}} \tag{24}$$

where we further assumed that $n \ll N_\mathrm{t}$ and hence $p = n_\mathrm{t} + n \approx n_\mathrm{t}$. The first term on the right-hand side of Supplementary Eq. (24) describes a quadratic decay of the concentration n_t of trapped electrons dominating the recombination process as the concentrations n and p of free carriers are proportional to n_t. The second term describes radiative recombination of free electrons and free holes, which still affects the concentration of trapped electrons via the fast exchange of free and trapped electrons that we assume here. We can now calculate the differential PL decay time according to

$$\frac{1}{\tau_\mathrm{diff}} = -\frac{1}{2}\frac{\frac{d\phi}{dt}}{\phi} = -\frac{1}{2}\frac{\frac{dn}{dt}p + n\frac{dp}{dt}}{np} = -\frac{1}{2}\left(\frac{\frac{dn}{dt}}{n} + \frac{\frac{dp}{dt}}{p}\right) = \frac{1+k_{rad}\tau_p N_t}{\tau_\mathrm{p} N_\mathrm{t}} n_t. \tag{25}$$

Rewriting the definition of quasi-Fermi level splitting ΔE_F yields

$$n_i^2 \exp\left(\frac{\Delta E_\mathrm{F}}{k_\mathrm{B} T}\right) = np = \frac{n_1}{N_\mathrm{t}} {n_\mathrm{t}}^2 \tag{26}$$

Combining Supplementary Eq. (25) and (26) yields an analytic expression for the dependence of the PL decay time

$$\tau_\mathrm{diff} \approx \tau_p \sqrt{\frac{n_1 N_T}{np}} \frac{1}{1+k_\mathrm{rad}\tau_\mathrm{p} n_1} = \tau_\mathrm{p} \frac{\sqrt{n_1 N_T}}{n_\mathrm{i}} \frac{1}{1+k_\mathrm{rad}\tau_\mathrm{p} n_1} \exp\left(-\frac{\Delta E_F}{2kT}\right) \tag{27}$$

Note that Supplementary Eq. (27) predicts a continuously changing decay time with quasi-Fermi level splitting ΔE_F, and such absolute value increases with the density N_t and shallower energy level (the shallower the defect, the higher n_1).

Generally, differential equations of the type $dn/dt = -kn^2$ lead to solutions of the type $n(t) = n(0)/(1 + n(0)kt)$, which becomes a power law for $n(0)kt \gg 1$. To evaluate the carrier recombination dynamics, we define a differential recombination coefficient k_diff as

$$k_\mathrm{diff} = -\frac{1}{\sqrt{np}} \cdot \frac{d\ln\phi}{dt} \tag{28}$$

Such k_{diff} contains the combined effects of radiative recombination and SRH recombination through defect (including trapping and detrapping related effects). By substituting Supplementary Eq. (26) and Supplementary Eq. (27), we can obtain

$$k_{\text{diff}} \approx \frac{1+k_{\text{rad}}\tau_{\text{p}}n_1}{\tau_{\text{p}}\sqrt{n_1 N_{\text{T}}}} \quad (29)$$

Noted that the k_{diff} is independent of ΔE_{F} in the case of radiative recombination and SRH recombination via shallow defects (see Supplementary Fig. 13 and 14).

Quantifying relationship between Q_e^{lum}, V_{oc} and carrier lifetime τ for deep defects

Steady state photoluminescence (ss-PL) is widely used way to external quantify photoluminescence quantum yields $Q_e^{\text{lum}}$of the film as shown in Method section. Q_e^{lum} is defined as

$$Q_e^{\text{lum}} = \frac{Y_{\text{em}}}{Y_{\text{rec}}} \quad (30)$$

where $Y_{\text{em}}$is the emitted photon flux caused by radiative recombination, and $Y_{\text{rec}}$is the recombination flux, including the effect of radiative recombination and non-radiative recombination (i.e. SRH recombination) (neglecting Auger recombination). Hence, we obtain

$$Q_e^{\text{lum}} = \frac{p_{\text{e}}R_{\text{rad}}}{R_{\text{SRH}}+(1-p_{\text{r}})R_{\text{rad}}} \quad (31)$$

Please note that only part of photons created in the radiative recombination can be emitted out of the film. Another part of them will be reabsorbed by the perovskite film itself. The coefficients of these two parts are named emission probability p_{e} and reabsorption probability p_{r}. If we neglect the influence parasitic absorption in the reabsorption process, $p_{\text{e}} = 1 - p_{\text{r}}$ holds.

Most perovskites are intrinsic and the photoexcited carrier concentration Δn is much higher than the equilibrium carrier concentration n_0 (high level injection). Thus, the carrier lifetime $\tau = \tau_{\text{p}} + \tau_{\text{n}}$. Further assuming that only a deep defect exists in the film (which is a general assumption in the community), we obtain $R_{\text{SRH}} = \Delta n/\tau = n/\tau$ and $R_{\text{rad}} = k_{\text{rad}}n^2$.

Substituting them into Supplementary Eq. (31)

$$Q_e^{\text{lum}} = \frac{p_{\text{e}}k_{\text{rad}}n^2}{\frac{n}{\tau}+(1-p_{\text{r}})k_{\text{rad}}n^2} \quad (32)$$

This equation generally shows the relationship between Q_e^{lum} and carrier lifetime τ. However, the charge

carrier concentration n depends on τ as well. In the following, we aim to derive an equation for n. In the absence of charge extraction, the external generation rate equals the total recombination rate

$$G_{\mathrm{ext}} = R_{\mathrm{tot}} = k_{\mathrm{rad}}(1 - p_{\mathrm{r}})n^2 + \frac{n}{\tau} \tag{33}$$

Solving for n yields

$$n = \frac{-1+\tau\sqrt{-4G_{\mathrm{ext}}k_{\mathrm{rad}}(-1+p_{\mathrm{r}})+\frac{1}{\tau^2}}}{2k_{\mathrm{rad}}(-1+p_{\mathrm{r}})\tau} \tag{34}$$

Substituting Supplementary Eq. (34) into Supplementary Eq. (32), the Q_e^{lum} reads

$$Q_{\mathrm{e}}^{\mathrm{lum}} = \frac{2p_{\mathrm{e}}k_{\mathrm{rad}}G_{\mathrm{ext}}\tau^2}{1+2p_{\mathrm{e}}k_{\mathrm{rad}}G_{\mathrm{ext}}\tau^2+\tau\sqrt{4G_{\mathrm{ext}}k_{\mathrm{rad}}p_{\mathrm{e}}+\frac{1}{\tau^2}}} \tag{35}$$

This equation shows the quantifying relationship between Q_e^{lum} and carrier lifetime τ, where Q_e^{lum} can be quantified by the ss-PL measurement and τ can be quantified by the transient PL measurement.

In real situations, the variation of Q_e^{lum} is always dominated by non-radiative recombination processes caused by deep defects. Lower Q_e^{lum} means stronger non-radiative recombination and lower open-circuit voltage of a solar cell. The quantifying relationship between V_{oc} and Q_e^{lum} can be written as[3],

$$V_{\mathrm{oc}} = V_{\mathrm{oc}}^{\mathrm{rad}} + \frac{k_{\mathrm{B}}T}{q}\ln\left(Q_e^{\mathrm{lum}}\right) \tag{36}$$

Therefore, we can get the quantifying relationship between open-circuit voltage and carrier lifetime τ. The reference lines in Fig.1 are calculated based on Supplementary Eq. (35) and Supplementary Eq. (36).

Nature of the Shallow Defects

Based on the experimental evidence presented in this paper alone, it is not possible to make a very strong statement about the chemical or structural origin of the proposed shallow defects. Of course, they could be intrinsic point defects that happen to be either close to the conduction or close to the valence band and are not charged in thermal equilibrium (zero volt in the dark). In principle, the origin of the shallow traps could also be a polaron that might behave similarly to a shallow defect. However, one would expect that this would involve radiative transitions that are visible as Stokes-shifted luminescence as described in ref.[4]. Given the very steep absorption onset on the small energy difference between absorption onset and photo- or electroluminescence peaks in lead-halide perovskites, this seems unlikely to be able the current observations.

Supplementary Note 2 Photoluminescence experiments and numerical simulation

Photoluminescence experiments were performed on homogenous, pinhole-free samples without impurities obvious in (large) SEM image, as shown in Supplementary Fig. 1. The PL image in Supplementary Fig. 2 shows that the whole film has a very homogenous PL signal intensity distribution in a large area, which also indicates that strong spatial variations of electronic properties cannot cause the strong carrier density dependent of the recombination lifetime observed in the PL decays. Additionally, both control and OAI modified films generally show a similar morphology including grain size and homogeneity. However, the grain boundaries of the OAI modified films become blurrier as shown in Supplementary Fig. 3.

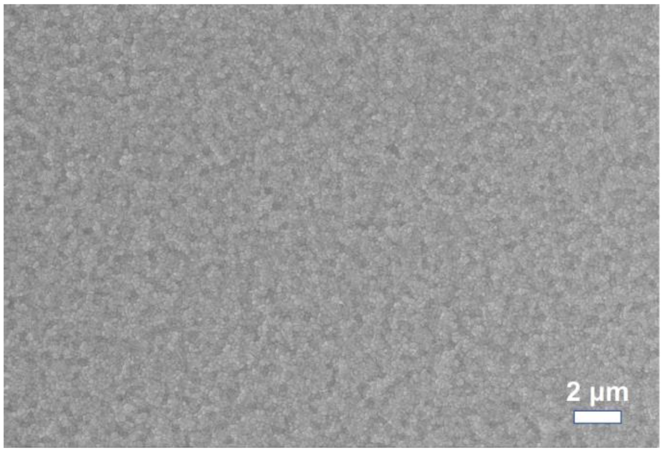

Supplementary Fig. 1 SEM image of OAI modified perovskite film prepared on ITO/Me-4PACz substrate.

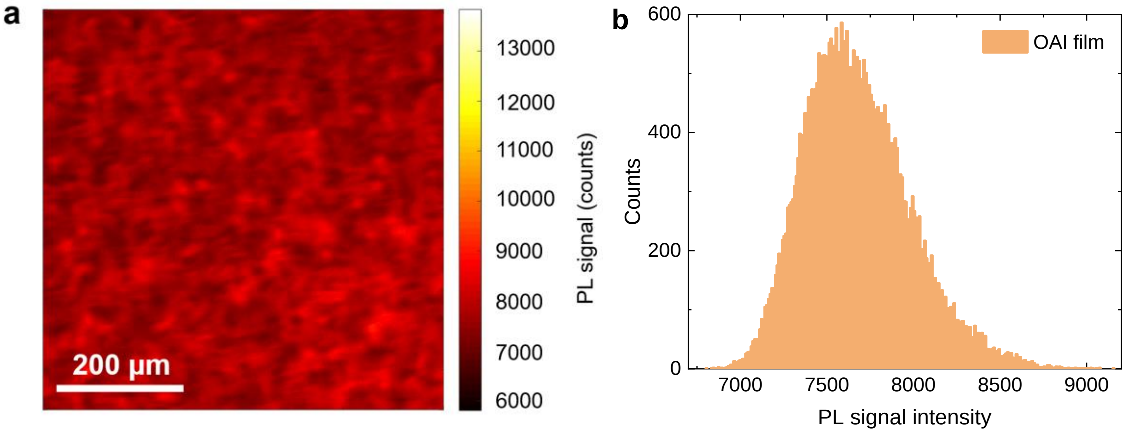

Supplementary Fig. 2 **a** PL mapping image of OAI modified perovskite films and **b** the corresponding PL signal intensity distribution.

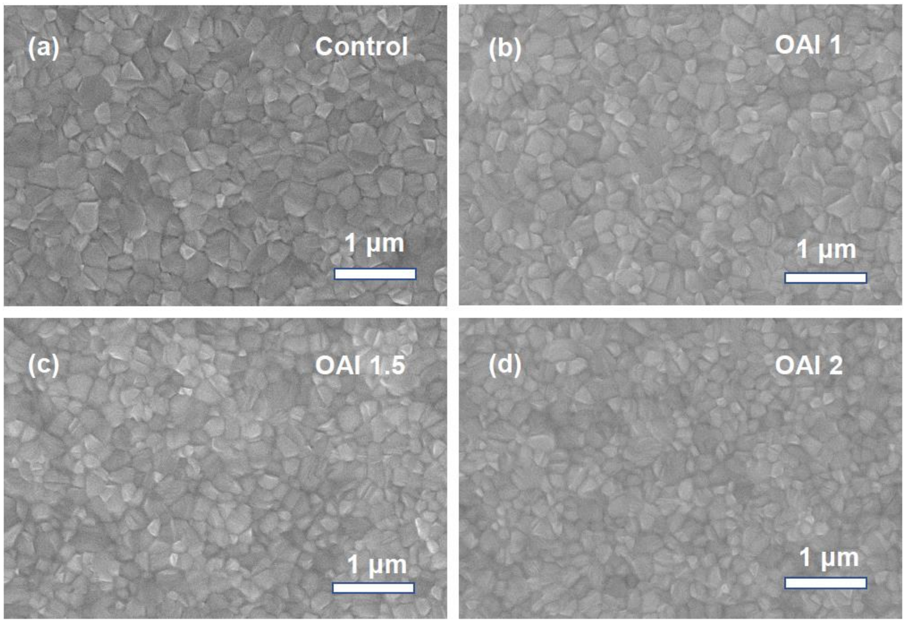

Supplementary Fig. 3 SEM images of **a** control film and **b, c, d** OAI modified films with different OAI concentrations.

Supplementary Fig. 4a shows the original Tr-PL decay curves of OAI modified perovskite film. Obviously, the gated CCD results show a much larger dynamic range, which allows us to obtain a more complete decay process. The shown decay is approximately consistent with a power law rather than a exponent (Supplementary Fig. 5). For the TCSPC setup measurement, we split it into 4 parts by using different laser intensity (by using optical density (OD) filters) to increase the dynamic range. This indeed has increased the Fermi-level splitting range as shown in Supplementary Fig. 4b. But as the measurement is based on single photon counting, the maximum differential decay time is limited by the repetition rate of the equipment. Supplementary Fig. 6 shows the decay curves and corresponding differential decay times τ_{diff} vs. ΔE_{F} using different repetition rates. In order to show the results in the low ΔE_{F} region, we use a 2 OD filter to decrease the light intensity. Obviously, the repetition rate strongly affects the upper limit of the differential decay time τ_{diff} as well as the shape of the curve. In our case, we used a repetition rate of 25 kHz, so the maximum decay time can't exceed ~20 μs (shown as the dashed line in Supplementary Fig. 4b). Thus, we conclude that TCSPC results can't accurately reflect the variation of the curve in the low Fermi-level splitting region.

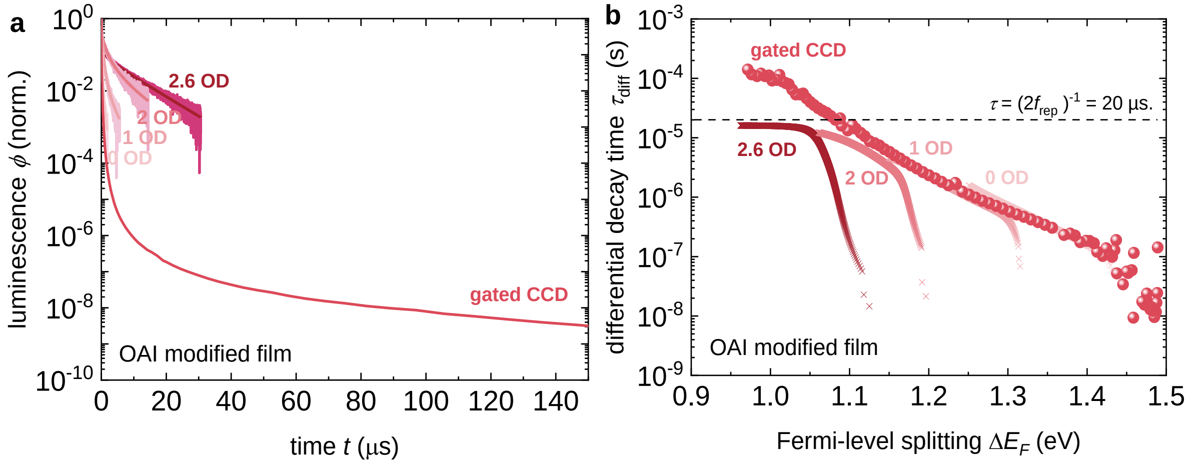

Supplementary Fig. 4 **a** Tr-PL decay curves of OAI modified film measured by gated CCD and TCSPC setups. **b** The corresponding differential decay time τ_{diff} versus Fermi-level splitting ΔE_{F}. For the transformation, we use $\tau_{\mathrm{diff}} = -2(\mathrm{d}\ln(\phi)/\mathrm{d}t)^{-1}$ to calculate the decay time and $\Delta E_{\mathrm{F}}(t) = \Delta E_{\mathrm{F}}(0) + k_{\mathrm{B}}T\ln(\phi(t)/\phi(0))$ to calculate the Fermi-level splitting axis. The initial quasi-Fermi level after the pulse, i.e. at time $t = 0$, was determined by calculating the density of absorbed photons, the average density Δn of generated electrons and subsequently the Fermi level splitting using $\Delta E_{\mathrm{F}}(0) = k_{\mathrm{B}}T\ln\left(\Delta n^2(0)/n_{\mathrm{i}}^2\right)$. Here, $k_{\mathrm{B}}T$ is thermal energy. Additionally, for the TCSPC data, the saturation effects at low ΔE_{F} are related to the minimum repetition rate available in our setup. At a repetition rate of f_{rep} = 25 kHz, the maximum decay time does not exceed a value of approximately $\tau < \left(2f_{\mathrm{rep}}\right)^{-1} = 20$ μs (as the dash line shown).

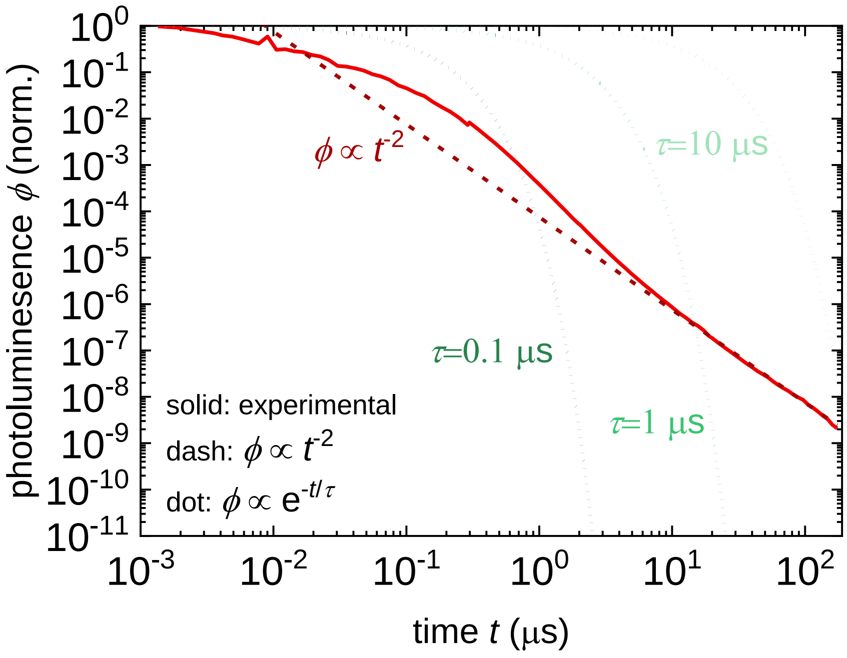

Supplementary Fig. 5 Tr-PL decay curves of OAI modified film measured by gated CCD setup and plotted with double logarithm scale. Reference lines $\phi \propto t^{-2}$ and $\phi \propto e^{-t/\tau}$ were also plotted for comparison. It demonstrates that decay is approximately consistent with a power law.

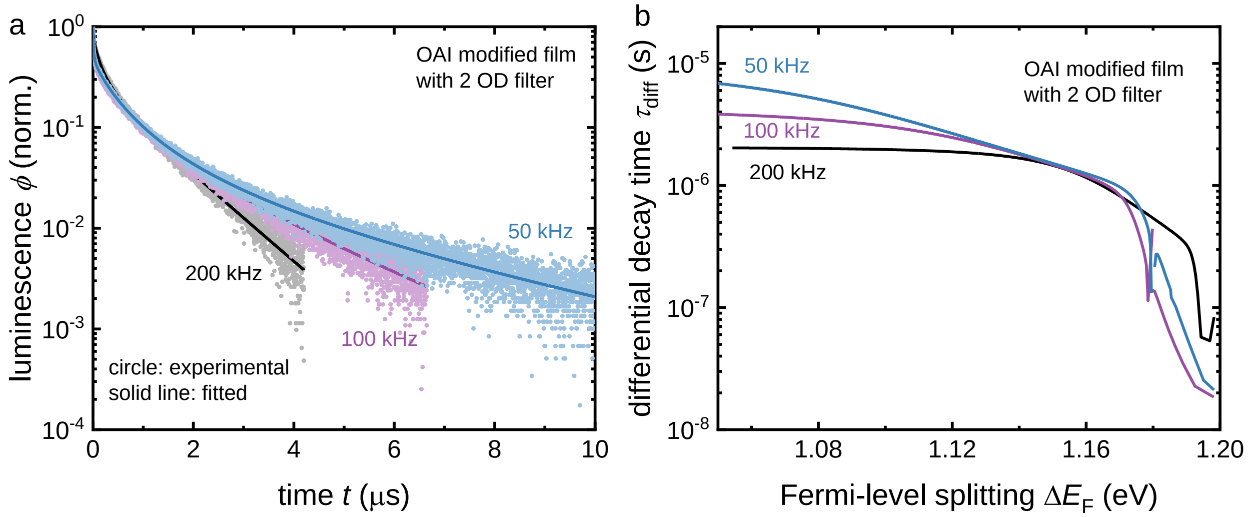

Supplementary Fig. 6 a Tr-PL decay curve and **b** corresponding differential decay time τ_{diff} versus Fermi-level splitting ΔE_F of OAI modified film using TCSPC setup with different repetition rates.

In addition, the widely used exponential fitting is also not a solid way to extract PL decay times.

Supplementary Table 1 shows the bi-exponential fitting results for data measured from the TCSPC setup with different OD filters. The values of τ_2 obviously increase with decreasing laser intensity even for the same sample. It is expected that this value will continuously increase until the detection limit of the setup is reached.

Supplementary Table 1. Comparison of PL decay time of same sample with TCSPC setups using bi-exponential fitting. The applied excitation fluences using 0 OD, 1 OD, 2 OD and 2.6 OD filters are 796.54, 79.65, 7.97 and 2.00 nJ/cm^2, respectively.

Methods	PL decay time (ns)
Bi-exponential fitting with TCSPC setup (0 OD)	τ_1 = 29 τ_2 = 130
Bi-exponential fitting with TCSPC setup (1 OD)	τ_1 =102 τ_2 = 472
Bi-exponential fitting with TCSPC setup (2 OD)	τ_1 =108 τ_2 = 1778
Bi-exponential fitting with TCSPC setup (2.6 OD)	τ_1 =82 τ_2 = 4673

The gated CCD result in Supplementary Fig. 4a shows an ultralong PL decay process, but the decay process has not finished yet. The signal noise ratio (SNR) limits further detection. Thus, we attempted to obtain a better SNR by increasing the laser intensity. The result is shown in Supplementary Fig. 7a and b. The curve shows a dynamic range over 10 orders of magnitude and a surprising PL decay time over 280 µs. Shallow defects should be responsible for this long decay time as discussed in main paper.

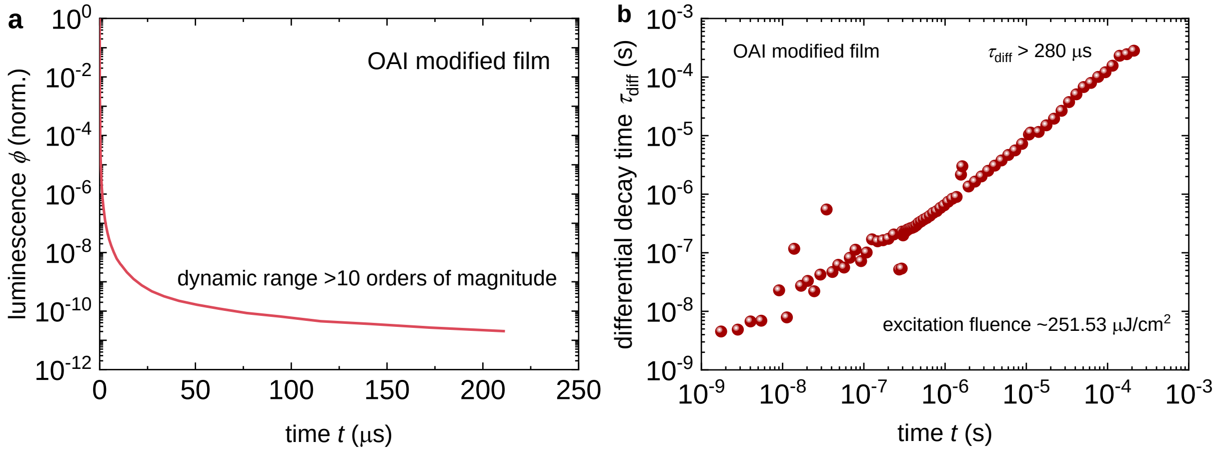

Supplementary Fig. 7 a Tr-PL decay curve and **b** corresponding differential decay time τ_{diff} versus time t of OAI modified film measured by gated CCD setup with higher illumination intensity.

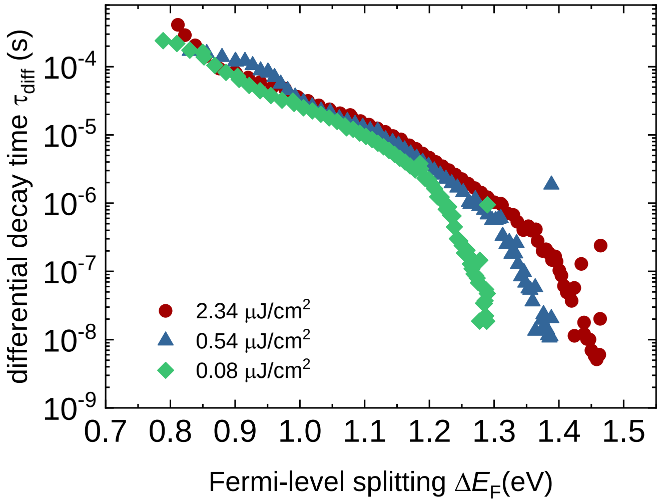

Supplementary Fig. 8 Differential decay time τ_{diff} versus Fermi-level splitting ΔE_F of OAI modified film measured by gated CCD setup using UV laser (343 nm) with different illumination intensity.

As gated CCD setup can measure a large dynamic range, it is not necessary to use different light intensities for the measurement, as the TCSPC setup did. Here, to demonstrate it, we have measured the transient photoluminescence of another OAI modified film sample with different excitation fluences using gated CCD setup, as shown in Supplementary Fig. 8. We can observe that the curves nearly overlap at lower Fermi-level splitting ΔE_F.

Supplementary Fig. 9a shows a comparison of a tr-PL decay of a control and an OAI modified perovskite film under the same measuring condition. The control film shows a much faster decay process than the OAI modified film. As shown in Supplementary Fig. 9b, the control film exhibits an obviously lower differential decay time in the region of 1.1 - 1.4 eV. In this region, nonradiative recombination dominates the decay process. Stronger nonradiative recombination in the control film makes it show lower Fermi-level splitting at the same decay time. In the region below 1.1 eV, the differential decay time of the control film also shows fast increase. It can be seen that this film also has very long maximum PL decay time, just like the OAI modified film.

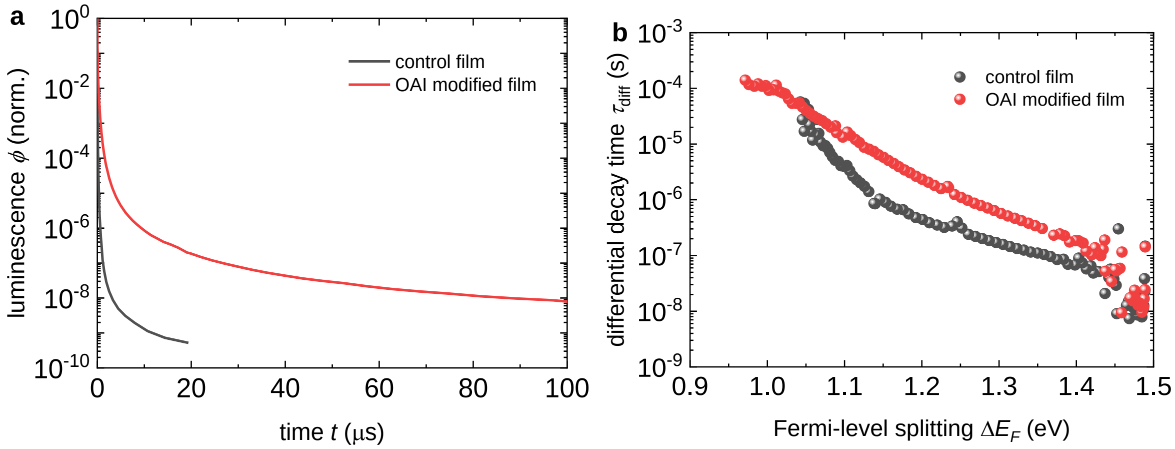

Supplementary Fig. 9 Comparison of **a** tr-PL decay curves and **b** corresponding differential decay time τ_{diff} versus Fermi-level splitting ΔE_F of control and OAI modified films.

For further investigation, we have performed transient PL measurement using gated CCD setup for different Pb-based perovskites with different bandgaps. Here we discuss the universality of power-law type of the decay in the Pb-based halide perovskites. Supplementary Fig. 10 shows the transient PL results of $Cs_{0.05}FA_{0.95}PbI_3$, $MAPbI_3$($MA=CH_3NH_3$), $CsPbBr_3$ films, respectively. They all have power-law type of decay and continuously rising τ_{diff} at low ΔE_F region, which is the same as the control and OAI modified triple cation ($Cs_{0.05}FA_{0.73}MA_{0.22}PbI(I_{0.78}Br_{0.22})_2$) film. Such phenomena indicate that the dominant role of shallow defects is prevalent in pure Pb-based perovskites.

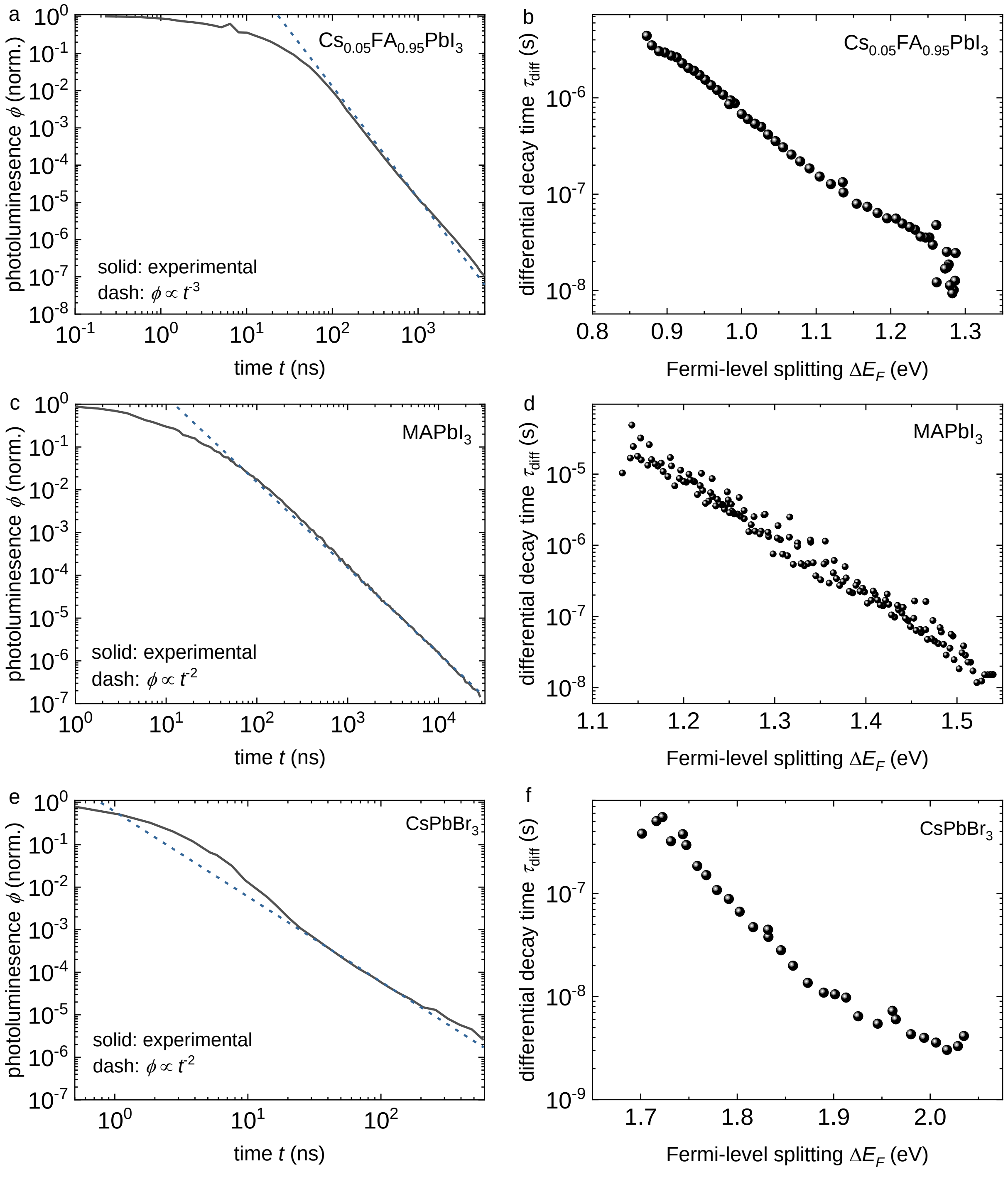

Supplementary Fig. 10 Normalized photoluminescence decay curve and differential decay time τ_{diff} versus Fermi-level splitting ΔE_{F} curve of **a,b** $Cs_{0.05}FA_{0.95}PbI_3$ film; **c,d** $MAPbI_3$ film and **e,f** $CsPbBr_3$ film. The experimental data of $MAPbI_3$ film were redrawn from ref.[5]. Copyright 2021, The Authors, published by Wiley-VCH GmbH.

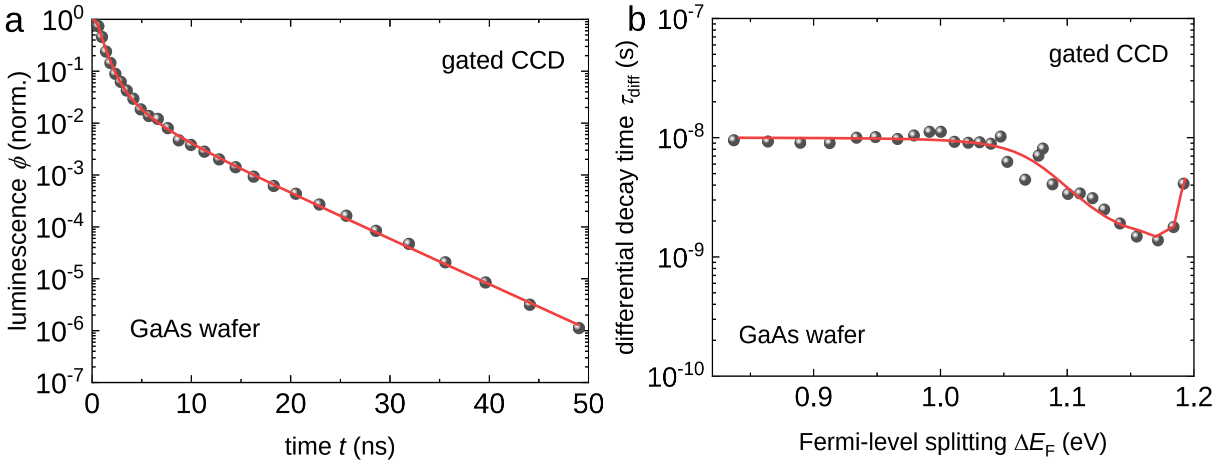

Supplementary Fig. 11 a Tr-PL decay curve and b corresponding differential decay time τ_{diff} versus Fermi-level splitting ΔE_{F} of an intrinsic GaAs wafer measured by gated CCD setup. The resistivity and mobility of GaAs wafer is ~10^8 Ω·cm and ~5300 $\mathrm{cm^2/Vs}$, respectively.

In order to make sure that the ultralong PL decay is indeed the property of perovskite itself and is not caused by artefacts of contamination or component of setup, we have performed transient measurement for GaAs wafer using the same gated CCD setup. Supplementary Fig. 11 shows the normalized PL decay curve and the corresponding differential decay time τ_{diff} versus Fermi-level splitting ΔE_{F} curve of an intrinsic GaAs wafer, suggesting that the GaAs sample has significant faster PL decay than the perovskite one. Additionally, there is an obvious plateau at the low Fermi-level splitting region, indicating an effective decay time of ~10 ns.

Supplementary Fig. 12 shows the variation of TCSPC amplitude of the control film along with the estimated photoinduced carrier concentration n. The amplitude is proportional to n^2, which demonstrates that the defects in the film do not dope the film implying that the densities of electrons and holes before the pulse are not significantly different. Together with the result shown in Fig. 2c (main paper), we conclude that both the control and OAI modified films are intrinsic[6]. In Supplementary Fig. 13, we characterize the defects by FTPS measurements. Results show that the control sample shows an obvious absorption feature

at 1.23 eV in the bandgap. This feature is likely located at the top surface of the perovskite because it disappears after OAI modification.

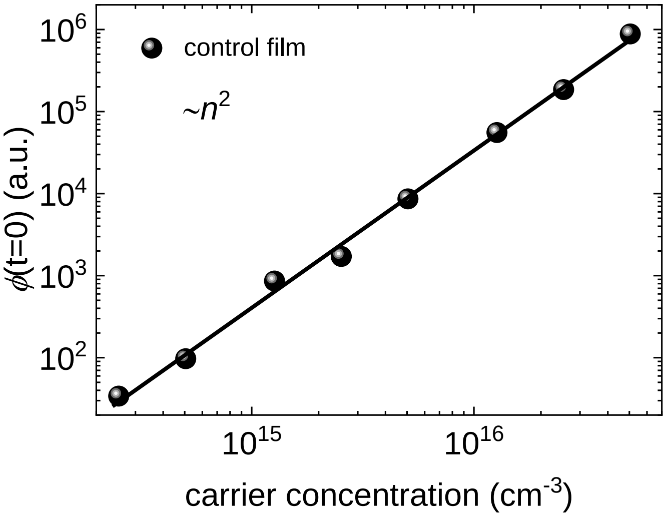

Supplementary Fig. 12 The change of amplitude of tr-PL decay curve (TCSPC setup) for control film along with induced carrier concentration.

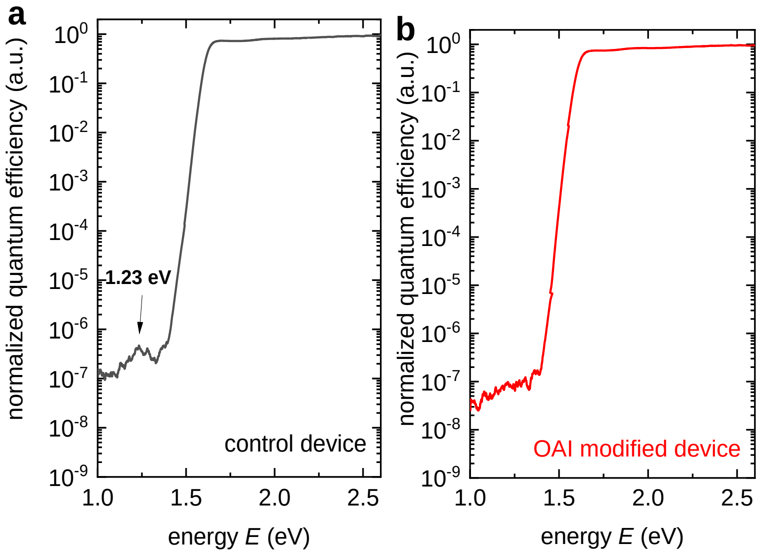

Supplementary Fig. 13 FTPS spectra of **a** control and **b** OAI modified devices.

In Supplementary Fig. 14, we calculated the k_{diff} by using Supplementary Eq. (29). It can be found that the

k_{diff} of the OAI modified film is approximately constant at around 10^{-9} cm^3/s over a large range, implying that the transient follows a power law with approximately power 2 over the nearly the whole decay.

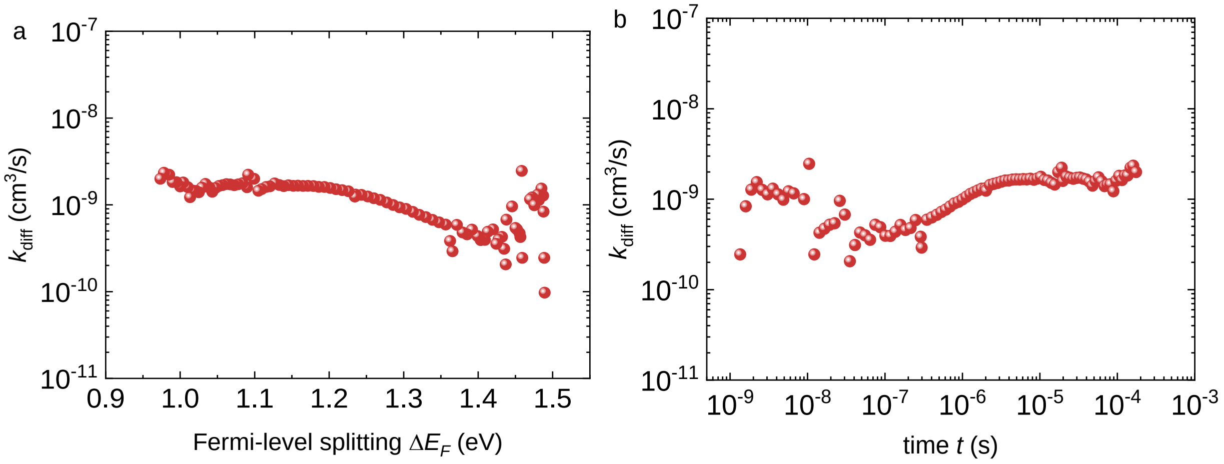

Supplementary Fig. 14 Calculated k_{diff} of OAI modified film based on the tr-PL data from gated CCD setup. (a) k_{diff} vs. ΔE_F. (b) k_{diff} vs. t.

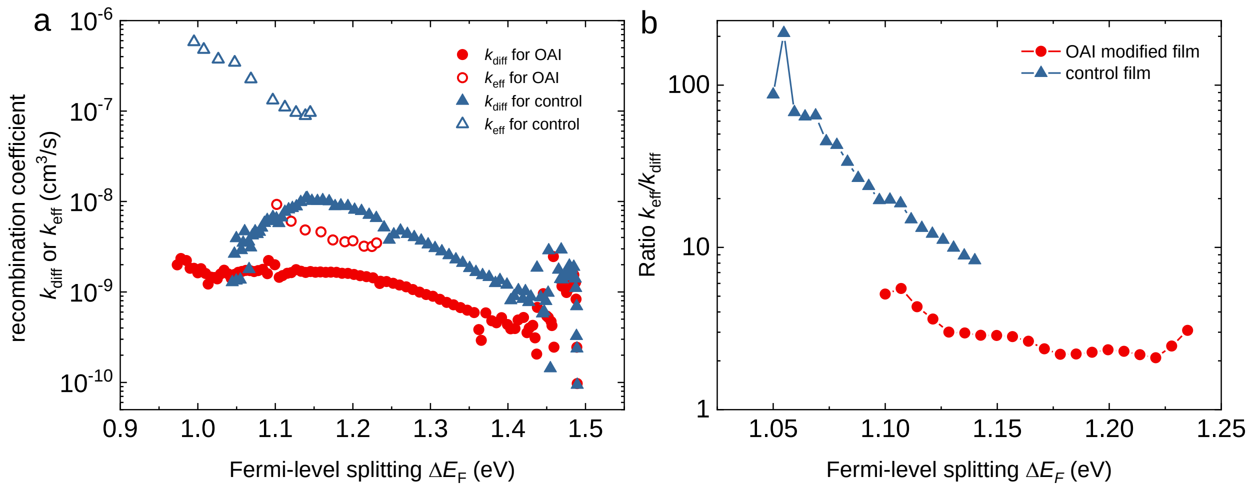

Supplementary Fig. 15 a The effective recombination coefficients from the steady state PL (i.e. k_{eff}) and the ones from the transient PL (i.e. k_{diff}) for both control and OAI modified samples. **b** The corresponding ratio of k_{eff}/k_{diff} for both samples.

Supplementary Fig. 15 shows the effective recombination coefficients of the steady-state PL (called k_{eff})

and the ones from the transient PL (called k_{diff}). Especially the data from the transients are fairly constant vs. Fermi level splitting. Note that “constant” has to be considered relative to the decay time which varies by 4 orders of magnitude. The steady-state data is somewhat less constant and varies significantly, especially in the case of the control film. We also observe that the steady-state data always has higher recombination coefficients than the transients (as shown in panel b), which implies that detrapping slows down the transients relative to the steady-state data. For control sample, detrapping effect is more intense (larger ratio values) due to the higher capture coefficients. If there was essentially no detrapping, we would expect them to be similar. The discrepancy also shows that during the transient, the occupation of the traps must be quite different than during the steady state measurement. Further, the control sample shows both higher values of the recombination coefficient and less constant values. Hence, the recombination is generally higher and the traps are likely less shallow (deeper) than for the passivated sample.

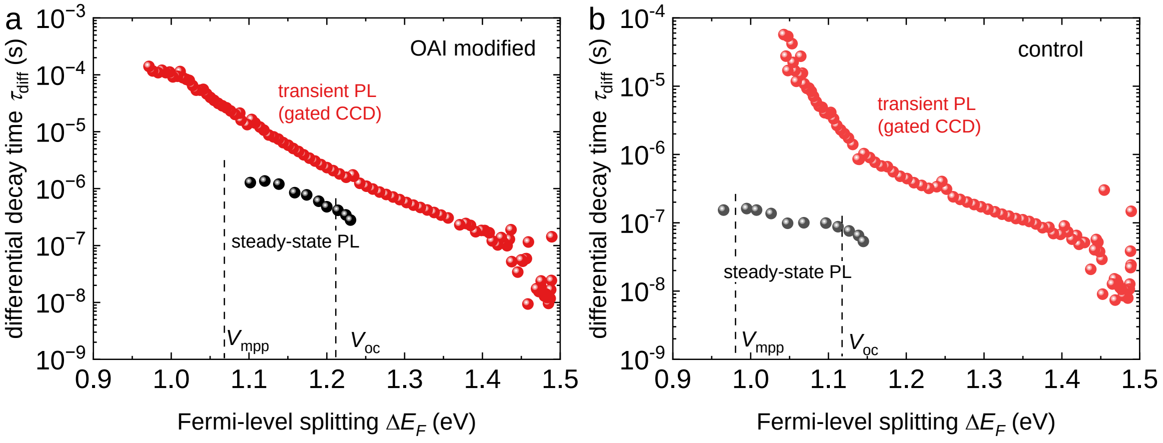

Supplementary Fig. 16 Differential decay time τ_{diff} from both tr-PL and transformed ss-PL results as a function of Fermi-level splitting. **a** OAI modified film. **b** control film.

Here, we make a transformation for the ss-PL data of OAI modified film (which are shown in Fig. 2c) and compare it with the tr-PL data. As we already have ΔE_{F} and illumination intensity (suns), the recombination rate R can be calculated using

$$R = G = \frac{\mathrm{suns}}{\mathrm{d}} \int_{\boldsymbol{E}_{\mathbf{g}}}^{\infty} \phi_{\mathrm{sun}}\, \mathrm{d}E \quad (37)$$

where ϕ_{sun} is the AM 1.5 spectra (global tilt) and G is the generation rate. Substituting the values of R and ΔE_F into Supplementary Eq. (5) and (6), we can obtain the corresponding decay time τ_{diff}. Supplementary Fig. 16 shows that the two curves are nearly parallel rather than overlapping. Such a gap provides evidence for a high density of shallow defects. Interestingly, the τ_{diff} from the transient measurement is larger than the steady-state decay time, because carrier detrapping slows down the decay of the carrier concentration. However, such dynamic processes cannot be detected by steady-state measurement technique. The control sample shows similar results but larger gap between steady-state and transient curves, indicating severer influence of shallow defect (larger density and/or capture coefficients).

As the τ_{diff} continuously increases with lower Fermi-level splitting, it is not possible to acquire a constant lifetime to calculate other related properties like carrier diffusion length. In this case, we suggest using steady-state τ_{diff} at Fermi-level splitting that is equal to the voltage at the maximum power point of the actual solar cell. Thus, in our case, the corresponding τ_{diff} is ~2×10^{-6} s and ~2×10^{-7} s for OAI modified and control sample, respectively. Assuming mobility μ is 2 $\text{cm}^2\text{V}^{-1}\text{s}^{-1}$, we obtain the diffusion length L_D of 3.2 μm (OAI modified sample) and 1 μm (control sample) by using $L_D = \sqrt{\frac{kT}{q}\mu\tau}$.

In the following, we show how to deduce the properties of the shallow defects from fitting a rate equation model to steady state and transient PL data. We have simulated tr-PL and steady state PL data together as shown in Fig. 2b and c in the main paper. Supplementary Fig. 17 and Supplementary Table 2 show the simulation results of shallow defects. For a good fit over the whole range of Fermi level splitting (or carrier density), we need three shallow defects in the film and they have a distance to the nearest band of about 55, 95 and 125 meV. Two possible options for the defects are shown in Supplementary Fig. 17. In general, they should be acceptor like defects close to conduction band or donor like defects close to valence band in order to be consistent with the observation that the tr-PL at early times scales with n^2.

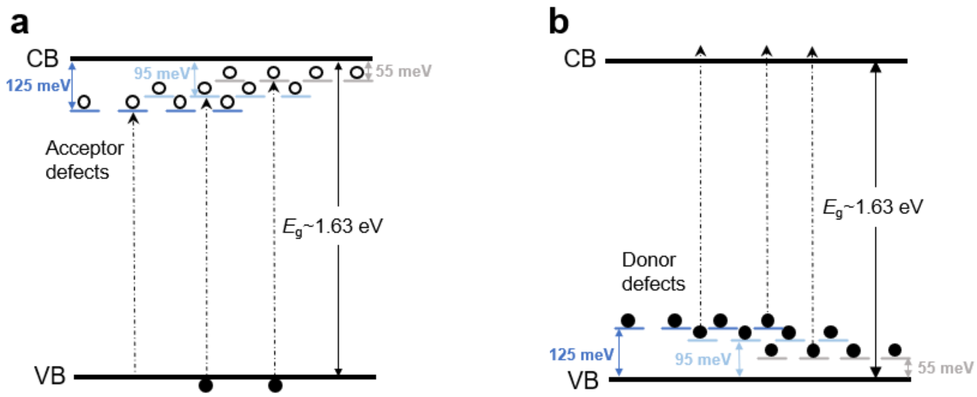

Supplementary Fig. 17 Visualization of different options for the shallow defects. **a** Acceptor-like defects close to the conduction band. **b** Donor-like defects close to the valence band.

It is worth noting that shallow defects not only affect perovskites but also should be considered for silicon materials. As early as 1953-1955, the effect of shallow defects has been observed by Hornbeck and Haynes[7-9] in the single-crystalline silicon and germanium. However, as growth techniques for single-crystalline materials matured, shallow defects do not matter anymore. As for multicrystalline silicon, Daniel Harold Macdonald et al. have observed a long “tail” in the decay, which consequently increased the lifetime in the low carrier density region.[10] Additionally, he has demonstrated that such experimental curves can be fitted well with the Hornbeck-Haynes model, which contains the effect of shallow defects. All these cases suggest the general presence of shallow defects in multicrystalline silicon. Note that we cannot use the exact same equations for our perovskite samples as the Hornbeck-Haynes model was developed for semiconductors with a significant doping density.

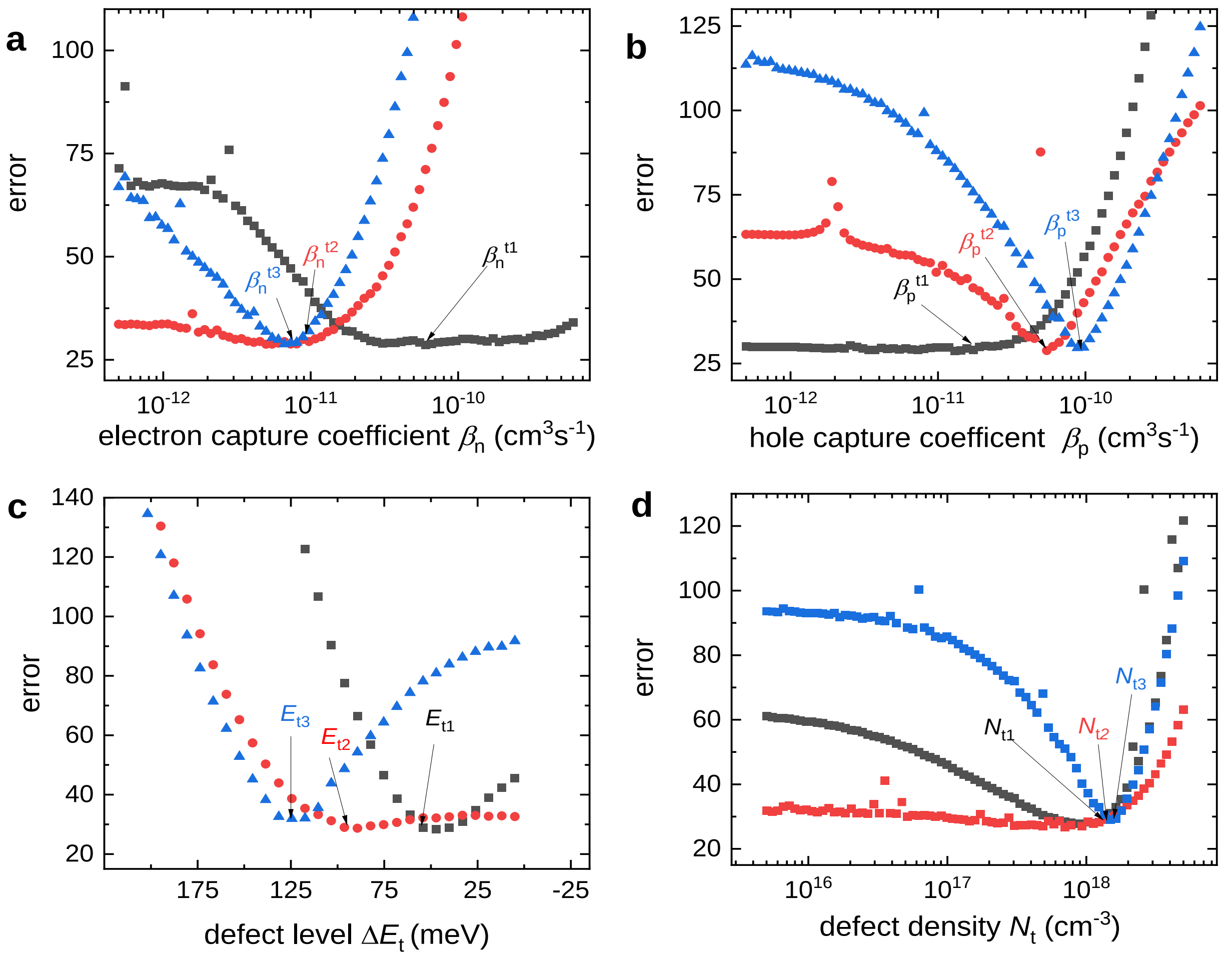

Supplementary Fig. 18 Grid search for the fitted results of the simulated parameters. The arrow positions suggest the values of parameters shown in Supplementary Table 2 (for OAI modified sample).

Here, we discuss the uniqueness of the fitting results. In the simulation, we vary the defect energy levels, defect densities and capture coefficients for the three defects. Here, we have made a grid search to confirm that the fitted values are unique in our case (for OAI modified film). We change the value of each fitting parameter to see the variation of the fitting error, while the other variables are fixed. As shown in the Supplementary Fig. 18, it is obvious that most of the fitted values are around the minimum fitting error, which means that the fitting routine found at least a local optimum, where changing any of the parameter would lead to a loss in fit quality. Only the capture coefficients of Defect 1 could be further reduced (in case of the hole capture) and increased (in case of electron capture) without any effect on the result.

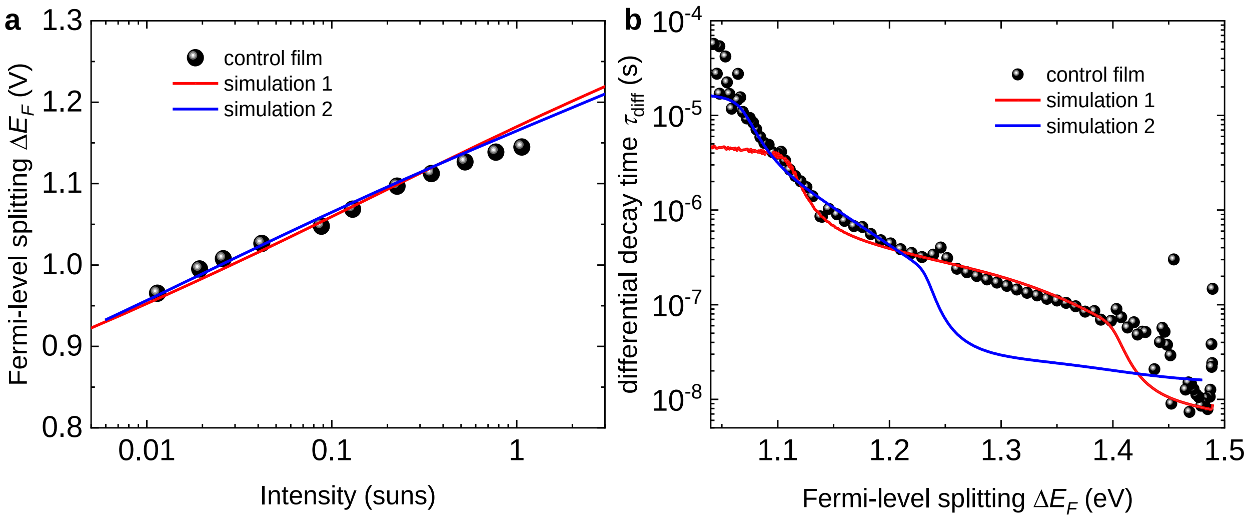

Supplementary Fig. 19 Experimental data of the control sample and corresponding simulated results using the consistent model for both ss-PL and tr-PL. **a** The variation of Fermi-level splitting ΔE_F along with light intensity. **b** The differential decay time τ_{diff} versus Fermi-level splitting ΔE_F. The same to the OAI sample, three defects are used. For simulation 1, a defect with energy level of 1.32 eV is used for simulation. For simulation 2, we further lower the defect energy level to 1.18 eV.

The simulation results of the control film are shown in Supplementary Fig. 19, using the same script as OAI sample. Simulation 1 can generally fit the curve well, except for the low ΔE_F region (< 1.1 eV). The parameter comparison between OAI film and control film (simulation 1) are shown in Supplementary Table 2. The main difference is that the control film has a much deeper defect with a significant higher hole capture coefficient, which is responsible for the ΔE_F loss. Additionally, the difference of emission coefficients is also induced by the different defect energy levels and capture coefficients. If we want to fit the low ΔE_F region better, we need to further lower the defect energy level. In simulation 2, the defect level is assumed deeper (changed from 1.32 to 1.18 eV) while other parameters would also be slightly adjusted by the script automatically. In this case, the fitting for low ΔE_F region is better, indicating that there could be one or more deeper defects existing in the control film. OAI passivation is thought to be able to passivate these deeper defects and lower the capture coefficient.

Supplementary Table 2 Values of parameters related to numerical simulation. The main different parameters between OAI film and control film are highlighted in bold.

	Parameter	OAI film	Control film (Simulation 1)	Method
Basic	Bandgap (eV)	1.63	1.63	EQE inflection point
	Film thickness (nm)	450	450	step profiler
	Radiative recombination coefficient (cm^3/s)#	1×10^{-11}	1×10^{-11}	estimation
	Intrinsic carrier concentration ($1/cm^3$)	4.51×10^{4}	4.51×10^{4}	calculation
	Initial carrier concentration ($1/cm^3$)	1.46×10^{17}	1.46×10^{17}	calculation
	Initial Fermi-level splitting (eV)	1.48	1.48	calculation
Defect 1	Defect level (eV)	1.57	1.56	simulation
	Defect density ($1/cm^3$)	1.32×10^{18}	1.49×10^{18}	simulation
	Electron capture coefficient (cm^3/s)	6.15×10^{-11}	1.22×10^{-10}	simulation
	Hole capture coefficient (cm^3/s)	1.68×10^{-11}	1.59×10^{-10}	simulation
	Electron emission coefficient (1/s)	1.75×10^{7}	2.26×10^{7}	calculation
	Hole emission coefficient (1/s)	1.46×10^{-19}	2.11×10^{-18}	calculation
	Lifetime (s)	5.75×10^{-8}	9.74×10^{-9}	calculation
Defect 2	Defect level (eV)	1.53	1.55	simulation
	Defect density ($1/cm^3$)	1.40×10^{18}	1.58×10^{18}	simulation
	Electron capture coefficient (cm^3/s)	9.26×10^{-12}	1.38×10^{-12}	simulation
	Hole capture coefficient (cm^3/s)	5.36×10^{-11}	6.26×10^{-11}	simulation
	Electron emission coefficient (1/s)	5.20×10^{5}	2.08×10^{5}	calculation
	Hole emission coefficient (1/s)	2.35×10^{-18}	1.02×10^{-18}	calculation
	Lifetime (s)	9.02×10^{-8}	4.7×10^{-7}	calculation
Defect 3	Defect level (eV)	**1.50**	**1.32**	simulation
	Defect density ($1/cm^3$)	1.58×10^{18}	1.61×10^{18}	simulation
	Electron capture coefficient (cm^3/s)	7.65×10^{-12}	2.21×10^{-11}	simulation
	Hole capture coefficient (cm^3/s)	**9.33×10^{-11}**	**9.52×10^{-8}**	simulation
	Electron emission coefficient (1/s)	**1.30×10^{5}**	**320**	calculation
	Hole emission coefficient (1/s)	**1.35×10^{-17}**	**1.62×10^{-11}**	calculation
	Lifetime (s)	8.97×10^{-8}	2.81×10^{-8}	calculation

We use an approximate value of $k_{rad}=1\times10^{-11}$ cm^3/s. From the steady state data, one can estimate k_{rad} to be about ~4×10^{-11} cm^3/s by using the equation $k_{\mathrm{rad}} \times n_{\mathrm{i}}^2 \exp\left(\frac{\Delta E_{\mathrm{F}}}{k_{\mathrm{B}}T}\right) = G$. However, in our simulation, varying k_{rad} in this range only creates a tiny difference (as shown in Supplementary Fig.27).

In Supplementary Fig. 20, we change the energy level of Defect 3 from deep to shallow position. Supplementary Fig. 20a shows ΔE_F increase when the defect level is changed from deep to shallow. This phenomenon is consistent with the consensus that shallow defects do not cause strong carrier recombination, which is related to the "defect-tolerance" of lead halide perovskites[11-14]. In Supplementary Fig. 20b, it is

obvious that any defect level would have a significant influence on the shape of tr-PL curves. When the defect level change from deep to shallow, samples show much longer PL lifetime. Supplementary Fig. 21 suggests the "defect tolerance" more obviously. Here, we change shallow Defect 3, 2 and 1 to deep defects one by one. All other simulated parameters stay the same as in Supplementary Table 2. The results further certify that deep defects would cause more energy loss than shallow defects.

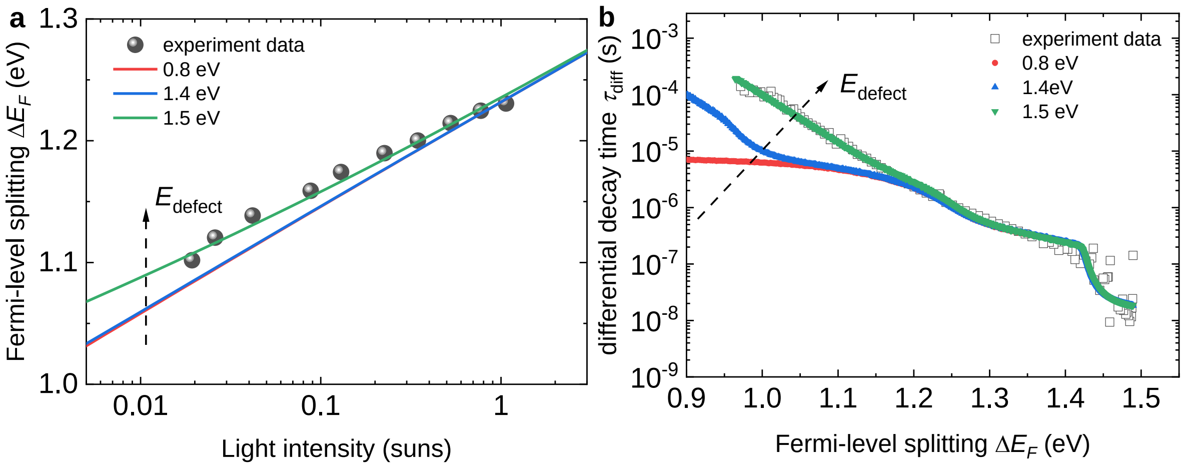

Supplementary Fig. 20 Experimental data of OAI modified sample and the related simulated results using the consistent model for both ss-PL and tr-PL with different energy levels of Defect 3. **a** The variation of Fermi-level splitting ΔE_{F} along with light intensity. **b** The differential decay time τ_{diff} versus Fermi-level splitting ΔE_{F}.

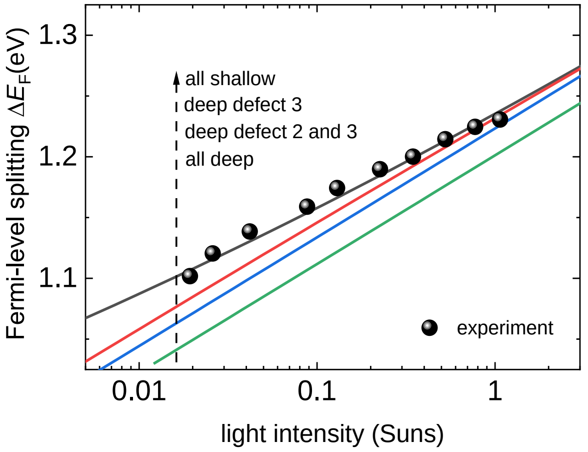

Supplementary Fig. 21 Experiment data and the variation of Fermi-level splitting ΔE_{F} with different number of deep defects (0.8 eV). The results suggest the "defect tolerance" of shallow defects.

Supplementary Fig. 22 shows how different shallow defects affect the τ_{diff} vs ΔE_{F} curves. From Supplementary Fig. 22a, we observe that the shallower defect would dominate the higher ΔE_{F} region. It would determine the shape and the slope of the corresponding part. Supplementary Fig. 22b further indicate that deeper defect should be responsible for the lowered ΔE_{F} in nearly the whole region.

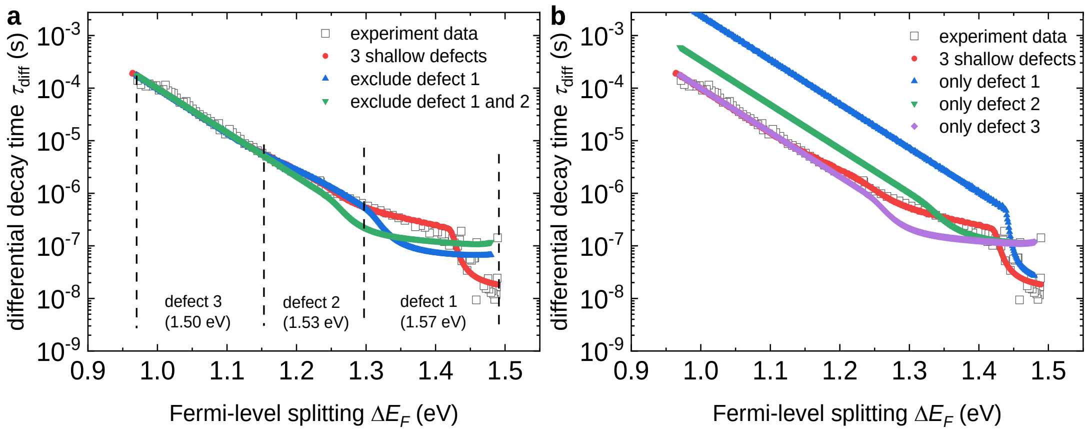

Supplementary Fig. 22 The variation of differential decay time τ_{diff} versus Fermi-level splitting ΔE_{F} with different defects combination. **a** Step-by-step removal of defects. **b** Only activate one defect among the three shallow defects.

Subsequently, we change the density of Defect 3 (i.e. the deepest of the three shallow defects) and the corresponding capture coefficient but fix the effective carrier lifetime and other parameters. Supplementary Fig. 23a shows the result when keeping all defects at the position specified in Supplementary Table 1, while Supplementary Fig. 23b shows the situation where Defect 3 is a midgap defect. According to the results, we can conclude that the defect density of shallow defect has a significant influence on the shape of the decay time curve. As shown in Supplementary Fig. 23a, the differential decay time increases with defect density as also predicted by Supplementary Eq. (27). Furthermore, it continuously increases with lower ΔE_{F}. This behavior is quite different from the situation, where we have one deep defect. In the case of deep defect, the decay time is usually supposed to decrease with increasing defect density as shown in Supplementary Fig. 23b. These simulation results prove that shallow defects have significant influence on tr-PL decay time and the shape of the curve. As shallow defects are widely considered to exist in perovskite

films, they should be considered for quantitative analysis of tr-PL data. In addition, PL decay time is also widely used in the qualitative discussion of film quality. In this case, shallow defects also need to be treated with caution. The longer PL decay time may result from a decrease of deep defect density, but it may also result from an increase of shallow defect density. Obviously, the latter is not an evidence of film quality improvement.

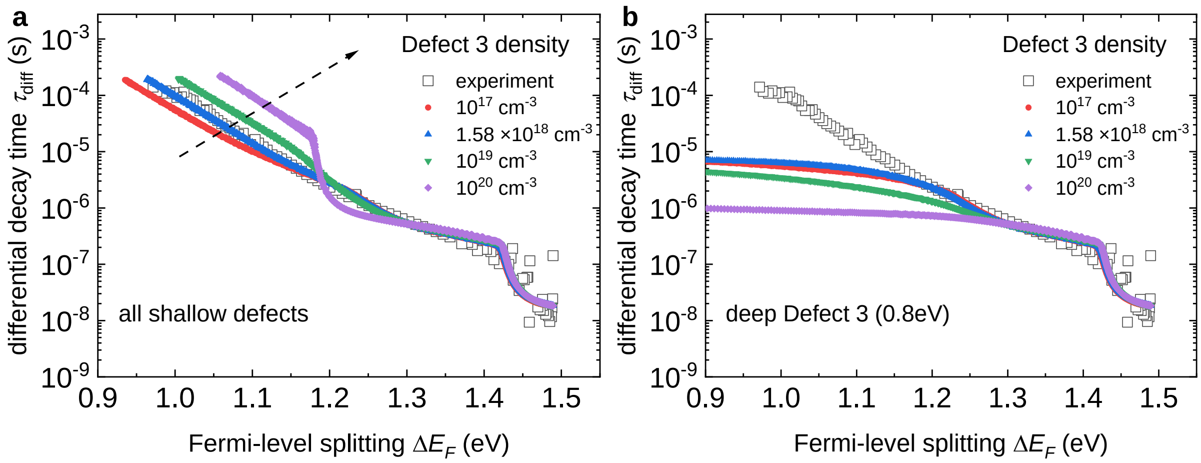

Supplementary Fig. 23 Experiment data and the variation of differential decay time τ_{diff} versus Fermi-level splitting ΔE_F with different defect densities of Defect 3 while keeping the effective carrier lifetime constant. **a** In the case of all are shallow defects. **b** In the case of Defect 3 is deep defect (0.8 eV).

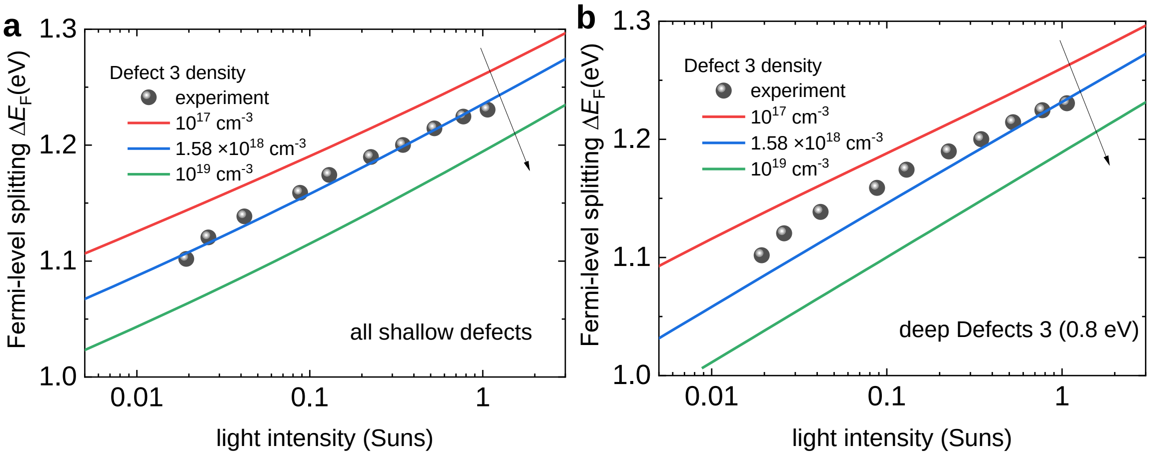

Supplementary Fig. 24 Experiment data and the variation of Fermi-level splitting ΔE_F with different defect densities of Defect 3. **a** In the case of all are shallow defects. **b** In the case of Defect 3 is a deep defect (0.8 eV).

Supplementary Fig. 24 shows the variation of simulated Fermi-level splitting ΔE_F with different defect densities. In order to accurately show the influence of defect density, we removed the restriction on effective carrier lifetime. That means capture coefficient won't simultaneously change with density, so the effective carrier lifetime would directly decrease with increase of defect density. Supplementary Fig. 24 clearly shows that a high deep-defect density can significantly deteriorate the ΔE_F at a given steady state light intensity, while the shallow defect density shows less impact with the same value.

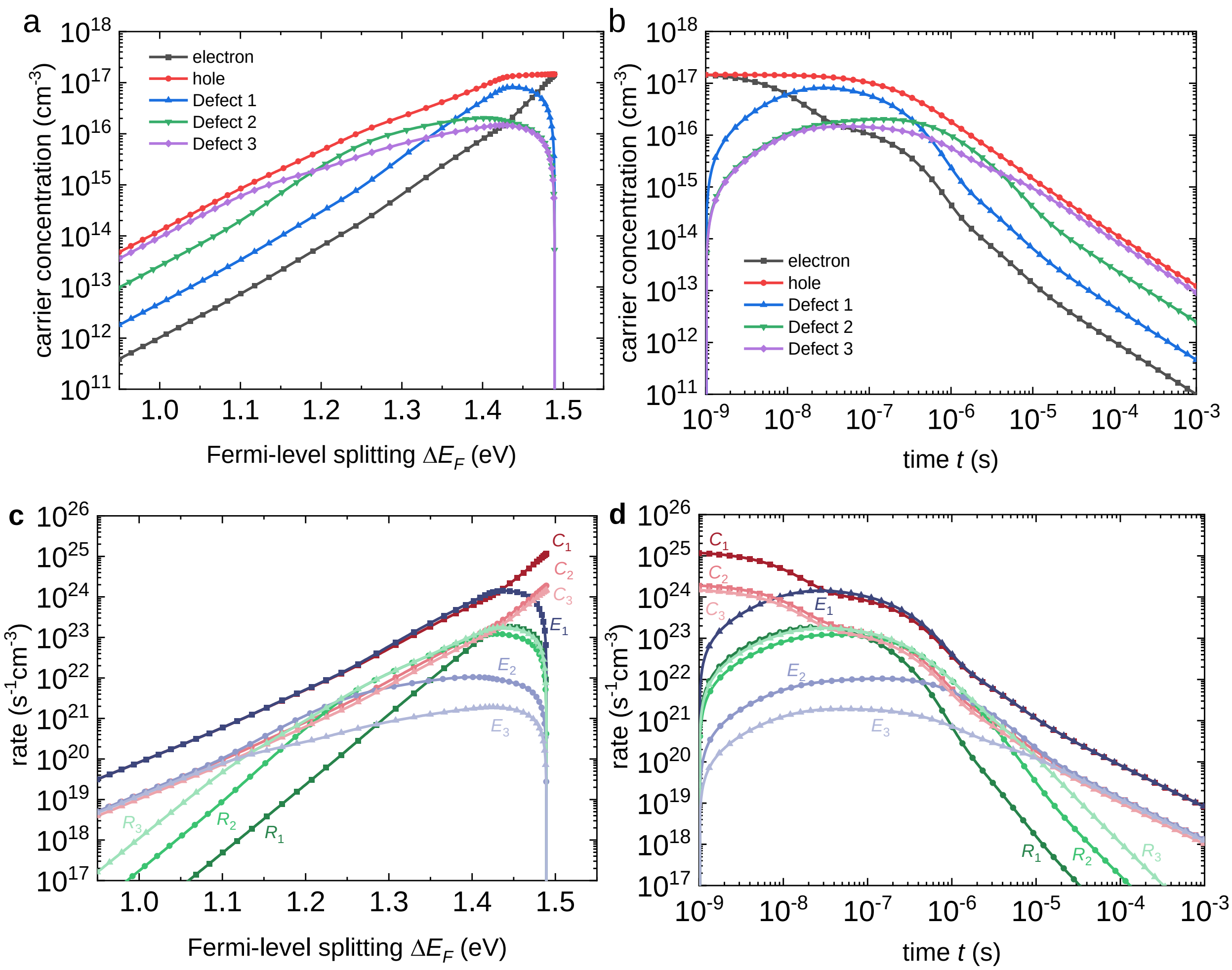

Supplementary Fig. 25 Simulated carrier and defect behavior during the transients. **a, b** Variation of free carrier and defect trapped carrier concentration along with Fermi-level splitting and time. **c, d** Capture rate (C_1, C_2 and C_3), recombination rate (R_1, R_2 and R_3) and emission rate (E_1, E_2 and E_3) of defects as a function of Fermi-level splitting and time. Please note that capture rate $= \beta_{\mathrm{n}}^{\mathrm{t}} n(N_{\mathrm{t}} - n_{\mathrm{t}})$, recombination rate $= \beta_{\mathrm{p}}^{\mathrm{t}} p n_{\mathrm{t}}$, emission rate $= e_{\mathrm{n}}^{\mathrm{t}} n_{\mathrm{t}} = \beta_{\mathrm{n}}^{\mathrm{t}} n_1 n_{\mathrm{t}}$. More details can be found in Supplementary Note 6.

Supplementary Fig. 25 shows the carrier behavior under the influence of shallow defects. Supplementary Fig. 25a and b show that electron concentration decreases more sharply than the hole concentration, especially in the first 30 ns. This is because a large number of free electrons has been captured by shallow defects in the beginning (we assume that defects are all acceptor-like defects close to the conduction band in our simulation). As for the trapped carriers, we observe a carrier transfer process from shallower to deeper defects. Supplementary Fig. 25c and d show the capture, recombination and emission rates of defects. All capture rates show a sharp decrease in the first 30 ns, which is consistent with the variation of free electron concentration. The emission rates show the same tendency with corresponding trapped carrier concentrations. When the Fermi-level splitting is small, the emission rates equal the capture rates. As for the recombination rate, all the defects show a similar value in the beginning but shallower defects will show lower values at later times.

To investigate how a deep defect affect the recombination, we add a parameter τ_{bulk} in the simulation to represent carrier lifetime of a deep defect. All other parameters stay the same as in Supplementary Table 2. Supplementary Fig. 26 shows that the τ_{bulk} of our sample should be at least 10^{-3} s, suggesting that deep defect has negligible effect on the recombination dynamic.

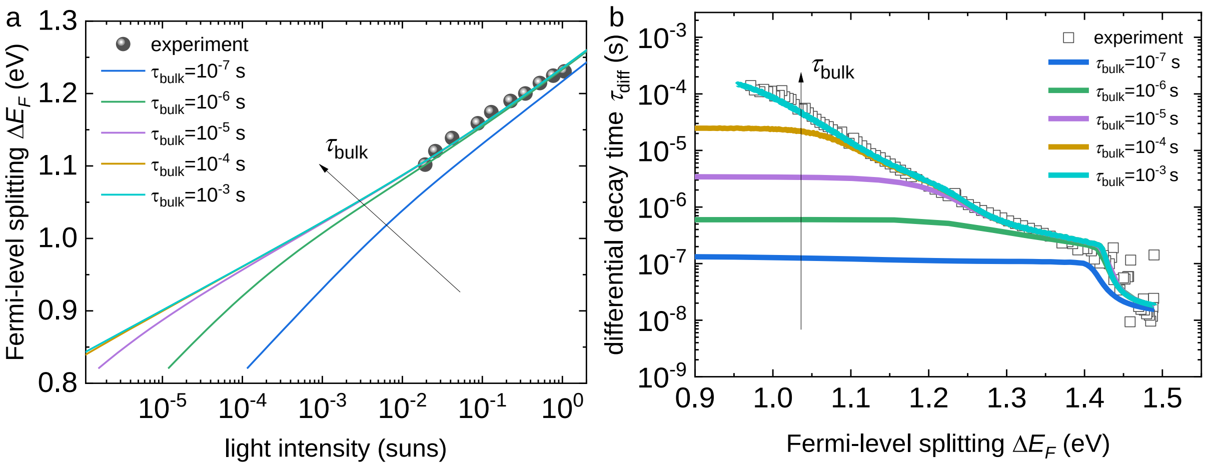

Supplementary Fig. 26 Experimental data and simulated results using the consistent model for both ss-PL and tr-PL. The simulation was performed with 3 shallow defects and one deep defect. **a** The variation of Fermi-level splitting ΔE_{F} along with light intensity. **b** The differential decay time τ_{diff} versus Fermi-level splitting ΔE_{F}.

The radiative recombination coefficient k_{rad} used in the simulation shows negligible influence on the ΔE_F versus light intensity plot (ss-PL related). But it has slight influence on the tail part of τ_{diff} versus ΔE_F curve. As shown in Supplementary Fig. 27, the τ_{diff} would decrease slightly in the range of 1.4~1.5 eV when the k_{rad} value increase from 1×10^{-11} to 1×10^{-10} $cm^{-3}s^{-1}$.

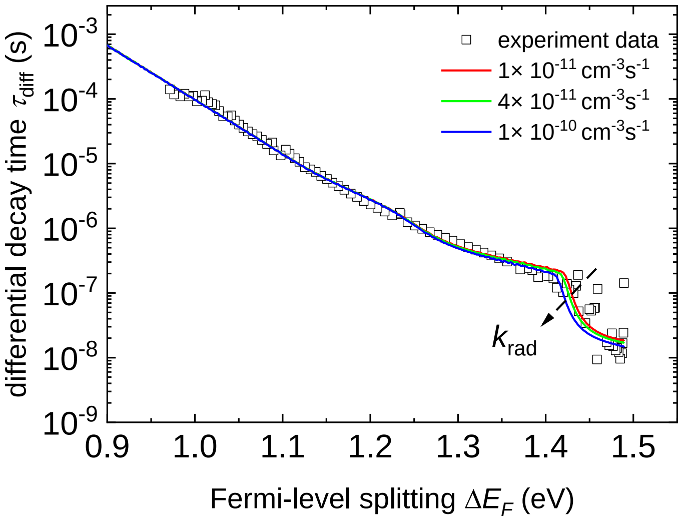

Supplementary Fig. 27 The influence of radiative recombination coefficient on differential decay time τ_{diff}. The simulations are performed with the consistent model for both ss-PL and tr-PL.

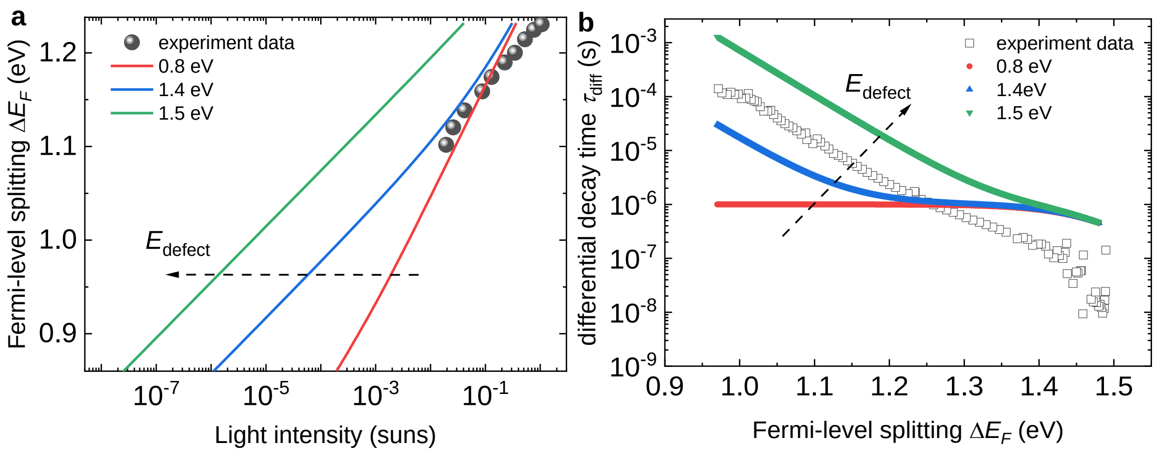

Supplementary Fig. 28 Simulation results for an intrinsic semiconductor with one defect varying defect energy levels under fixed low defect density (10^{13} cm^{-3}) and fixed carrier lifetime (1 μs). A consistent model for both ss-PL and tr-PL is used for simulation. Experimental data of our perovskite sample are also presented for comparison. **a** The variation of Fermi-level splitting ΔE_F along with light intensity in ss-PL. **b** The differential decay time τ_{diff} versus Fermi-level splitting ΔE_F in tr-PL.

Here, we assume an intrinsic semiconductor with a very low defect density of 10^{13} cm^{-3}, which is the normal case for high quality traditional semiconductors. In Supplementary Fig. 28, we change the defect energy level from deep to shallow and simulate the result using our consistent model. Supplementary Fig. 28a shows that the Fermi-level splitting ΔE_F tends to be smaller for deeper defect, which is more obvious under low light intensity. As for the differential decay time τ_{diff}, it exists a plateau (equal to the fixed carrier lifetime) in the deep defect case (0.8 eV). However, in the case of shallow defect, τ_{diff} will go up infinitely in low ΔE_F region. The defect located at 1.4 eV behaves as a shallow defect for low ΔE_F (when $n < n_1$) and as a deep defect for medium ΔE_F ($n > n_1$) until radiative recombination takes over at high ΔE_F.

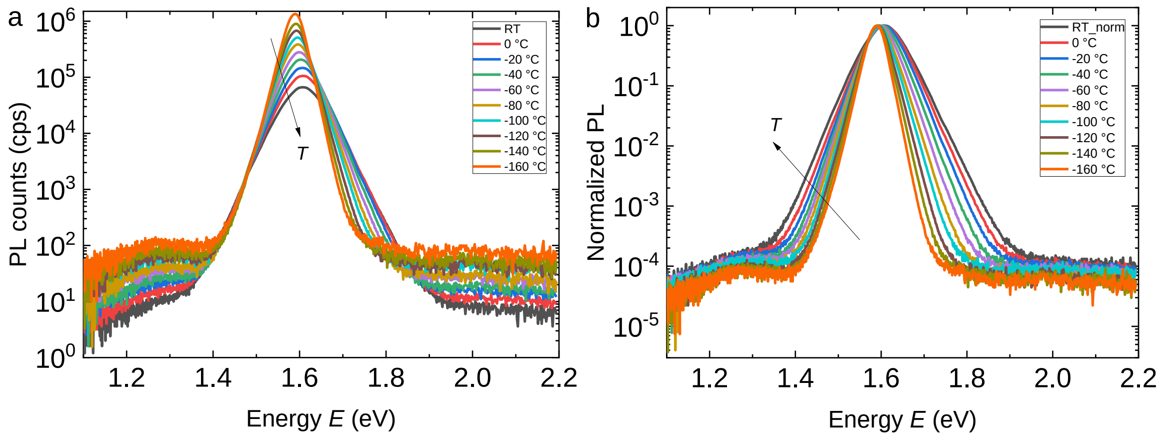

Supplementary Fig. 29 Temperature-dependent PL spectra of OAI film sample. (a) Original PL spectra. (b) Normalized PL spectra.

Here, we discuss the behavior of lifetime and luminescence quantum efficiency under low temperatures. In the case of recombination is dominated by shallow defect ($n_1 > n, p$), the steady-state recombination rate would be given by $R_{\mathrm{SRH}} = \frac{np}{n_1 \tau_{\mathrm{p}}}$. The internal luminescence quantum efficiency would then be given by $Q_i^{\mathrm{lum}} = \frac{R_{\mathrm{rad}}}{R_{\mathrm{rad}} + R_{\mathrm{SRH}}} = \frac{k_{\mathrm{rad}} n_1 \tau_{\mathrm{p}}}{k_{\mathrm{rad}} n_1 \tau_{\mathrm{p}} + 1}$. In the limit, where non-radiative processes are still dominant, this would simplify to $Q_i^{\mathrm{lum}} = k_{\mathrm{rad}} n_1 \tau_{\mathrm{p}}$. The key question determining the temperature dependence of the PL intensity is therefore how the product $k_{\mathrm{rad}} n_1 \tau_{\mathrm{p}}$ depends on temperature. The radiative recombination coefficient does not seem to have a strong and obvious temperature dependence, when looking at the

experimental data shown in ref.[15]. It showed a strong temperature dependence of $k_{\mathrm{rad}}n_i^2$ but it seems to be entirely dominated by n_i^2. The parameter n_1 has a temperature dependence dominated by the trap depth E_C - E_t, i.e. $n_1 = N_C\exp[-(E_C - E_t)/kT]$.

The hole lifetime τ_p however has most likely a temperature dependence that makes τ_p get longer with lower temperatures, i.e. $\tau_p \propto \exp[E_a/kT]$, where $E_a > 0$ is an activation energy for the recombination process. This is a behavior typical for multiphonon recombination in semiconductors.[16, 17] It was found experimentally for instance for III-V semiconductors and has been derived theoretically within the harmonic oscillator approximation [18] but also for anharmonic semiconductors such as halide perovskites [19]. It originates from the fact that for an electron or hole capture event to happen, the vibrational ground state of the upper level (say the defect) has to transfer to a vibrationally excited state of the electronic ground state (say the valence band). This transfer happens at low temperatures by tunneling and at medium or high temperatures by a combination of thermal activation and tunneling. At room temperature it is most likely that the fastest transfer of charge to the vibrationally excited state of the electronic ground state involves thermal activation to reduce the tunneling barrier. Thus, non-radiative carrier capture events are typically (but not necessarily) thermally activated with an activation energy that is usually small relative to the band gap. Thus, it may well be in a similar range as the trap depth of a shallow trap. Thus, the product $k_{\mathrm{rad}}n_1\tau_p$ may increase or decrease with temperature even for shallow traps. If the activation energy for the hole lifetime is higher than the absolute value of the trap depth, the net effect would be an increase in the luminescence intensity with reduced temperature, as observed in Supplementary Fig. 29.

Note that this trend would be the same for deep defects. In this case, $Q_i^{\mathrm{lum}} \approx k_{\mathrm{rad}}n\tau_{\mathrm{deep}} = k_{\mathrm{rad}}G\tau_{\mathrm{deep}}^2$, where we again assume that the luminescence quantum efficiency is significantly smaller than 1. Then at a given steady state generation rate G, the temperature dependence of the lifetime of the deep defect would determine the overall temperature dependence. As the lifetime τ_{deep} of the deep defects is equally likely getting longer with lower temperatures as the lifetime of a shallow defect, the trend would again be qualitatively the same. The fact that we obtain a τ_{deep}^2 term here, is not necessarily helpful to distinguish whether the recombination is dominated by shallow or deep defects as we don't know the activation energy

of the processes. Thus, we conclude that the temperature dependence of steady-state PL is not likely to provide additional insights into the recombination mechanism. This is because we have no way to a priori know the exact temperature dependence of the electron and hole lifetimes (or capture coefficients) but we also cannot ignore them as they will likely dominate the temperature dependence of the luminescence intensity. Noted that such situation is also true to the temperature dependence of transient PL, because τ_{diff} also depends on the hole lifetime (for a shallow trap close to the conduction band) or the electron lifetime (for a shallow trap close to the valence band) as shown in Supplementary Eq. (27).

Supplementary Note 3 Influence of charge extracting layers

Fig. 3a in the main paper shows quasi-Fermi-level splitting of samples with different stacks. The calculation is based on the steady-state PL results of which original PL curves are shown in Supplementary Fig. 30. The peaks of each steady-state PL spectra are adopted for ΔE_F calculation. Supplementary Fig. 31 shows absorptance spectra of these samples measured by UV-VIS spectrophotometer. Obviously, different stack samples have different absorptance. So the influence of absorptance must be taken into account for the ΔE_F calculation. More details about calculation can be found in Methods section. From above results, we can also obtain the photoluminescence quantum yield Q_e^{lum} for different layer stacks, as shown in Supplementary Fig. 32.

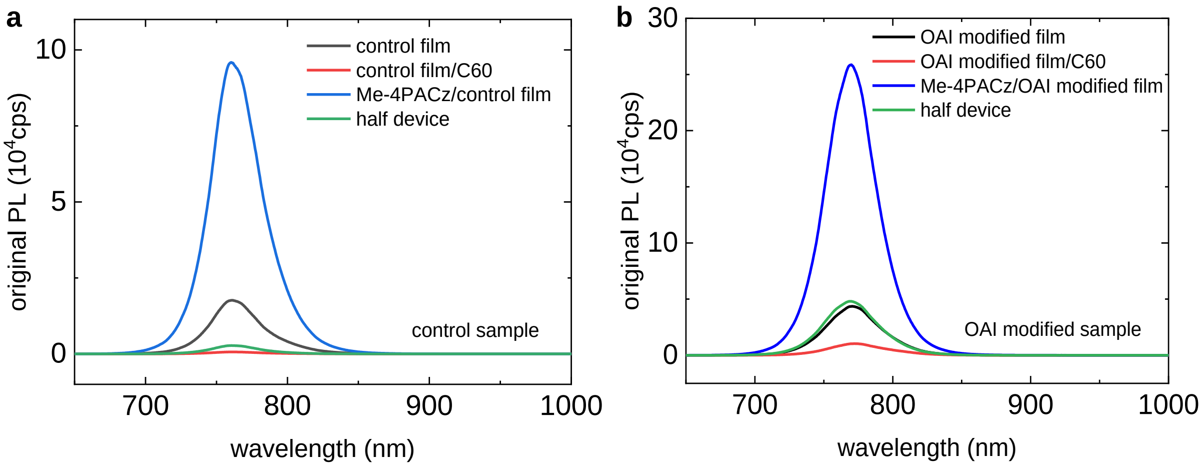

Supplementary Fig. 30 Original steady-state PL spectra of **a** control and **b** OAI modified samples used for quasi-Fermi level splitting ΔE_F calculation of which shown in main paper Fig. 3a.

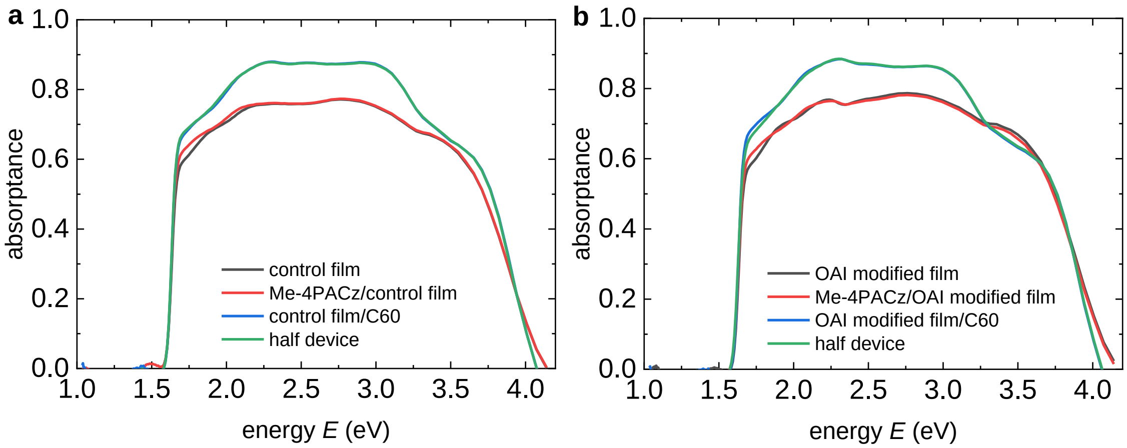

Supplementary Fig. 31 Absorptance spectra of **a** control and **b** OAI modified samples.

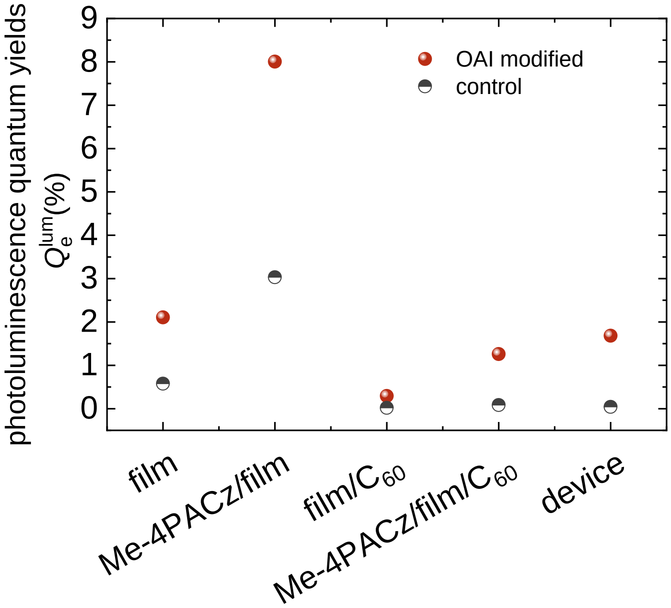

Supplementary Fig. 32 Photoluminescence quantum yield Q_e^{lum} for the control and OAI modified samples with different layer stacks using the quasi-Fermi-level splitting ΔE_{F} data shown in Fig. 3a. The relationship between Q_e^{lum} and ΔE_{F} is described in Supplementary Eq. (36).

In Fig. 3a and b (main paper), we have discussed the influence of charge extracting layers based on the gated CCD results. In Supplementary Fig. 33, we plot the TCSPC and gated CCD results together. The TCSPC results are also splitted into 4 parts by changing laser intensity using OD filters. From TCSPC

curves in Supplementary Fig. 33a, c, e, we can also demonstrate that OAI modified films/C_{60} sample shows much faster decay than the other two stacks, which is matched well with gated CCD results. From the corresponding τ_{diff} vs. ΔE_F curves, we can further make sure that the gated CCD and TCSPC results have a high degree of matching in the high Fermi-level splitting region (e.g. 1.2-1.5 eV). but at lower region (e.g. <1.1 eV), gated CCD data is more accurate.

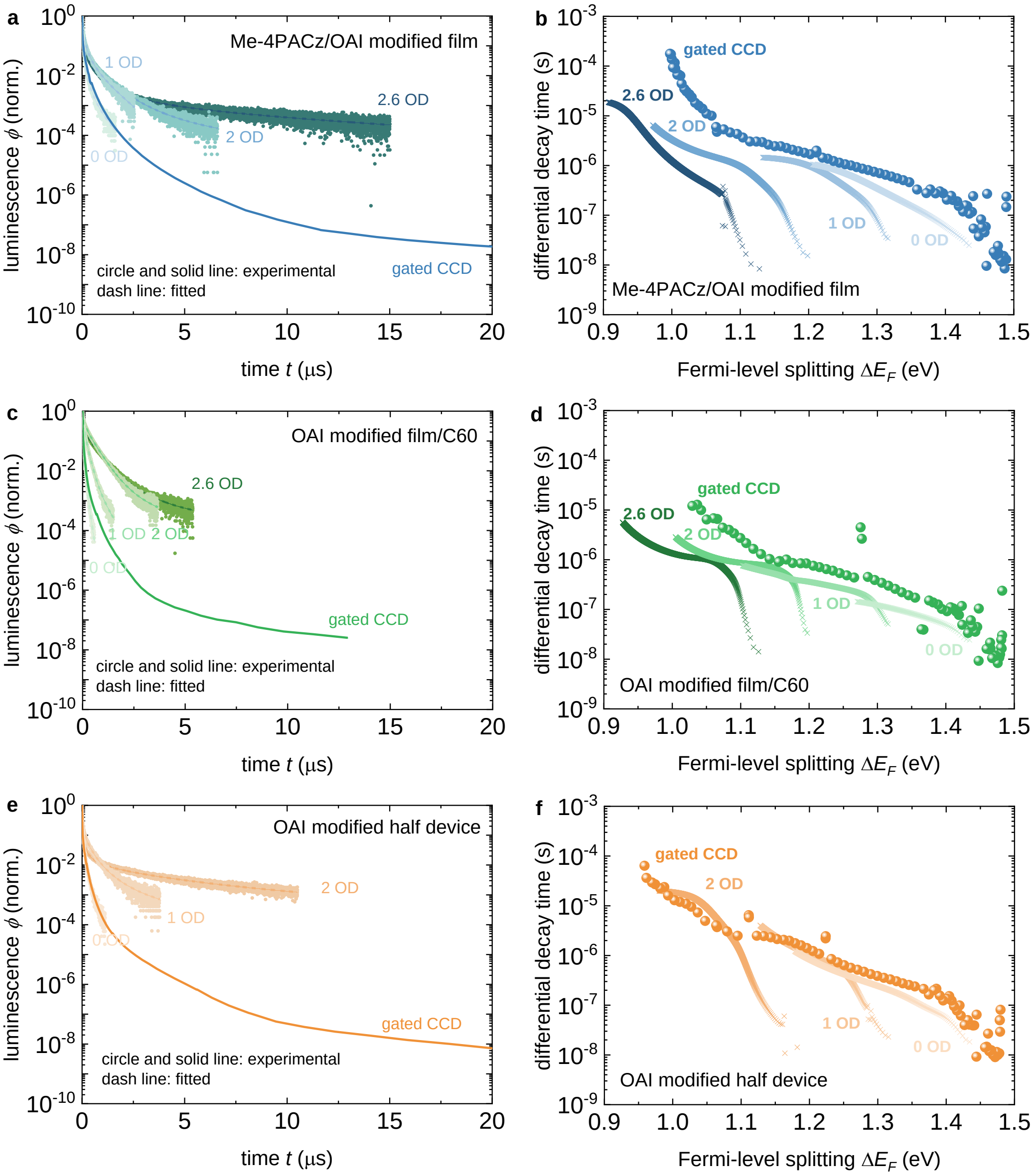

Supplementary Fig. 33 Tr-PL decay curves and the corresponding τ_{diff} changing with ΔE_F for different layer stack samples. **a, b** ITO/Me-4PACz/OAI modified film sample, **c, d** OAI modified film/C_{60} sample and **e, f** ITO/Me-4PACz/OAI modified film/C_{60} half device sample.

Supplementary Note 4 Device characteristics

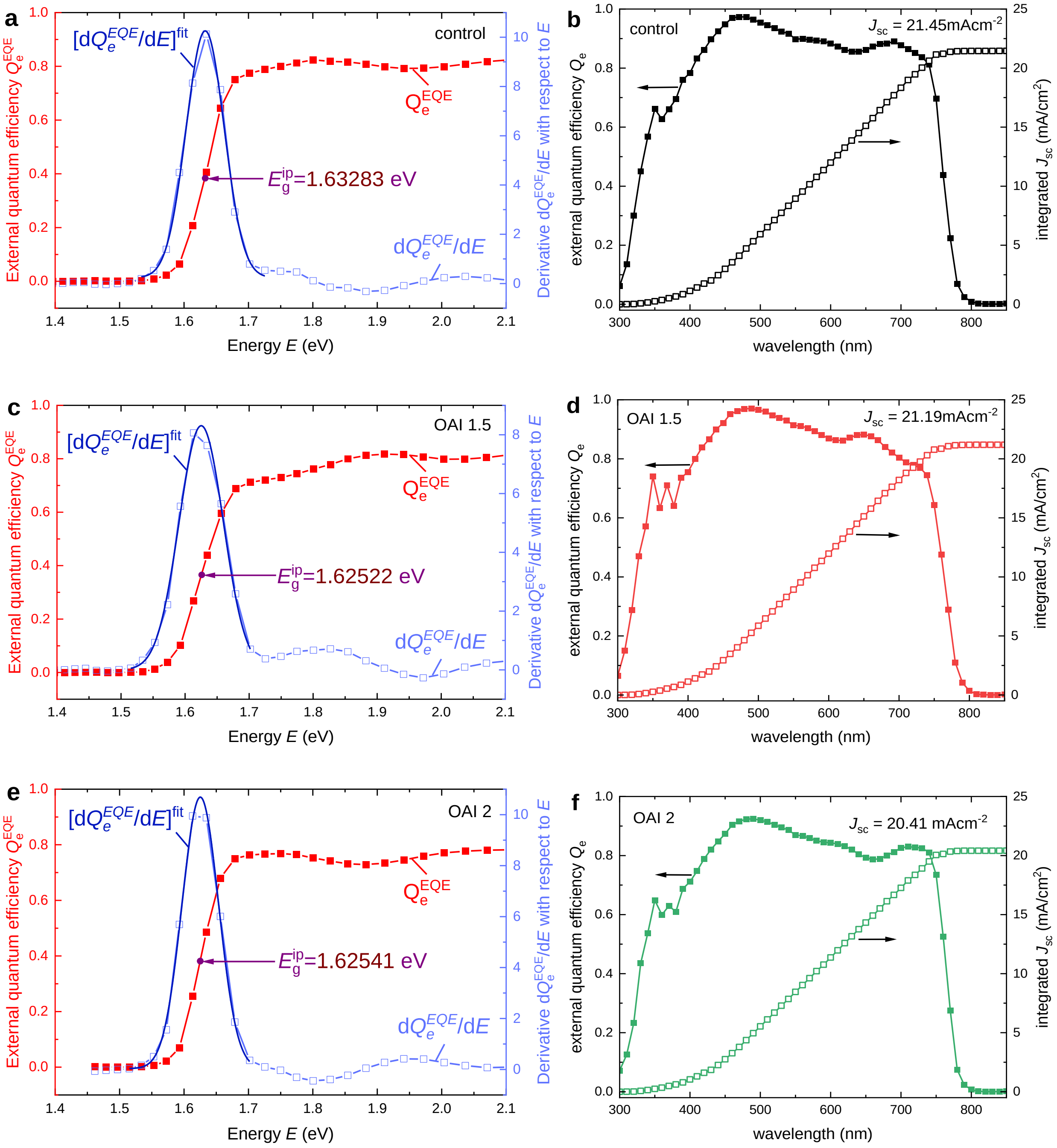

Supplementary Fig. 34 Calculation of bandgap by inflection method for **a** control, **b** OAI modified sample with 1.5 mg/mL solution and **c** OAI modified sample with 2 mg/mL solution. EQE spectra and the corresponding integrated J_{sc} for **d** control, **e** OAI modified sample with 1.5 mg/mL solution and **f** OAI modified sample with 2 mg/mL solution.

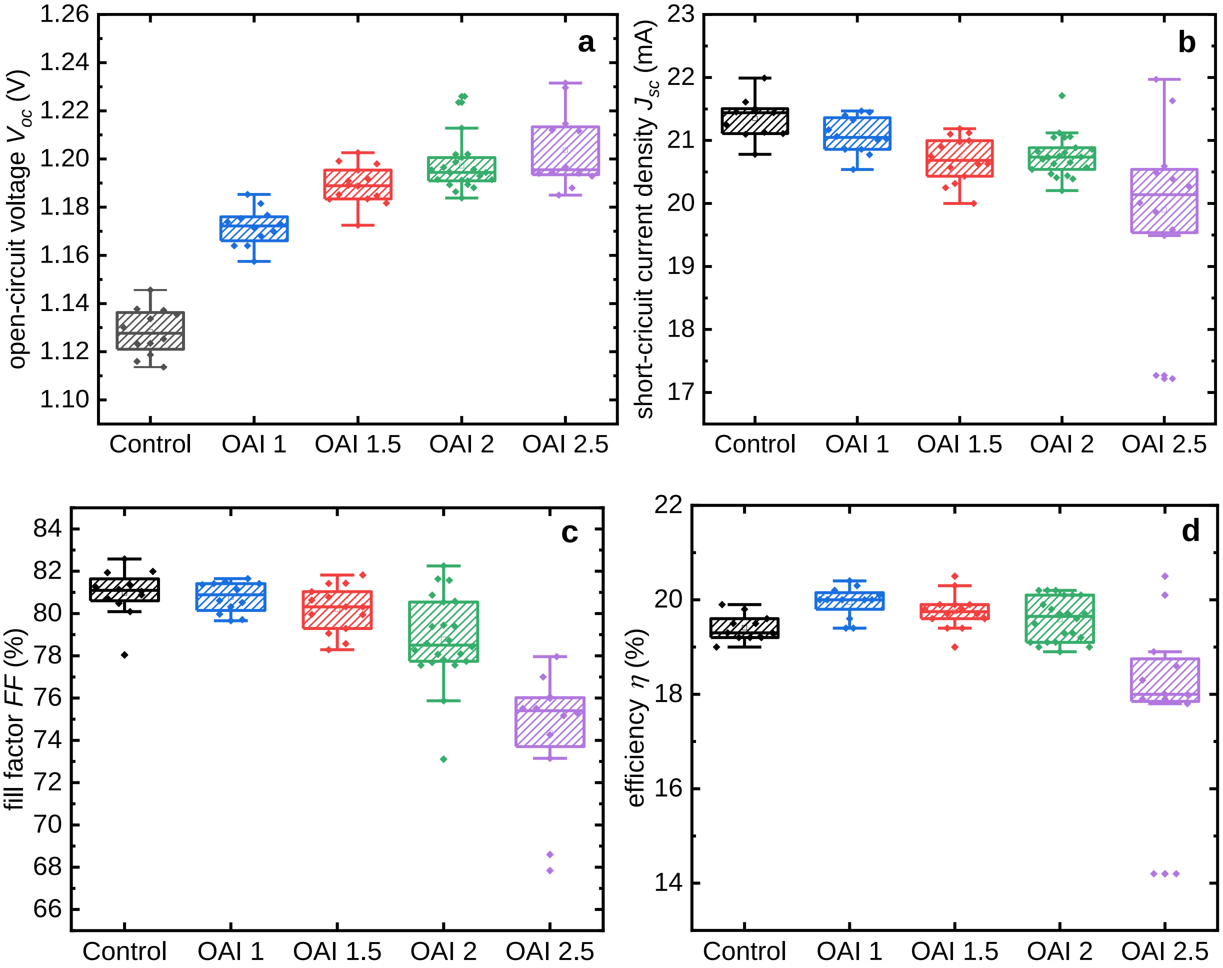

Supplementary Fig. 35 Statistical distribution of **a** open-circuit voltage **b** short-circuit current **c** fill factor and **d** efficiency of control cells and OAI modified cells with OAI concentration from 1 to 2.5 mg/mL (named OAI 1, OAI 1.5, OAI 2, OAI 2.5 in figures). The data points were based on forward *JV* scan results of normal area device (0.16cm^2) and all the devices are independent. As for the box-plotting, the box contains the values from upper to lower quartiles. The lines outside the box indicate the 1.5x interquartile range, as well as the line inside the box is the median line. The open square inside the box is the mean value. The sample size for each group is 12 (control), 12 (OAI 1), 14 (OAI 1.5), 22 (OAI 2) and 12 (OAI 2.5), separately.

Devices with two different active areas were finally fabricated with ITO/Me-4PACz/

$Cs_{0.05}FA_{0.73}MA_{0.22}PbI(I_{0.78}Br_{0.22})_2$ /C_{60}/BCP/Ag inverted architecture. The normal area (0.16 cm^2) devices were used to show reliable variation tendency with the optimization. Unless otherwise noted, normally, the area of devices is 0.16 cm^2 in this paper. As most reported high performance perovskite solar cells are based on small area (< 0.1 cm^2)[20, 21], we also prepared small area (0.06 cm^2) device to show the upper limit of performances.

Supplementary Fig. 34 shows EQE spectra of control and modified devices. We determine the bandgap of devices based on the inflection method[22, 23]. The bandgap values show slight decrease after OAI modification, but in general they are all around 1.63 eV. The control device shows the largest EQE, of which maximum point is over 97% at ~470 nm. After modified by OAI solution (1.5 mg/ml), the EQE decrease a bit at long-wavelength region (~700 nm), resulting in an integrated J_{sc} decrease from 21.45 to 21.19 $mAcm^{-2}$. By further increasing OAI concentration to 2mg/ml, the EQE decrease in the whole region, and the integrated J_{sc} further decrease to 20.41 $mAcm^{-2}$. The statistical data shown in Supplementary Fig. 35 also proves the decrease of J_{sc}. Even so, the V_{oc} enhance more significantly. When OAI concentration is 1-2 mg/mL, it is easier to get high quality device with good PCE and V_{oc}.

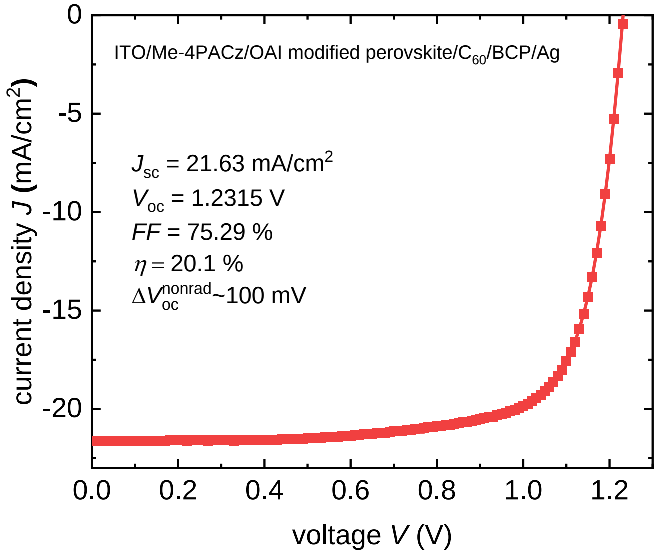

Supplementary Fig. 36 *JV* curve of OAI modified device under forward scan with highest open-circuit voltage over 1.23V and lowest nonradiative recombination loss ~100 mV.

Supplementary Fig. 36 shows the *JV* curve of the OAI modified cell with highest open-circuit voltage over

1.23 V. Voltage loss of a device can be separated into two parts[23],

$$\Delta V_{\mathrm{oc}} = \Delta V_{\mathrm{oc}}^{\mathrm{rad}} + \Delta V_{\mathrm{oc}}^{\mathrm{nonrad}} \tag{38}$$

where $\Delta V_{\mathrm{oc}}^{\mathrm{rad}}$ is the radiative loss which is due to the difference between the ideal SQ value and the radiative value. And $\Delta V_{\mathrm{oc}}^{\mathrm{nonrad}} = V_{\mathrm{oc}}^{\mathrm{rad}} - V_{\mathrm{oc}}$ which is caused by nonradiative recombination loss. $\Delta V_{\mathrm{oc}}^{\mathrm{nonrad}}$ is the main source of energy loss and can be decreased by the defect passivation. According to the method proposed by ref [23], the $\Delta V_{\mathrm{oc}}^{\mathrm{nonrad}}$ of this cell is calculated to be ~100 mV. By contrast, the control cell has ~208 mV nonradiative recombination loss.

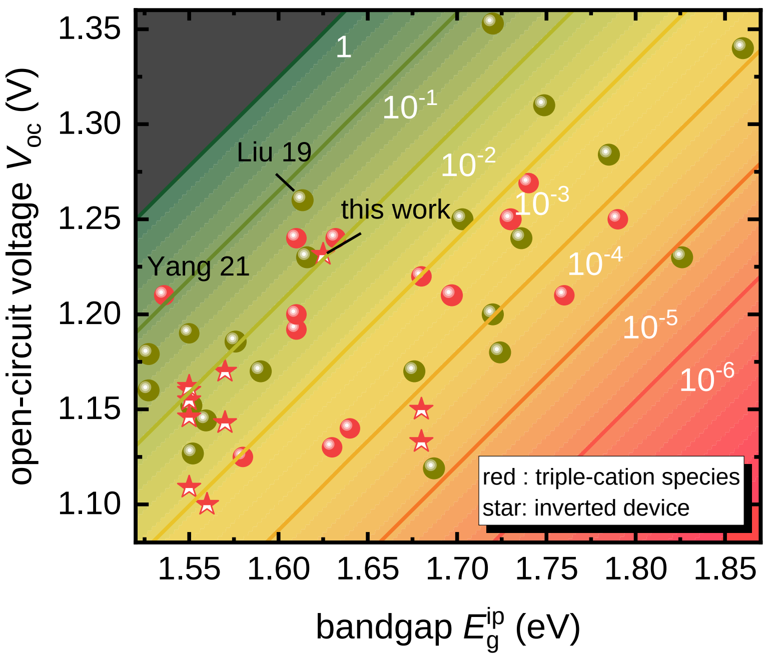

Supplementary Fig. 37 Open-circuit voltage vs. bandgap[20, 21, 24-28].

Supplementary Fig. 37 shows V_{oc} versus bandgap of some reported perovskite solar cells. The reference lines show the level of photoluminescence quantum efficiency which is calculated by a step function absorptance. The device from Yang *et al*. shows the top quantum efficiency of ~10%[28]. As for wide bandgap devices (> 1.6 eV), Liu *et al*. have reported a high value > 5%[24, 27]. Our device has a quantum efficiency ~2%, which is higher than the majority of triple-cation perovskite solar cells, especially in the field of inverted perovskite solar cells.

Supplementary Fig. 38 shows other two representative *JV* curves. When device areas are fixed at 0.16 cm^2, the PCE of OAI modified sample can reach ~20.5%, which is much higher than the control sample (~19.6%). Under small area (0.06 cm^2) condition, the OAI modified sample can get high PCE over 21% (as shown in Fig. 4a in the main paper). Besides, control cells show no obvious hysteresis, while high V_{oc} cells modified by OAI always show better FF and PCE under forward scan condition. The stabilized power output is closer to the power of the forward scan direction as well. Supplementary Fig. 39a and b show the maximum efficiency and open-circuit voltage tracking results. After a sharp increase in the initial stage, the efficiency of both control and OAI modified samples keep stable during the measurement. The control sample show a stable efficiency of ~19.4%, while OAI modified sample is ~20.2%. Different from the efficiency, the open-circuit voltage of the samples slightly decrease first and then keep stable. The V_{oc} of OAI modified sample can still keep stable with ~1.19 V, which is much higher than control sample (~1.12 V).

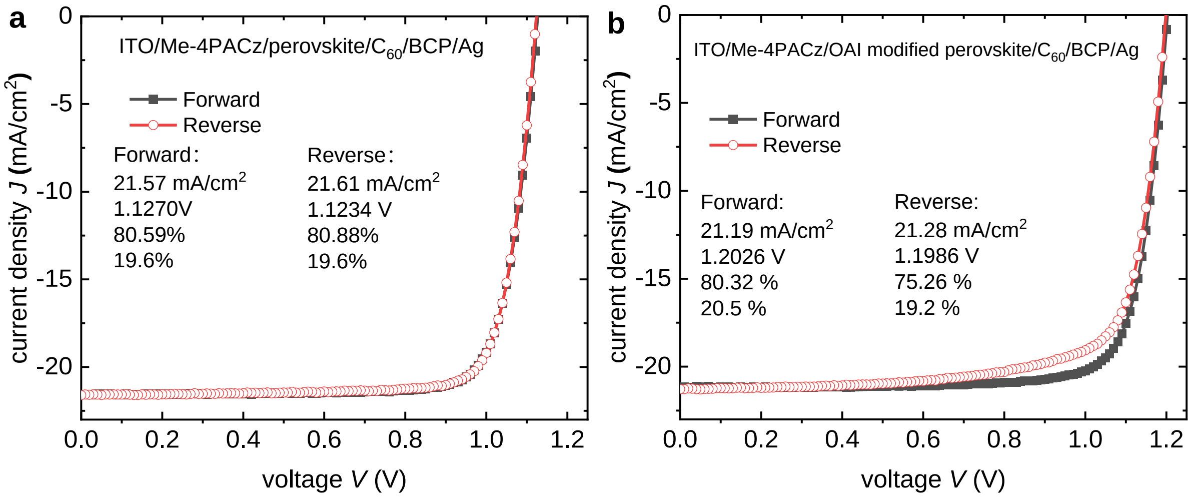

Supplementary Fig. 38 *JV* curves of **a** control and **b** OAI modified solar cells under both forward and reverse scans.

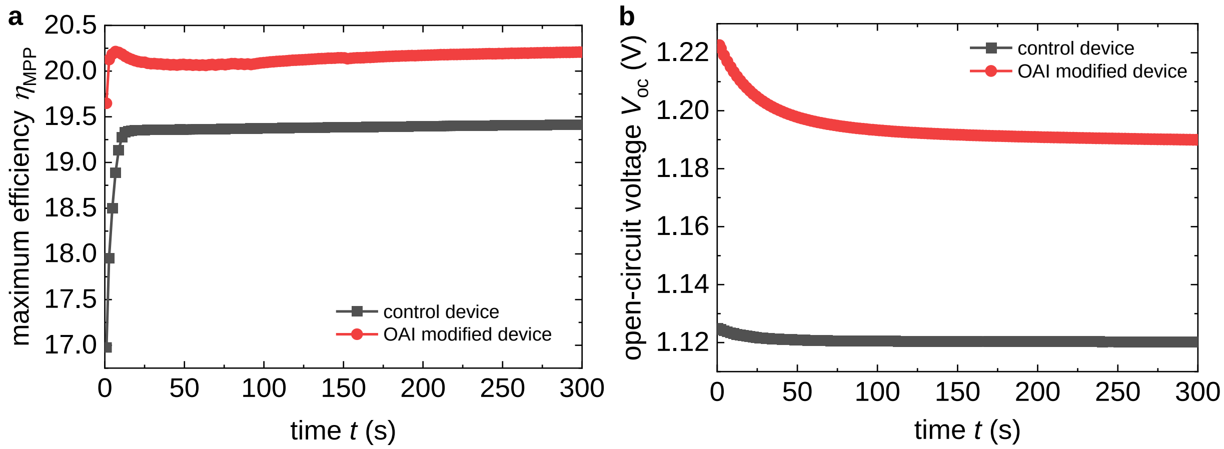

Supplementary Fig. 39 a Maximum efficiency tracking and **b** open-circuit voltage tracking of control and OAI modified devices under white LED with 1 sun intensity.

Supplementary Fig. 40 shows visualized comparison of potential improvement for different samples. The efficiency losses are broken down into five figure of merits (FoMs) via the following equation,[29, 30]

$$\frac{\eta^{\mathrm{real}}}{\eta^{\mathrm{SQ}}} = F_{\mathrm{sc}} \frac{V_{\mathrm{oc}}^{\mathrm{real}}}{V_{\mathrm{oc}}^{\mathrm{rad}}} \frac{V_{\mathrm{oc}}^{\mathrm{rad}}}{V_{\mathrm{oc}}^{\mathrm{SQ}}} \frac{\mathrm{FF}_0(V_{\mathrm{oc}}^{\mathrm{real}})}{\mathrm{FF}_0(V_{\mathrm{oc}}^{\mathrm{SQ}})} F_{\mathrm{FF}}^{\mathrm{res}} \tag{39}$$

where $F_{sc} = J_{sc}/J_{sc}^{SQ}$, represents photocurrent loss; $\mathrm{FF}_0(V_{oc}^{real})$/ $\mathrm{FF}_0(V_{oc}^{SQ})$ corresponds to FF loss due to the loss in V_{oc}; $F_{FF}^{res} = \mathrm{FF}_{real}/\,\mathrm{FF}_0(V_{oc}^{real})$ relates to FF loss caused by the resistive and ideality factor; $V_{oc}^{real}/V_{oc}^{rad}$ represents V_{oc} loss due to the nonradiative recombination; V_{oc}^{rad}/V_{oc}^{SQ} represents V_{oc} loss due to discrepancy between the actual absorption coefficient and the assumed step-function in SQ limit. In the figure, we calculate the efficiency loss for three devices: the control sample, the OAI modified sample with PCE ~ 20.5% (named OAI A) and the OAI modified sample with the highest open-circuit voltage over 1.23 V (named OAI B). It shows that nonradiative recombination ($V_{oc}^{real}/V_{oc}^{rad}$) contribute the main share for the loss of control sample. With OAI modification, the green bar gets shorter, indicating less nonradiative recombination loss, resulting in final η^{real}/η^{SQ} ratio increase. However, proportions of photocurrent loss and FF loss increase a bit due to the resistive and ideality factor problem.

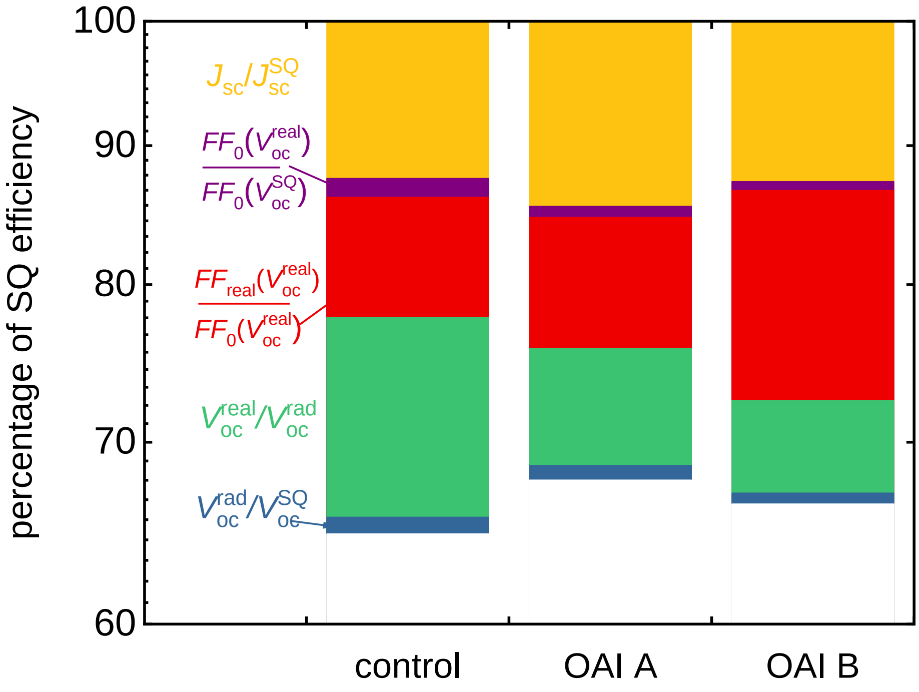

Supplementary Fig. 40 Visualization of potential improvement using FoMs of the efficiency losses for control device and two representative OAI modified devices (named OAI A and OAI B).

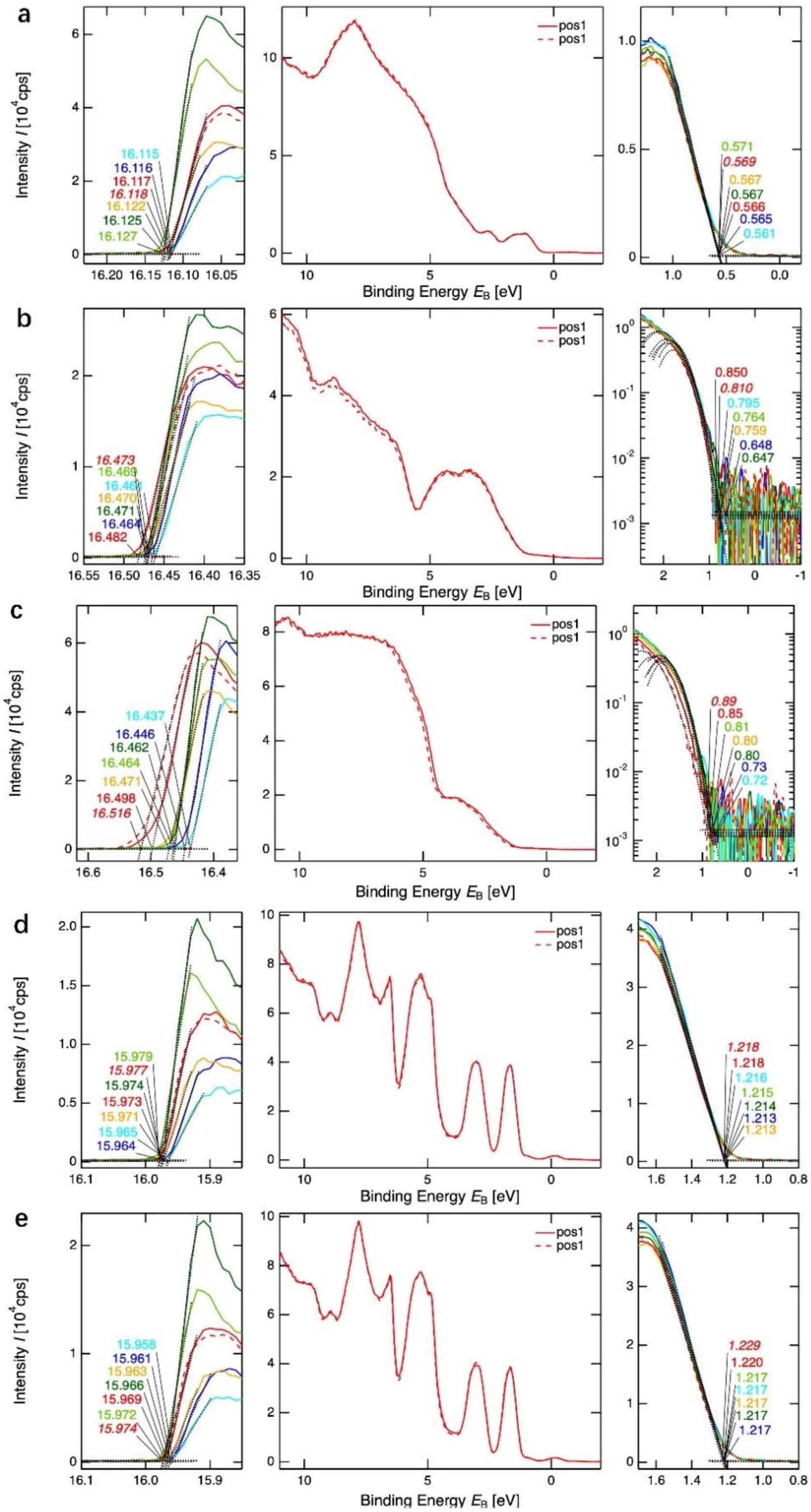

Supplementary Fig. 41 Original UPS spectra of **a** Me-4PACz, **b** pure perovskite, **c** OAI modified perovskite, **d** C_{60} prepared on pure perovskite and **e** C_{60} prepared on OAI modified perovskite.

To visualize energy-level diagrams of devices, we have performed UPS measurement for each layer. Each sample was measured for 7 times to improve the accuracy (as shown in Supplementary Fig. 41). The median values were used to plot the band diagram. Supplementary Fig. 42a and b illustrates the energy-levels of the control and OAI treated devices relative to the vacuum level. Additionally, the equilibrium-band diagram of both device samples which simulated by SCAPS is shown in Supplementary Fig. 42c and d. There is an obvious conduction band offset of ~300 meV at the perovskite/C_{60} interface, which would be adverse to quasi Fermi-level splitting. After the OAI treatment, the band offset decreases to 250 meV, facilitating the open-circuit voltage.

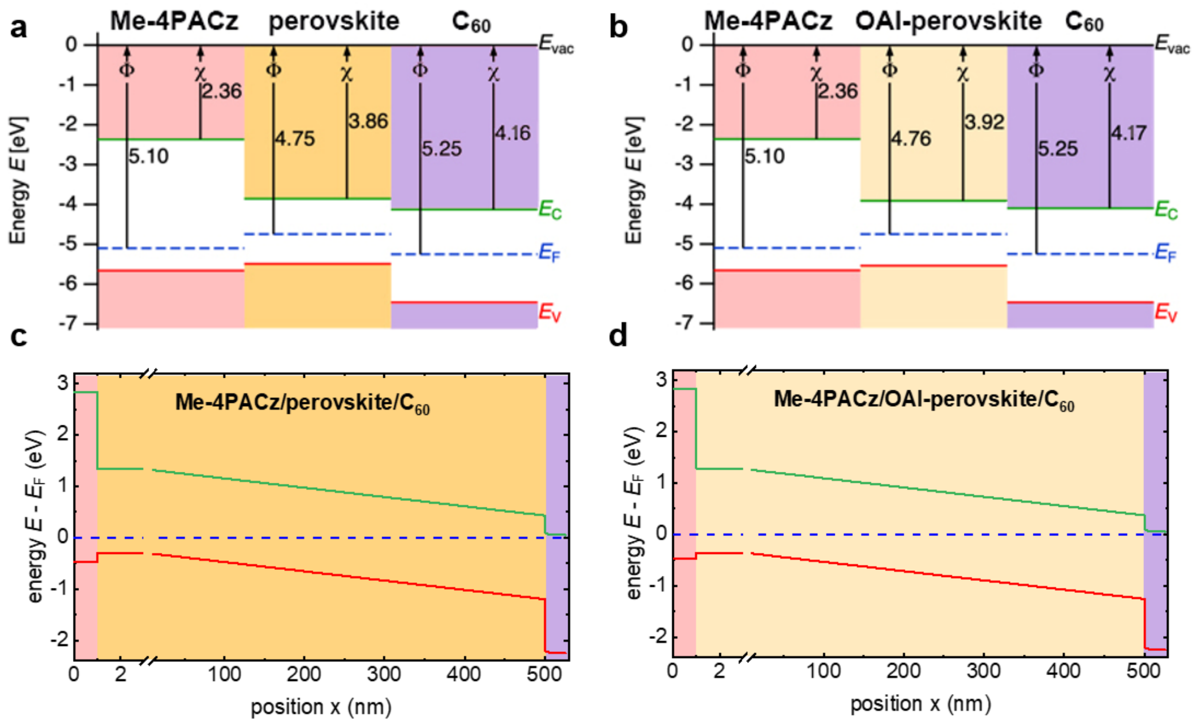

Supplementary Fig. 42 Schematics of the energy-level diagrams of **a** control and **b** OAI modified samples. **c,d** The corresponding equilibrium-band diagrams simulated by SCAPS.

Supplementary Note 5 References used for Fig. 1 in the main paper

Supplementary Table 3 Detailed parameters and corresponding references used for Fig. 1 in the main paper.

τ (s)	E_g/q- V_{oc} (V)	Dynamic range (orders of magnitude)	Fitting methods	Ref.
1×10^{-6}	0.486	3	mono-exponential	[31]
1.82×10^{-5}	0.442	3	mono-exponential	[31]
1.8×10^{-6}	N/A	4	differential	[27]
5.1×10^{-7}	N/A	5	differential	[32]
8.5×10^{-7}	N/A	4	N/A	[33]
9.7×10^{-7}	N/A	3.5	stretched exponential	[34]
8.8×10^{-6}	N/A	3.5	stretched exponential	[34]
5.5×10^{-6}	N/A	3	stretched exponential	[35]
3.5×10^{-7}	N/A	3	stretched exponential	[35]
1×10^{-6}	0.39	3	mono-exponential	[36]
1.5×10^{-6}	N/A	3.5	mono-exponential	[36]
8.6×10^{-7}	0.412	3.5	mono-exponential	[37]
2×10^{-6}	0.41	6	mono-exponential	[37]
N/A	0.438	4.5	N/A	[38]
N/A	0.51	1	N/A	[38]
1.39×10^{-7}	0.416	1	bi-exponential	[39]
1.23×10^{-6}	0.43	4	bi-exponential	[39]
3.64×10^{-7}	0.44	4	N/A	[40]
2.8×10^{-8}	0.61	3	mono-exponential	[41]
4.5×10^{-8}	0.68	3	mono-exponential	[41]
5.5×10^{-7}	0.42	3	mono-exponential	[42]
1.37×10^{-6}	0.4	3	mono-exponential	[42]
1.33×10^{-6}	0.41	3	mono-exponential	[42]
1.93×10^{-6}	0.41	3	mono-exponential	[42]
4.3×10^{-9}	0.69	2	mono-exponential	[43]
2.1×10^{-9}	0.8	2	mono-exponential	[43]

2.15×10^{-7}	0.5	2	bi-exponential	[44]
6.27×10^{-7}	0.4	2	mono-exponential	[44]
7.71×10^{-7}	0.5	1	bi-exponential	[45]
1.22×10^{-6}	0.47	1	bi-exponential	[45]
2.66×10^{-6}	0.42	1	bi-exponential	[45]
1.85×10^{-6}	0.42	1	bi-exponential	[45]
1.35×10^{-6}	0.38	1	bi-exponential	[45]
2.67×10^{-7}	0.5	1	bi-exponential	[46]
2.45×10^{-7}	0.48	1	bi-exponential	[46]
5.56×10^{-7}	0.46	1	bi-exponential	[46]
4.91×10^{-7}	0.46	1	bi-exponential	[46]
7.37×10^{-7}	0.43	1	bi-exponential	[46]
9.3×10^{-7}	0.42	1	bi-exponential	[46]
7.81×10^{-7}	0.43	1	bi-exponential	[46]
1.28×10^{-6}	0.43	1	bi-exponential	[46]
N/A	0.421	2	mono-exponential	[47]
N/A	0.442	2	mono-exponential	[47]
N/A	0.474	2	mono-exponential	[47]
3.43×10^{-7}	0.421	2	mono-exponential	[47]
1.5×10^{-7}	0.512	2	bi-exponential	[48]
1.6×10^{-7}	0.493	2	bi-exponential	[48]
2.1×10^{-7}	0.475	2	bi-exponential	[48]
3.5×10^{-7}	0.47	2	bi-exponential	[48]
5.8×10^{-7}	0.454	2	bi-exponential	[48]
8×10^{-7}	0.45	2	bi-exponential	[48]
9.2×10^{-7}	0.445	2	bi-exponential	[48]
7.5×10^{-7}	0.37	4.5	differential	[24]
4×10^{-5}	N/A	7.5	differential	[5]
7×10^{-6}	0.53	2	differential	[49]
5×10^{-7}	0.429	2	differential	[50]
6.21×10^{-6}	0.39	2.5	mono-exponential	[51]
3.13×10^{-7}	0.46	2.5	mono-exponential	[51]
1.59×10^{-7}	0.4	1	biexponential	[52]
4×10^{-6}	0.341	1	mono-exponential	[53]

7.37×10^{-7}	0.171	2	bi-exponential	[54]
7.6×10^{-7}	0.469	N/A	N/A	[28]
3.66×10^{-6}	0.366	3	mono-exponential	[55]
4.62×10^{-7}	0.46	N/A	N/A	[20]
7.06×10^{-7}	0.36	N/A	N/A	[20]
2.02×10^{-7}	0.452	3	mono-exponential	[56]
1.11×10^{-6}	N/A	3	mono-exponential	[56]
6.56×10^{-7}	0.397	3	bi-exponential	[57]
9.56×10^{-7}	N/A	3	bi-exponential	[57]
1.18×10^{-6}	N/A	3	bi-exponential	[57]
6×10^{-7}	0.36	3	bi-exponential	[58]
N/A	0.31	2	mono-exponential	[59]
1.60×10^{-6}	0.399	2	bi-exponential	[60]
1.36×10^{-7}	0.41	2	bi-exponential	[60]
5.98×10^{-7}	0.403	2	bi-exponential	[60]
3.57×10^{-7}	0.497	3	bi-exponential	[60]
3.10×10^{-7}	0.508	1.5	mono-exponential	[61]
9.41×10^{-7}	N/A	1.5	mono-exponential	[61]
1.37×10^{-7}	0.5	1	bi-exponential	[62]
8.41×10^{-6}	0.75	3	mono-exponential	[63]
3.12×10^{-7}	N/A	3	N/A	[64]
9.82×10^{-9}	N/A	3	N/A	[64]
8.42×10^{-7}	0.401	2	bi-exponential	[65]
3.82×10^{-7}	0.475	2	bi-exponential	[65]
2.8×10^{-4}	0.398	10	differential	This work

Supplementary Note 6 Numerical models

Numerical simulations for the transient (Tr-PL) and steady-state photoluminescence (ss-PL) were performed using self-developed MATLAB scripts based on the coupled rate equations.

Equations that were solved for tr-PL numerical simulation encompass the time derivatives for the electron and holes concentrations n, p as well as for the concentrations $n_{\mathrm{t}x}$ $(x = 1,2,3)$ of occupied defects.

$$\frac{dn}{dt} = -k_{\mathrm{rad}}\left(np - n_i^2\right) - \beta_{\mathrm{n}}^{\mathrm{t1}} n (N_{\mathrm{t1}} - n_{\mathrm{t1}}) + e_{\mathrm{n}}^{\mathrm{t1}} n_{\mathrm{t1}} - \beta_{\mathrm{n}}^{\mathrm{t2}} n (N_{\mathrm{t2}} - n_{\mathrm{t2}}) + e_{\mathrm{n}}^{\mathrm{t2}} n_{\mathrm{t2}} - \beta_{n}^{\mathrm{t3}} n (N_{\mathrm{t3}} - n_{\mathrm{t3}}) + e_{\mathrm{n}}^{\mathrm{t3}} n_{\mathrm{t3}} + G_{\mathrm{bias}} \quad (40)$$

$$\frac{dp}{dt} = -k_{\mathrm{rad}}\left(np - n_i^2\right) - \beta_{\mathrm{p}}^{\mathrm{t1}} p n_{\mathrm{t1}} + e_{\mathrm{p}}^{\mathrm{t1}} (N_{\mathrm{t1}} - n_{\mathrm{t1}}) - \beta_{\mathrm{p}}^{\mathrm{t2}} p n_{\mathrm{t2}} + e_{\mathrm{p}}^{\mathrm{t2}} (N_{\mathrm{t2}} - n_{\mathrm{t2}}) - \beta_{\mathrm{p}}^{\mathrm{t3}} p n_{\mathrm{t3}} + e_{\mathrm{p}}^{\mathrm{t3}} (N_{\mathrm{t3}} - n_{\mathrm{t3}}) + G_{\mathrm{bias}} \quad (41)$$

$$\frac{dn_{\mathrm{t1}}}{dt} = \beta_{\mathrm{n}}^{\mathrm{t1}} n (N_{\mathrm{t1}} - n_{\mathrm{t1}}) - \beta_{\mathrm{p}}^{\mathrm{t1}} p n_{\mathrm{t1}} - e_{\mathrm{n}}^{\mathrm{t1}} n_{\mathrm{t1}} + e_{\mathrm{p}}^{\mathrm{t1}} \, (N_{\mathrm{t1}} - n_{\mathrm{t1}}) \quad (42)$$

$$\frac{dn_{\mathrm{t2}}}{dt} = \beta_{\mathrm{n}}^{\mathrm{t2}} n (N_{\mathrm{t2}} - n_{\mathrm{t2}}) - \beta_{\mathrm{p}}^{\mathrm{t2}} p n_{\mathrm{t2}} - e_{\mathrm{n}}^{\mathrm{t2}} n_{\mathrm{t2}} + e_{\mathrm{p}}^{\mathrm{t2}} (N_{\mathrm{t2}} - n_{\mathrm{t2}}) \quad (43)$$

$$\frac{dn_{\mathrm{t3}}}{dt} = \beta_{\mathrm{n}}^{\mathrm{t3}} n (N_{\mathrm{t3}} - n_{\mathrm{t3}}) - \beta_{\mathrm{p}}^{\mathrm{t3}} p n_{\mathrm{t3}} - e_{\mathrm{n}}^{\mathrm{t3}} n_{\mathrm{t3}} + e_{\mathrm{p}}^{\mathrm{t3}} (N_{\mathrm{t3}} - n_{\mathrm{t3}}) \quad (44)$$

Note that because of the over-all conservation of the electrical charge we have

$$\frac{dn}{dt} + \frac{dn_{\mathrm{t1}}}{dt} + \frac{dn_{\mathrm{t2}}}{dt} + \frac{dn_{\mathrm{t3}}}{dt} = \frac{dp}{dt} \quad (45)$$

such that a numerical solution of three differential equations is sufficient. For this numerical solution we use a Runge-Kutta algorithm as implemented in MATLAB with the initial conditions $n(0) = p(0) = n_i \exp(\Delta E_{\mathrm{F}}(0)/2k_{\mathrm{B}}T)$ and $n_{\mathrm{t}\,1/2/3}(0) = 0$ at time $t = 0$.

Equations that were solved for ss-PL numerical simulation are shown as below,

$$f_{\mathrm{SRH}}^{\mathrm{t1}} = (n\beta_{\mathrm{n}}^{\mathrm{t1}} + e_{\mathrm{p}}^{\mathrm{t1}})/(n\beta_{\mathrm{n}}^{\mathrm{t1}} + p\beta_{\mathrm{p}}^{\mathrm{t1}} + e_{\mathrm{n}}^{\mathrm{t1}} + e_{\mathrm{p}}^{\mathrm{t1}}) \quad (46)$$

$$f_{\mathrm{SRH}}^{\mathrm{t2}} = (n\beta_{\mathrm{n}}^{\mathrm{t2}} + e_{\mathrm{p}}^{\mathrm{t2}})/(n\beta_{\mathrm{n}}^{\mathrm{t2}} + p\beta_{\mathrm{p}}^{\mathrm{t2}} + e_{\mathrm{n}}^{\mathrm{t2}} + e_{\mathrm{p}}^{\mathrm{t2}}) \quad (47)$$

$$f_{\mathrm{SRH}}^{\mathrm{t3}} = (n\beta_{\mathrm{n}}^{\mathrm{t3}} + e_{\mathrm{p}}^{\mathrm{t3}})/(n\beta_{\mathrm{n}}^{\mathrm{t3}} + p\beta_{\mathrm{p}}^{\mathrm{t3}} + e_{\mathrm{n}}^{\mathrm{t3}} + e_{\mathrm{p}}^{\mathrm{t3}}) \quad (48)$$

Where $f_{\mathrm{SRH}}^{\mathrm{t1}}$, $f_{\mathrm{SRH}}^{\mathrm{t2}}$ and $f_{\mathrm{SRH}}^{\mathrm{t3}}$are the occupation probability of Defect 1, Defect 2 and Defect 3.

$$n_{\mathrm{t1}} = f_{\mathrm{SRH}}^{\mathrm{t1}} \times N_{\mathrm{t1}} \quad (49)$$

$$n_{\mathrm{t2}} = f_{\mathrm{SRH}}^{\mathrm{t2}} \times N_{\mathrm{t2}} \quad (50)$$

$$n_{\mathrm{t3}} = f_{\mathrm{SRH}}^{\mathrm{t3}} \times N_{\mathrm{t3}} \quad (51)$$

Then the radiative recombination rate R_{rad}, the SRH recombination rates $R_{\mathrm{SRH}}^{\mathrm{t1}}$, $R_{\mathrm{SRH}}^{\mathrm{t2}}$, $R_{\mathrm{SRH}}^{\mathrm{t3}}$ and the total recombination rate R_{tot} can be calculated,

$$R_{\mathrm{rad}} = k_{\mathrm{rad}} np \quad (52)$$

$$R_{\mathrm{SRH}}^{\mathrm{t1}} = N_{\mathrm{t1}}\beta_{\mathrm{n}}^{\mathrm{t1}}\beta_{\mathrm{p}}^{\mathrm{t1}}(np - n_i^2)/(n\beta_{\mathrm{n}}^{\mathrm{t1}} + p\beta_{\mathrm{p}}^{\mathrm{t1}} + e_{\mathrm{n}}^{\mathrm{t1}} + e_{\mathrm{p}}^{\mathrm{t1}}) \quad (53)$$

$$R_{\mathrm{SRH}}^{\mathrm{t2}} = N_{\mathrm{t2}}\beta_{\mathrm{n}}^{\mathrm{t2}}\beta_{\mathrm{p}}^{\mathrm{t2}}(np - n_i^2)/(n\beta_{\mathrm{n}}^{\mathrm{t2}} + p\beta_{\mathrm{p}}^{\mathrm{t2}} + e_{\mathrm{n}}^{\mathrm{t2}} + e_{\mathrm{p}}^{\mathrm{t2}}) \quad (54)$$

$$R_{\mathrm{SRH}}^{\mathrm{t3}} = N_{\mathrm{t3}}\beta_{\mathrm{n}}^{\mathrm{t3}}\beta_{\mathrm{p}}^{\mathrm{t3}}(np - n_i^2)/(n\beta_{\mathrm{n}}^{\mathrm{t3}} + p\beta_{\mathrm{p}}^{\mathrm{t3}} + e_{\mathrm{n}}^{\mathrm{t3}} + e_{\mathrm{p}}^{\mathrm{t3}}) \quad (55)$$

$$R_{\mathrm{tot}} = R_{\mathrm{rad}} + R_{\mathrm{SRH}}^{\mathrm{t1}} + R_{\mathrm{SRH}}^{\mathrm{t2}} + R_{\mathrm{SRH}}^{\mathrm{t3}} \quad (56)$$

$$p = n + n_{\mathrm{t1}} + n_{\mathrm{t2}} + n_{\mathrm{t3}} \quad (57)$$

Iteration process is used in the script. It ends until *p* no long changed and thus we acquire the final results.

References

1. Shockley W, Read WT. Statistics of the Recombinations of Holes and Electrons. *Physical Review* 1952, **87**(5)**:** 835-842.

2. Siekmann J, Kulkarni A, Akel S, Klingebiel B, Saliba M, Rau U*, et al.* Characterizing the Influence of Charge Extraction Layers on the Performance of Triple-Cation Perovskite Solar Cells. *Adv Energy Mater*, **n/a**(n/a)**:** 2300448.

3. Wolff CM, Zu F, Paulke A, Toro LP, Koch N, Neher D. Reduced Interface-Mediated Recombination for High Open-Circuit Voltages in CH3NH3PbI3 Solar Cells. *Adv. Mater.* 2017, **29**(28)**:** 1700159.

4. Vandewal K, Tvingstedt K, Gadisa A, Inganäs O, Manca JV. Relating the open-circuit voltage to interface molecular properties of donor:acceptor bulk heterojunction solar cells. *Physical Review B* 2010, **81**(12)**:** 125204.

5. Krückemeier L, Krogmeier B, Liu Z, Rau U, Kirchartz T. Understanding Transient Photoluminescence in Halide Perovskite Layer Stacks and Solar Cells. *Adv Energy Mater* 2021, **11**(19)**:** 2003489.

6. Peña-Camargo F, Thiesbrummel J, Hempel H, Musiienko A, Corre VML, Diekmann J*, et al.* Revealing the doping density in perovskite solar cells and its impact on device performance. *Applied Physics Reviews* 2022, **9**(2)**:** 021409.

7. Hornbeck JA, Haynes JR. Trapping of Minority Carriers in Silicon. I. P-Type Silicon. *Physical Review* 1955, **97**(2)**:** 311-321.

8. Haynes JR, Hornbeck JA. Temporary Traps in Silicon and Germanium. *Physical Review* 1953, **90**(1)**:** 152-153.

9. Haynes JR, Hornbeck JA. Trapping of Minority Carriers in Silicon. II. n-Type Silicon. *Physical Review* 1955, **100**(2)**:** 606-615.

10. Macdonald DH. Recombination and Trapping in Multicrystalline Silicon Solar Cells. Doctor of Philosophy thesis, The Australian National University, 2001.

11. Brandt RE, Stevanović V, Ginley DS, Buonassisi T. Identifying defect-tolerant semiconductors with high minority-carrier lifetimes: beyond hybrid lead halide perovskites. *MRS Communications* 2015, **5**(2)**:** 265-275.

12. Das B, Liu Z, Aguilera I, Rau U, Kirchartz T. Defect tolerant device geometries for lead-halide perovskites.

Materials Advances 2021, **2**(11)**:** 3655-3670.

13. Huang H, Bodnarchuk MI, Kershaw SV, Kovalenko MV, Rogach AL. Lead Halide Perovskite Nanocrystals in the Research Spotlight: Stability and Defect Tolerance. *ACS Energy Lett* 2017, **2**(9)**:** 2071-2083.

14. Zhang X, Turiansky ME, Van de Walle CG. Correctly Assessing Defect Tolerance in Halide Perovskites. *The Journal of Physical Chemistry C* 2020, **124**(11)**:** 6022-6027.

15. Barugkin C, Cong J, Duong T, Rahman S, Nguyen HT, Macdonald D*, et al.* Ultralow Absorption Coefficient and Temperature Dependence of Radiative Recombination of CH3NH3PbI3 Perovskite from Photoluminescence. *The Journal of Physical Chemistry Letters* 2015, **6**(5)**:** 767-772.

16. Henry CH, Lang DV. Nonradiative capture and recombination by multiphonon emission in GaAs and GaP. *Physical Review B* 1977, **15**(2)**:** 989-1016.

17. Markvart T. Multiphonon recombination. In: Landsberg PT (ed). *Recombination in Semiconductors*. Cambridge University Press: Cambridge, 2003, p 470.

18. Markvart T. Semiclassical theory of non-radiative transitions. *Journal of Physics C: Solid State Physics* 1981, **14**(29)**:** L895.

19. Zhang X, Shen J-X, Turiansky ME, Van de Walle CG. Minimizing hydrogen vacancies to enable highly efficient hybrid perovskites. *Nature Materials* 2021.

20. Li X, Zhang W, Guo X, Lu C, Wei J, Fang J. Constructing heterojunctions by surface sulfidation for efficient inverted perovskite solar cells. *Science* 2022, **375**(6579)**:** 434-437.

21. Degani M, An Q, Albaladejo-Siguan M, Hofstetter YJ, Cho C, Paulus F*, et al.* 23.7% Efficient inverted perovskite solar cells by dual interfacial modification. *Sci Adv* 2021, **7**(49)**:** eabj7930.

22. Rau U, Blank B, Müller TCM, Kirchartz T. Efficiency Potential of Photovoltaic Materials and Devices Unveiled by Detailed-Balance Analysis. *Physical Review Applied* 2017, **7**(4)**:** 044016.

23. Krückemeier L, Rau U, Stolterfoht M, Kirchartz T. How to Report Record Open-Circuit Voltages in Lead-Halide Perovskite Solar Cells. *Adv Energy Mater* 2020, **10**(1)**:** 1902573.

24. Liu Z, Siekmann J, Klingebiel B, Rau U, Kirchartz T. Interface Optimization via Fullerene Blends Enables Open-Circuit Voltages of 1.35 V in $CH_3NH_3Pb(I_{0.8}Br_{0.2})_3$ Solar Cells. *Adv Energy Mater* 2021, **11**(16)**:** 2003386.

25. Almora O, Baran D, Bazan GC, Berger C, Cabrera CI, Catchpole KR*, et al.* Device Performance of Emerging

Photovoltaic Materials (Version 2). *Adv Energy Mater* 2021, **11**(48)**:** 2102526.

26. Al-Ashouri A, Kohnen E, Li B, Magomedov A, Hempel H, Caprioglio P*, et al.* Monolithic perovskite/silicon tandem solar cell with > 29% efficiency by enhanced hole extraction. *Science* 2020, **370**(6522)**:** 1300-1309.

27. Liu Z, Krückemeier L, Krogmeier B, Klingebiel B, Márquez JA, Levcenko S*, et al.* Open-Circuit Voltages Exceeding 1.26 V in Planar Methylammonium Lead Iodide Perovskite Solar Cells. *ACS Energy Lett.* 2019, **4**(1)**:** 110-117.

28. Yang G, Ren Z, Liu K, Qin M, Deng W, Zhang H*, et al.* Stable and low-photovoltage-loss perovskite solar cells by multifunctional passivation. *Nat Photonics* 2021, **15**(9)**:** 681-689.

29. Guillemoles JF, Kirchartz T, Cahen D, Rau U. Guide for the perplexed to the Shockley-Queisser model for solar cells. *Nat Photonics* 2019, **13**(8)**:** 501-505.

30. Guillemoles J-F, Kirchartz T, Cahen D, Rau U. Reply to 'Ideal solar cell efficiencies'. *Nat Photonics* 2021, **15**(3)**:** 165-166.

31. Gutierrez-Partida E, Hempel H, Caicedo-Dávila S, Raoufi M, Peña-Camargo F, Grischek M*, et al.* Large-Grain Double Cation Perovskites with 18 μs Lifetime and High Luminescence Yield for Efficient Inverted Perovskite Solar Cells. *ACS Energy Lett.* 2021, **6**(3)**:** 1045-1054.

32. Staub F, Hempel H, Hebig J-C, Mock J, Paetzold UW, Rau U*, et al.* Beyond Bulk Lifetimes: Insights into Lead Halide Perovskite Films from Time-Resolved Photoluminescence. *Physical Review Applied* 2016, **6**(4)**:** 044017.

33. Richter JM, Abdi-Jalebi M, Sadhanala A, Tabachnyk M, Rivett JPH, Pazos-Outón LM*, et al.* Enhancing photoluminescence yields in lead halide perovskites by photon recycling and light out-coupling. *Nature Communications* 2016, **7**(1)**:** 13941.

34. deQuilettes DW, Koch S, Burke S, Paranji RK, Shropshire AJ, Ziffer ME*, et al.* Photoluminescence Lifetimes Exceeding 8 μs and Quantum Yields Exceeding 30% in Hybrid Perovskite Thin Films by Ligand Passivation. *ACS Energy Lett.* 2016, **1**(2)**:** 438-444.

35. Braly IL, deQuilettes DW, Pazos-Outón LM, Burke S, Ziffer ME, Ginger DS*, et al.* Hybrid perovskite films approaching the radiative limit with over 90% photoluminescence quantum efficiency. *Nat Photonics* 2018, **12**(6)**:** 355-361.

36. Abdi-Jalebi M, Andaji-Garmaroudi Z, Cacovich S, Stavrakas C, Philippe B, Richter JM*, et al.* Maximizing and stabilizing luminescence from halide perovskites with potassium passivation. *Nature* 2018, **555**(7697)**:** 497-501.

37. Al-Ashouri A, Magomedov A, Roß M, Jošt M, Talaikis M, Chistiakova G*, et al.* Conformal monolayer contacts with lossless interfaces for perovskite single junction and monolithic tandem solar cells. *Energ Environ Sci* 2019, **12**(11)**:** 3356-3369.

38. Abdi-Jalebi M, Pazoki M, Philippe B, Dar MI, Alsari M, Sadhanala A*, et al.* Dedoping of Lead Halide Perovskites Incorporating Monovalent Cations. *ACS Nano* 2018, **12**(7)**:** 7301-7311.

39. Tong J, Song Z, Kim DH, Chen X, Chen C, Palmstrom AF*, et al.* Carrier lifetimes of >1 μs in Sn-Pb perovskites enable efficient all-perovskite tandem solar cells. *Science* 2019, **364**(6439)**:** 475-479.

40. Jiang Q, Zhao Y, Zhang X, Yang X, Chen Y, Chu Z*, et al.* Surface passivation of perovskite film for efficient solar cells. *Nat Photonics* 2019, **13**(7)**:** 460-466.

41. Song D, Cui P, Wang T, Wei D, Li M, Cao F*, et al.* Managing Carrier Lifetime and Doping Property of Lead Halide Perovskite by Postannealing Processes for Highly Efficient Perovskite Solar Cells. *The Journal of Physical Chemistry C* 2015, **119**(40)**:** 22812-22819.

42. Turren-Cruz S-H, Saliba M, Mayer MT, Juárez-Santiesteban H, Mathew X, Nienhaus L*, et al.* Enhanced charge carrier mobility and lifetime suppress hysteresis and improve efficiency in planar perovskite solar cells. *Energ Environ Sci* 2018, **11**(1)**:** 78-86.

43. Nie W, Tsai H, Asadpour R, Blancon J-C, Neukirch AJ, Gupta G*, et al.* High-efficiency solution-processed perovskite solar cells with millimeter-scale grains. *Science* 2015, **347**(6221)**:** 522-525.

44. Alharbi EA, Dar MI, Arora N, Alotaibi MH, Alzhrani YA, Yadav P*, et al.* Perovskite Solar Cells Yielding Reproducible Photovoltage of 1.20 V. *Research* 2019, **2019:** 8474698.

45. Wu S, Li Z, Zhang J, Liu T, Zhu Z, Jen AKY. Efficient large guanidinium mixed perovskite solar cells with enhanced photovoltage and low energy losses. *Chem Commun* 2019, **55**(30)**:** 4315-4318.

46. Yang S, Dai J, Yu Z, Shao Y, Zhou Y, Xiao X*, et al.* Tailoring Passivation Molecular Structures for Extremely Small Open-Circuit Voltage Loss in Perovskite Solar Cells. *Journal of the American Chemical Society* 2019, **141**(14)**:** 5781-5787.

47. Wang Q, Mosconi E, Wolff C, Li J, Neher D, De Angelis F*, et al.* Rationalizing the Molecular Design of Hole-Selective Contacts to Improve Charge Extraction in Perovskite Solar Cells. *Adv Energy Mater* 2019, **9**(28)**:** 1900990.

48. Caprioglio P, Zu F, Wolff CM, Márquez Prieto JA, Stolterfoht M, Becker P*, et al.* High open circuit voltages in pin-type perovskite solar cells through strontium addition. *Sustain. Energ. Fuels* 2019, **3**(2)**:** 550-563.

49. Al-Ashouri A, Köhnen E, Li B, Magomedov A, Hempel H, Caprioglio P*, et al.* Monolithic perovskite/silicon tandem solar cell with >29% efficiency by enhanced hole extraction. *Science* 2020, **370**(6522)**:** 1300-1309.

50. Ding Y, Ding B, Kanda H, Usiobo OJ, Gallet T, Yang Z*, et al.* Single-crystalline TiO2 nanoparticles for stable and efficient perovskite modules. *Nature Nanotechnology* 2022, **17**(6)**:** 598-605.

51. Yang X, Fu Y, Su R, Zheng Y, Zhang Y, Yang W*, et al.* Superior Carrier Lifetimes Exceeding 6 µs in Polycrystalline Halide Perovskites. *Adv. Mater.* 2020, **32**(39)**:** 2002585.

52. Lin R, Xu J, Wei M, Wang Y, Qin Z, Liu Z*, et al.* All-perovskite tandem solar cells with improved grain surface passivation. *Nature* 2022, **603**(7899)**:** 73-78.

53. Su R, Xu Z, Wu J, Luo D, Hu Q, Yang W*, et al.* Dielectric screening in perovskite photovoltaics. *Nature Communications* 2021, **12**(1)**:** 2479.

54. Min H, Lee DY, Kim J, Kim G, Lee KS, Kim J*, et al.* Perovskite solar cells with atomically coherent interlayers on SnO2 electrodes. *Nature* 2021, **598**(7881)**:** 444-450.

55. Yoo JJ, Seo G, Chua MR, Park TG, Lu Y, Rotermund F*, et al.* Efficient perovskite solar cells via improved carrier management. *Nature* 2021, **590**(7847)**:** 587-593.

56. Jang Y-W, Lee S, Yeom KM, Jeong K, Choi K, Choi M*, et al.* Intact 2D/3D halide junction perovskite solar cells via solid-phase in-plane growth. *Nat. Energy* 2021, **6**(1)**:** 63-71.

57. Chen J, Yang Y, Dong H, Li J, Zhu X, Xu J*, et al.* Highly efficient and stable perovskite solar cells enabled by low-dimensional perovskitoids. *Science Advances* 2022, **8**(4)**:** eabk2722.

58. Peng J, Walter D, Ren Y, Tebyetekerwa M, Wu Y, Duong T*, et al.* Nanoscale localized contacts for high fill factors in polymer-passivated perovskite solar cells. *Science* 2021, **371**(6527)**:** 390-395.

59. Kim M, Jeong J, Lu H, Lee TK, Eickemeyer FT, Liu Y*, et al.* Conformal quantum dot-SnO2 layers as electron transporters for efficient perovskite solar cells. *Science* 2022, **375**(6578)**:** 302-306.

60. Li N, Niu X, Li L, Wang H, Huang Z, Zhang Y*, et al.* Liquid medium annealing for fabricating durable perovskite solar cells with improved reproducibility. *Science* 2021, **373**(6554)**:** 561-567.

61. Cao Q, Li YJ, Zhang H, Yang JB, Han J, Xu T*, et al.* Efficient and stable inverted perovskite solar cells with very high fill factors via incorporation of star-shaped polymer. *Science Advances* 2021, **7**(28).

62. Yang Z, Zhang W, Wu S, Zhu H, Liu Z, Liu Z*, et al.* Slot-die coating large-area formamidinium-cesium perovskite film for efficient and stable parallel solar module. *Science Advances* 2021, **7**(18)**:** eabg3749.

63. Liu Y, Zhang Y, Zhu X, Yang Z, Ke W, Feng J*, et al.* Inch-sized high-quality perovskite single crystals by suppressing phase segregation for light-powered integrated circuits. *Science Advances* 2021, **7**(7)**:** eabc8844.

64. Hui W, Chao L, Lu H, Xia F, Wei Q, Su Z*, et al.* Stabilizing black-phase formamidinium perovskite formation at room temperature and high humidity. *Science* 2021, **371**(6536)**:** 1359-1364.

65. Huang T, Tan S, Nuryyeva S, Yavuz I, Babbe F, Zhao Y*, et al.* Performance-limiting formation dynamics in mixed-halide perovskites. *Science Advances* 2021, **7**(46)**:** eabj1799.
