# Supplementary material for: Shallow defects and variable photoluminescence decay times up to 280 µs in triple-cation perovskites
Source: Nat Mater. 2024 Jan 9;23(3):391–7. doi: 10.1038/s41563-023-01771-2 (PMC10917677; doi:10.1038/s41563-023-01771-2)
Supplement: Supplementary file 2 — Reporting Summary [file 41563_2023_1771_MOESM2_ESM.pdf]

nature research

Corresponding author(s): Genghua Yan, Thomas Kirchartz

Solar Cells Reporting Summary

Nature Research wishes to improve the reproducibility of the work that we publish. This form is intended for publication with all accepted papers reporting the characterization of photovoltaic devices and provides structure for consistency and transparency in reporting. Some list items might not apply to an individual manuscript, but all fields must be completed for clarity.

For further information on Nature Research policies, including our data availability policy, see Authors & Referees.

Experimental design

Please check: are the following details reported in the manuscript?

1. Dimensions

Item	Yes	No	Details
Area of the tested solar cells	☒	☐	in Fig.4, in the Methods section and in Section 4 of the Supplementary Information
Method used to determine the device area	☒	☐	in the Methods section

2. Current-voltage characterization

Item	Yes	No	Details
Current density-voltage (J-V) plots in both forward and backward direction	☒	☐	in the Supplementary Fig.38
Voltage scan conditions *For instance: scan direction, speed, dwell times*	☒	☐	in the Methods section
Test environment *For instance: characterization temperature, in air or in glove box*	☒	☐	in the Methods section
Protocol for preconditioning of the device before its characterization	☒	☐	in the Methods section
Stability of the J-V characteristic *Verified with time evolution of the maximum power point or with the photocurrent at maximum power point; see ref. 7 for details.*	☒	☐	in the Supplementary Fig.39

3. Hysteresis or any other unusual behaviour

Item	Yes	No	Details
Description of the unusual behaviour observed during the characterization	☒	☐	in Section 4 of the Supplementary Information
Related experimental data	☒	☐	in Supplementary Fig.38

4. Efficiency

Item	Yes	No	Details
External quantum efficiency (EQE) or incident photons to current efficiency (IPCE)	☒	☐	in Section 4 of the Supplementary Information
A comparison between the integrated response under the standard reference spectrum and the response measure under the simulator	☒	☐	EQE integrated Jsc in Supplementary Fig.34 and simulator measured Jsc in Fig.4 and in Supplementary Fig.35, 36 and 38.
For tandem solar cells, the bias illumination and bias voltage used for each subcell	☐	☒	We don't have tandem cells.

5. Calibration

Item	Yes	No	Details
Light source and reference cell or sensor used for the characterization	☒	☐	in the Methods section
Confirmation that the reference cell was calibrated and certified	☒	☐	in the Methods section. The reference cell was certified by the Fraunhofer ISE, Germany.

November 2017

Calculation of spectral mismatch between the reference cell and the devices under test

☒ Yes ☐ No

in the Methods section. The mismatch factor is ~0.98.

6. Mask/aperture

Size of the mask/aperture used during testing

☐ Yes ☒ No

We didn't use mask during testing. It will lead to erroneous Voc and FF with masking though the determination of Jsc can be more accurate(Kiermasch et al. Joule 2019, 3(1): 16-26). So we consider that validating the Jsc with the EQE results and acquiring the correct Voc and FF without a mask is the best option.

Variation of the measured short-circuit current density with the mask/aperture area

☐ Yes ☒ No

We didn't use mask during testing. But we varied the active area of the device and acquired the Jsc (shown in Fig.4 and Supplementary Fig.38)

7. Performance certification

Identity of the independent certification laboratory that confirmed the photovoltaic performance

☐ Yes ☒ No

As we focus on studying the PL related physical mechanism in this work, we have not certified the efficiency value. We use a AAA class solar simulator for the efficiency measurement.

A copy of any certificate(s)
Provide in Supplementary Information

☐ Yes ☒ No

No certificates.

8. Statistics

Number of solar cells tested

☒ Yes ☐ No

in the Supplementary Fig.35

Statistical analysis of the device performance

☒ Yes ☐ No

in the Supplementary Fig.35

9. Long-term stability analysis

Type of analysis, bias conditions and environmental conditions
For instance: illumination type, temperature, atmosphere humidity, encapsulation method, preconditioning temperature

☐ Yes ☒ No

As we focus on studying the PL related physical mechanism in this work, we have not measured the long-term stability.
